# Supplementary material for: Distribution and population structure in the naked goby Gobiosoma bosc (Perciformes: Gobiidae) along a salinity gradient in two western Atlantic estuaries
Source: PeerJ. 2018 Aug 7;6:e5380. doi: 10.7717/peerj.5380 (PMC6086083; doi:10.7717/peerj.5380)
Supplement: Table S1 — In the table below, the first column represents the haplotype number (the actual sequence information for each haplotype follows this table). Columns 2–10 represent newly collected raw data from populations in North Carolina. These include the abundance of a particular haplotype per site. Columns 11–12 represent data extracted from Genbank that were part of Van Tassell et al. (2015). Columns 13–23 represent incidence data extracted from Genbank that were part of Mila et al. (2017). [file peerj-06-5380-s001.docx]

**Table S1. Haplotype data and sequence information**

In the table below, the first column represents the haplotype number (the actual sequence information for each haplotype follows this table). Columns 2-10 represent newly collected raw data from populations in North Carolina. These include the abundance of a particular haplotype per site. Columns 11-12 represent data extracted from Genbank that were part of Van Tassell et al. (2015). Columns 13-23 represent incidence data extracted from Genbank that were part of Mila et al. (2017). Below the table are the individual sequences for each of the 88 haplotypes represented in the table.

| HAP | GSC | MLC | NCL | WRC | FSL | CQC | MTP | POC | CDI | HPC | FL | GERM | MIDATL | SC | JAFL | IRFL | CKFL | TBFL | APFL | DEFL | EMLA | OSMS | GATX |
| --- | --- | --- | --- | --- | --- | --- | --- | --- | --- | --- | --- | --- | --- | --- | --- | --- | --- | --- | --- | --- | --- | --- | --- |
| 1 |  |  |  |  |  |  |  |  |  |  |  | 2 |  |  |  |  |  |  |  |  |  |  |  |
| 2 |  |  |  |  |  |  |  |  |  |  |  | 1 | 1 |  |  |  |  |  |  |  |  |  |  |
| 3 |  |  |  |  |  |  |  |  |  |  |  | 1 |  |  |  |  |  |  |  |  |  |  |  |
| 4 |  |  |  |  |  |  |  |  |  |  | 4 | 1 |  |  |  |  |  |  |  |  |  |  |  |
| 5 | 25 | 15 | 16 | 13 | 6 | 8 | 12 | 2 | 12 | 5 |  |  |  |  |  |  |  |  |  |  |  |  |  |
| 6 |  |  | 1 |  |  |  |  |  |  |  |  |  |  |  |  |  |  |  |  |  |  |  |  |
| 7 |  |  |  |  |  | 1 |  |  |  |  |  |  |  |  |  |  |  |  |  |  |  |  |  |
| 8 |  |  |  |  |  |  | 1 |  |  |  |  |  |  |  |  |  |  |  |  |  |  |  |  |
| 9 | 1 |  |  |  |  |  |  |  |  |  |  |  |  |  |  |  |  |  |  |  |  |  |  |
| 10 | 1 |  |  |  |  |  |  |  |  |  |  |  |  |  |  |  |  |  |  |  |  |  |  |
| 11 |  |  |  |  | 1 |  |  |  |  |  |  |  |  |  |  |  |  |  |  |  |  |  |  |
| 12 |  |  |  |  |  |  | 1 |  |  |  |  |  |  |  |  |  |  |  |  |  |  |  |  |
| 13 | 1 |  |  |  |  |  |  |  |  |  |  |  |  |  |  |  |  |  |  |  |  |  |  |
| 14 |  |  | 1 |  |  |  |  |  |  |  |  |  |  |  |  |  |  |  |  |  |  |  |  |
| 15 |  |  |  |  |  |  | 1 |  |  |  |  |  |  |  |  |  |  |  |  |  |  |  |  |
| 16 |  |  |  |  |  |  |  |  | 1 |  |  |  |  |  |  |  |  |  |  |  |  |  |  |
| 17 |  |  |  |  |  | 1 |  |  |  |  |  |  |  |  |  |  |  |  |  |  |  |  |  |
| 18 |  | 1 |  |  |  |  |  |  |  |  |  |  |  |  |  |  |  |  |  |  |  |  |  |
| 19 | 1 | 1 |  | 1 |  |  |  |  |  |  |  |  |  |  |  |  |  |  |  |  |  |  |  |
| 20 |  |  |  |  |  | 1 |  |  |  |  |  |  |  |  |  |  |  |  |  |  |  |  |  |
| 21 | 1 |  |  |  |  |  | 1 |  |  |  |  |  |  |  |  |  |  |  |  |  |  |  |  |
| 22 |  | 1 |  |  |  |  |  |  | 1 |  |  |  |  |  |  |  |  |  |  |  |  |  |  |
| 23 |  |  | 1 |  |  |  |  |  |  |  |  |  |  |  |  |  |  |  |  |  |  |  |  |
| 24 |  | 1 |  |  |  |  |  |  |  |  |  |  |  |  |  |  |  |  |  |  |  |  |  |
| 25 |  |  |  |  |  |  |  |  | 1 |  |  |  |  |  |  |  |  |  |  |  |  |  |  |
| 26 |  |  | 1 |  |  |  |  |  | 1 |  |  |  |  |  |  |  |  |  |  |  |  |  |  |
| 27 | 3 |  | 1 |  |  | 1 |  | 1 |  | 2 |  |  |  |  |  |  |  |  |  |  |  |  |  |
| 28 |  |  |  |  |  |  |  |  | 1 |  |  |  |  |  |  |  |  |  |  |  |  |  |  |
| 29 | 1 |  |  |  |  |  |  |  |  |  |  |  |  |  |  |  |  |  |  |  |  |  |  |
| 30 |  |  |  |  |  |  |  |  |  | 1 |  |  |  |  |  |  |  |  |  |  |  |  |  |
| 31 |  |  |  |  |  | 1 |  |  |  |  |  |  |  |  |  |  |  |  |  |  |  |  |  |
| 32 |  |  |  |  |  |  |  | 1 |  |  |  |  |  |  |  |  |  |  |  |  |  |  |  |
| 33 |  |  |  |  |  | 1 |  |  |  |  |  |  |  |  |  |  |  |  |  |  |  |  |  |
| 34 |  |  |  |  |  | 1 |  |  |  |  |  |  |  |  |  |  |  |  |  |  |  |  |  |
| 35 |  |  |  |  |  |  |  |  |  |  |  |  |  | 1 |  |  |  |  |  |  |  |  |  |
| 36 |  |  |  |  |  |  |  |  |  |  |  |  |  | 1 |  |  |  |  |  |  |  |  |  |
| 37 |  |  |  |  |  |  |  |  |  |  |  |  |  | 1 |  |  |  |  |  |  |  |  |  |
| 38 |  |  |  |  |  |  |  |  |  |  |  |  |  |  | 1 |  |  |  |  |  |  |  |  |
| 39 |  |  |  |  |  |  |  |  |  |  |  |  |  |  | 1 |  |  |  |  |  |  |  |  |
| 40 |  |  |  |  |  |  |  |  |  |  |  |  |  |  | 1 |  |  |  |  |  |  |  |  |
| 41 |  |  |  |  |  |  |  |  |  |  |  |  |  |  | 1 |  |  |  |  |  |  |  |  |
| 42 |  |  |  |  |  |  |  |  |  |  |  |  |  |  |  | 1 |  |  |  |  |  |  |  |
| 43 |  |  |  |  |  |  |  |  |  |  |  |  |  |  |  | 1 |  |  |  |  |  |  |  |
| 44 |  |  |  |  |  |  |  |  |  |  |  |  |  |  |  | 1 |  |  |  |  |  |  |  |
| 45 |  |  |  |  |  |  |  |  |  |  |  |  |  |  |  | 1 |  |  |  |  |  |  |  |
| 46 |  |  |  |  |  |  |  |  |  |  |  |  |  |  |  | 1 |  |  |  |  |  |  |  |
| 47 |  |  |  |  |  |  |  |  |  |  |  |  |  |  |  | 1 |  |  |  |  |  |  |  |
| 48 |  |  |  |  |  |  |  |  |  |  |  |  |  |  |  | 1 |  |  |  |  |  |  |  |
| 49 |  |  |  |  |  |  |  |  |  |  |  |  | 1 |  |  |  |  |  |  |  |  |  |  |
| 50 |  |  |  |  |  |  |  |  |  |  |  |  | 1 |  |  |  |  |  |  |  |  |  |  |
| 51 |  |  |  |  |  |  |  |  |  |  |  |  | 1 |  |  |  |  |  |  |  |  |  |  |
| 52 |  |  |  |  |  |  |  |  |  |  |  |  |  |  |  |  | 1 |  |  |  |  |  |  |
| 53 |  |  |  |  |  |  |  |  |  |  |  |  |  |  |  |  |  | 1 |  |  |  |  |  |
| 54 |  |  |  |  |  |  |  |  |  |  |  |  |  |  |  |  | 1 |  |  |  |  |  |  |
| 55 |  |  |  |  |  |  |  |  |  |  |  |  |  |  |  |  | 1 |  |  |  |  |  |  |
| 56 |  |  |  |  |  |  |  |  |  |  |  |  |  |  |  |  | 1 |  |  |  |  |  |  |
| 57 |  |  |  |  |  |  |  |  |  |  |  |  |  |  |  |  | 2 |  |  |  |  |  |  |
| 58 |  |  |  |  |  |  |  |  |  |  |  |  |  |  |  |  |  | 1 |  |  |  |  |  |
| 59 |  |  |  |  |  |  |  |  |  |  |  |  |  |  |  |  | 1 |  |  |  |  |  |  |
| 60 |  |  |  |  |  |  |  |  |  |  |  |  |  |  |  |  | 1 |  |  |  |  |  |  |
| 61 |  |  |  |  |  |  |  |  |  |  |  |  |  |  |  |  | 1 |  |  |  |  |  |  |
| 62 |  |  |  |  |  |  |  |  |  |  |  |  |  |  |  |  |  | 1 |  |  |  |  |  |
| 63 |  |  |  |  |  |  |  |  |  |  |  |  |  |  |  |  |  |  |  | 1 |  |  |  |
| 64 |  |  |  |  |  |  |  |  |  |  |  |  |  |  |  |  |  |  |  | 1 |  |  |  |
| 65 |  |  |  |  |  |  |  |  |  |  |  |  |  |  |  |  |  |  |  | 1 |  |  |  |
| 66 |  |  |  |  |  |  |  |  |  |  |  |  |  |  |  |  |  |  |  | 1 |  |  |  |
| 67 |  |  |  |  |  |  |  |  |  |  |  |  |  |  |  |  |  |  |  | 1 |  |  |  |
| 68 |  |  |  |  |  |  |  |  |  |  |  |  |  |  |  |  |  |  | 1 |  |  |  |  |
| 69 |  |  |  |  |  |  |  |  |  |  |  |  |  |  |  |  |  |  | 1 |  |  |  |  |
| 70 |  |  |  |  |  |  |  |  |  |  |  |  |  |  |  |  |  |  |  |  |  |  | 1 |
| 71 |  |  |  |  |  |  |  |  |  |  |  |  |  |  |  |  |  |  |  |  |  |  | 1 |
| 72 |  |  |  |  |  |  |  |  |  |  |  |  |  |  |  |  |  |  | 1 |  |  |  |  |
| 73 |  |  |  |  |  |  |  |  |  |  |  |  |  |  |  |  |  |  | 1 |  |  |  |  |
| 74 |  |  |  |  |  |  |  |  |  |  |  |  |  |  |  |  |  |  | 1 |  |  |  |  |
| 75 |  |  |  |  |  |  |  |  |  |  |  |  |  |  |  |  |  |  | 1 |  |  |  |  |
| 76 |  |  |  |  |  |  |  |  |  |  |  |  |  |  |  |  |  |  |  | 1 |  |  |  |
| 77 |  |  |  |  |  |  |  |  |  |  |  |  |  |  |  |  |  |  | 1 |  |  |  |  |
| 78 |  |  |  |  |  |  |  |  |  |  |  |  |  |  |  |  |  |  | 2 |  |  |  |  |
| 79 |  |  |  |  |  |  |  |  |  |  |  |  |  |  |  |  |  |  | 1 |  |  |  |  |
| 80 |  |  |  |  |  |  |  |  |  |  |  |  |  |  |  |  |  |  |  |  | 1 |  |  |
| 81 |  |  |  |  |  |  |  |  |  |  |  |  |  |  |  |  |  |  |  |  | 1 |  |  |
| 82 |  |  |  |  |  |  |  |  |  |  |  |  |  |  |  |  |  |  |  |  |  | 1 |  |
| 83 |  |  |  |  |  |  |  |  |  |  |  |  |  |  |  |  |  |  |  |  | 1 |  |  |
| 84 |  |  |  |  |  |  |  |  |  |  |  |  |  |  |  |  |  |  |  |  |  |  | 1 |
| 85 |  |  |  |  |  |  |  |  |  |  |  |  |  |  |  |  |  |  |  |  |  | 1 |  |
| 86 |  |  |  |  |  |  |  |  |  |  |  |  |  |  |  |  |  |  |  |  |  | 1 |  |
| 87 |  |  |  |  |  |  |  |  |  |  |  |  |  |  |  |  |  |  |  | 1 |  |  |  |
| 88 |  |  |  |  |  |  |  |  |  |  |  |  |  |  |  |  |  |  |  | 1 |  |  |  |

HAP1 ATCCGCGCGGAA-CTCAGCCAGCCCGGCGCACTACTTGGGGACGACCAGATTTATAACGTAATTGTCACTGCCCACGCCTTTGTAATGATTTTCTTTATAGTAATACCAATTATGATTGGGGGCTTTGGGAACTGGCTAATTCCCCTAATGATTGGGGCCCCCGACATGGCCTTCCCCCGAATGAACAACATGAGCTTCTGGCTTTTGCCCCCCTCATTCCTGCTCCTTCTCGCCTCTTCGGGCGTTGAGGCTGGGGCTGGGACAGGGTGGACTGTCTACCCCCCATTGGCAGGAAACCTGGCCCACGCAGGCGCATCTGTCGACCTAACAATCTTTTCTCTCCACCTCGCCGGGATCTCTTCCATTCTTGGCGCCATTAACTTTATCACCACAATCCTAAACATGAAGCCACCCGCTATCTCGCAGTATCAAACGCCCCTCTTCGTATGGGCCGTTCTTATTACAGCCGTTCTCCTCCTTCTCTCACTGCCCGTCCTCGCCGCCGGCATCACCATGCTACTTACAGACC

HAP2 ATCCGCGCGGAA-CTCAGCCAGCCCGGCGCACTACTTGGGGACGACCAGATTTATAACGTAATTGTCACTGCCCACGCCTTTGTAATGATTTTCTTTATAGTAATACCAATTATGATTGGGGGCTTTGGGAACTGACTAATTCCCCTAATGATTGGGGCCCCCGACATGGCCTTCCCCCGAATGAACAACATGAGCTTCTGGCTTTTGCCCCCCTCATTCCTGCTCCTTCTCGCCTCTTCGGGCGTTGAGGCTGGGGCTGGGACAGGGTGGACTGTCTACCCCCCATTGGCAGGAAACCTGGCCCACGCAGGCGCATCTGTCGACCTAACAATCTTTTCTCTCCACCTCGCCGGGATCTCTTCCATTCTTGGCGCCATTAACTTTATCACCACAATCCTAAACATGAAGCCACCCGCTATCTCGCAGTATCAAACGCCCCTCTTCGTATGGGCCGTTCTTATTACAGCCGTTCTCCTCCTTCTCTCACTGCCCGTCCTCGCCGCCGGCATCACCATGCTACTTACAGACC

HAP3 ATCCGCGCGGAA-CTCAGCCAGCCCGGCGCACTACTTGGGGACGACCAGATTTATAACGTAATTGTCACTGCCCACGCCTTTGTAATGATTTTCTTTATAGTAATACCAATTATGATTGGGGGCTTTGGGAACTGACTAATTCCCCTAATGATTGGGGCCCCCGACATGGCCTTCCCCCGAATGAACAACATGAGCTTCTGGCTTTTGCCCCCCTCATTCCTGCTCCTTCTCGCCTCTTCGGGCGTTGAGGCTGGGGCTGGGACAGGGTGGACTGTCTACCCACCATTGGCAGGAAACCTGGCCCACGCAGGCGCATCTGTCGACCTAACAATCTTTTCTCTCCACCTCGCCGGGATCTCTTCCATTCTTGGCGCCATTAACTTTATCACCACAATCCTAAACATGAAGCCACCCGCTATCTCGCAGTATCAAACGCCCCTCTTCGTATGGGCCGTTCTTATTACAGCCGTTCTCCTCCTTCTCTCACTGCCCGTCCTCGCCGCCGGCATCACCATGCTACTTACAGACC

HAP4 ATCCGCGCGGAGGCTCAGCCAGCCCGGCG-ACTACTTGGGGACGACCAGATTTATAACGTAATTGTCACTGCCCACGCCTTTGTAATGATTTTCTTTATAGTAATACCAATTATGATTGGGGGCTTTGGGAACTGACTAATTCCCCTAATGATTGGGGCCCCCGACATGGCCTTCCCCCGAATGAACAACATGAGCTTCTGGCTTTTGCCCCCCTCATTCCTGCTCCTTCTCGCCTCTTCGGGCGTTGAGGCTGGGGCTGGGACAGGGTGGACTGTCTACCCACCATTGGCAGGAAACCTGGCCCACGCAGGCGCATCTGTCGACCTAACAATCTTTTCTCTCCACCTCGCCGGGATCTCTTCCATTCTTGGCGCCATTAACTTTATCACCACAATCCTAAACATGAAGCCACCCGCTATCTCGCAGTATCAAACGCCCCTCTTCGTATGGGCCGTTCTTATTACAGCCGTTCTCCTCCTTCTCTCACTGCCCGTCCTCGCCGCCGGCATCACCATGCTACTTACAGACC

HAP5 ATCCGCGCGGAGGCTCAGCCAGCCCGGCG-ACTACTTGGGGACGACCAGATTTATAACGTAATTGTCACTGCCCACGCCTTTGTAATGATTTTCTTTATAGTAATACCAATTATGATTGGGGGCTTTGGGAACTGACTAATTCCCCTAATGATTGGGGCCCCCGACATGGCCTTCCCCCGAATGAACAACATGAGCTTCTGGCTTTTGCCCCCCTCATTCCTGCTCCTTCTCGCCTCTTCGGGCGTTGAGGCTGGGGCTGGGACAGGGTGGACTGTCTACCCACCATTGGCAGGAAACCTGGCCCACGCAGGCGCATCTGTCGACCTAACAATCTTTTCTCTCCACCTCGCCGGGATCTCTTCCATTCTTGGCGCCATTAACTTTATCACCACAATCCTAAACATGAAGCCACCCGCTATCTCGCAGTATCAAACGCCCCTCTTCGTATGGGCCGTTCTTATCACAGCCGTTCTCCTCCTTCTCTCACTGCCCGTCCTCGCCGCCGGCATCACCATGCTACTTACAGACC

HAP6 ATCCGCGCGGAGGCTCAGCCAGCCCGGCG-ACTACTTGGGGACGACCAGATTTATAACGTAATTGTCACTGCCCACGCCTTTGTAATGATTTTCTTTATAGTAATACCAATTATGATTGGGGGCTTTGGAAACTGACTAATTCCCCTAATGATTGGGGCCCCCGACATGGCCTTCCCCCGAATGAACAACATGAGCTTCTGGCTTTTGCCCCCCTCATTCCTGCTCCTTCTCGCCTCTTCGGGCGTTGAGGCTGGGGCTGGGACAGGGTGGACTGTCTACCCACCATTGGCAGGAAACCTGGCCCACGCAGGCGCATCTGTCGACCTAACAATCTTTTCTCTCCACCTCGCCGGGATCTCTTCCATTCTTGGCGCCATTAACTTTATCACCACAATCCTAAACATGAAGCCACCCGCTATCTCGCAGTATCAAACGCCCCTCTTCGTATGGGCCGTTCTTATCACAGCCGTTCTCCTCCTTCTCTCACTGCCCGTCCTCGCCGCCGGCATCACCATGCTACTTACAGACC

HAP7 ATCCGCGCGGAGGCTCAGCCAGCCCGGCG-ACTACTTGGGGACGACCAGATTTATAACGTAATTGTCACTGCCCACGCCTTTGTAATGATTTTCTTTATAGTAATACCAATTATGATTGGGGGCTTTGGGAACTGACTAATTCCCCTAATGATTGGAGCCCCCGACATGGCCTTCCCCCGAATGAACAACATGAGCTTCTGGCTTTTGCCCCCCTCATTCCTGCTCCTTCTCGCCTCTTCGGGCGTTGAGGCTGGGGCTGGGACAGGGTGGACTGTCTACCCACCATTGGCAGGAAACCTGGCCCACGCAGGCGCATCTGTCGACCTAACAATCTTTTCTCTCCACCTCGCCGGGATCTCTTCCATTCTTGGCGCCATTAACTTTATCACCACAATCCTAAACATGAAGCCACCCGCTATCTCGCAGTATCAAACGCCCCTCTTCGTATGGGCCGTTCTTATCACAGCCGTTCTCCTCCTTCTCTCACTGCCCGTCCTCGCCGCCGGCATCACCATGCTACTTACAGACC

HAP8 ATCCGCGCGGAGGCTCAGCCAGCCCGGCG-ACTACTTGGGGACGACCAGATTTATAACGTAATTGTCACTGCCCACGCCTTTGTAATGATTTTCTTTATAGTAATACCAATTATGATTGGGGGCTTTGGGAACTGACTAATTCCCCTAATGATTGGGGCCCCCGACATGGCCTTCCCCCGAATGAACAACATGAGCTTCTGGCTTTTGCCCCCCTCATTCCTGCTCCTTCTCGCCTCTTCGGGCGTTGAGGCTGGGGCTGGGACAGGGTGGACTGTCTACCCACCATTGGCAGGAAACCTGGCCCACGCAGGCGCATCTGTCGACCTAACAATCTTTTCTCTCCACCTCGCCGGGATCTCTTCCATTCTTGGCGCCATTAACTTTATCACCACAATCCTAAACATGAAGCCACCCGCTATCTCGCAGTATCAAACGCCCCTCTTCGTATGGGCCGTTCTTATCACAGCCGTTCTCCTCCTTCTCTCACTGCCCGTCCTCGCCGCCGGCATCACCATGCTACTTAC-GACC

HAP9 ATCCGCGCGGAGGCTCAGCCAGCCCGGCG-ACTACTTGGGGACGACCAGATTTATAACGTAATTGTCACTGCCCACGCCTTTGTAATGATTTTCTTTATAGTAATACCAATTATGATTGGGGGCTTTGGGAACTGACTAATTCCCCTAATGATTGGGGCCCCCGACATGGCCTTCCCCCGAATGAACAACATGAGCTTCTGGCTTTTGCCCCCCTCATTCCTGCTCCTTCTCGCCTCTTCGGGCGTTGAGGCTGGGGCTGGGACAGGGTGGACTGTCTACCCACCATTGGCAGGAAACCTGGCCCACGCAGGCGCATCTGTCGACCTAACAATCTTTTCCCTCCACCTCGCCGGGATCTCTTCCATTCTTGGCGCCATTAACTTTATCACCACAATCCTAAACATGAAGCCACCCGCTATCTCGCAGTATCAAACGCCCCTCTTCGTATGGGCCGTTCTTATCACAGCCGTTCTCCTCCTTCTCTCACTGCCCGTCCTCGCCGCCGGCATCACCATGCTACTTACAGACC

HAP10 ATCCGCGCGGAGGCTCAGCCAGCCCGGCG-ACTACTTGGGGACGACCAGATTTATAACGTAATTGTCACTGCCCACGCCTTTGTAATGATTTTCTTTATAGTAATACCAATTATGATTGGGGGCTTTGGGAACTGACTAATTCCCCTAATGATTGGGGCCCCCGACATGGCCTTCCCCCGAATGAACAACATGAGCTTCTGGCTTTTGCCCCCCTCATTCTTGCTCCTTCTCGCCTCTTCGGGCGTTGAGGCTGGGGCTGGGACAGGGTGGACTGTCTACCCACCATTGGCAGGAAACCTGGCCCACGCAGGCGCATCTGTCGACCTAACAATCTTTTCTCTCCACCTCGCCGGGATCTCTTCCATTCTTGGCGCCATTAACTTTATCACCACAATCCTAAACATGAAGCCACCCGCTATCTCGCAGTATCAAACGCCCCTCTTCGTATGGGCCGTTCTTATCACAGCCGTTCTCCTCCTTCTCTCACTGGCCGTCCTCGCCGCCGGCATCACCATGCTACTTACAGACC

HAP11 ATCCGCGCGGAGGCTCAGCCAGCCCGGCG-ACTACTTGGGGACGACCAGATTTATAACGTAATTGTCACTGCCCACGCCTTTGTAATGATTTTCTTTATAGTAATACCAATTATGATTGGGGGCTTTGGGAACTGACTAATTCCCCTAATGATTGGGGCCCCCGACATGGCCTTCCCCCGAATGAACAACATGAGCTTCTGGCTTTTGCCCCCCTCATTCCTGCTCCTTCTCGCCTCTTCGGGCGTTGAGGCTGGGGCTGGGACAGGGTGGACTGTCTACCCACCATTGGCAGGAAACCTGGCCCACGCTGGCGCATCTGTCGACCTAACAATCTTTTCTCTCCACCTCGCCGGGATCTCTTCCATTCTTGGCGCCATTAACTTTATCACCACAATCCTAAACATGAAGCCACCCGCTATCTCGCAGTATCAAACGCCCCTCTTCGTATGGGCCGTTCTTATCACAGCCGTTCTCCTCCTTCTCTCACTGCCCGTCCTCGCCGCCGGCATCACCATGCTACTTACAGACC

HAP12 ATCCGCGCGGAGGCTCAGCCAGCCCGGCG-ACTACTTGGGGACGACCAGATTTATAACGTAATTGTCACTGCCCACGCCTTTGTAATGATTTTCTTTATAGTAATACCAATTATGATTGGGGGCTTTGGGAACTGACTAATTCCCCTAATGATTGGGGCCCCCGACATGGCCTTCCCCCGAATGAACAACATGAGCTTCTGGCTTTTGCCCCCCTCATTCCTGCTCCTTCTCGCCTCTTCGGGCGTTGAGGCTGGGGCTGGGACAGGGTGGACTGTCTACCCACCATTGGCAGGAAACCTGGCCCACGCAGGCGCATCTGTCGACCTAACAATCTTTTCTCTCCACCTCGCCGGGATCTCTTCCATTCTTGGCGCCATTAACTTTATCACCACAATCCTAAACATGAAGCCACCCGCTATCTCGCAGTATCAAACACCCCTCTTCGTATGGGCCGTTCTTATCACAGCCGTTCTCCTCCTTCTCTCACTGCCCGTCCTCGCCGCCGGCATCACCATGCTACTTACAGACC

HAP13 ATCCGCGCGGAGGCTCAGCCAGCCCGGCG-ACTACTTGGGGACGACCAGATTTATAACGTAATTGTCACTGCCCACGCCTTTGTAATGATTTTCTTTATAGTAATACCAATTATGATTGGGGGCTTTGGGAACTGACTAATTCCCCTAATGATCGGGGCCCCCGACATGGCCTTCCCCCGAATGAACAACATGAGCTTCTGGCTTTTGCCCCCCTCATTCCTGCTCCTTCTCGCCTCTTCGGGCGTTGAGGCTGGGGCTGGGACAGGGTGGACTGTCTACCCACCATTGGCAGGAAACCTGGCCCACGCAGGCGCATCTGTCGACCTAACAATCTTTTCTCTCCACCTCGCCGGGATCTCTTCCATTCTTGGCGCCATTAACTTTATCACCACAATCCTAAACATGAAGCCACCCGCTATCTCGCAGTATCAAACGCCCCTCTTCGTATGGGCCGTTCTTATCACAGCCGTTCTCCTCCTTCTCTCACTGCCCGTCCTCGCCGCCGGCATCACCATGCTACTTACAGACC

HAP14 ATCCGCGCGGAGGCTCAGCCAGCCCGGCG-ACTACTTGGGGACGACCAGATTTATAACGTAATTGTCACTGCCCACGCCTTTGTAATGATTTTCTTTATAGTAATACCAATTATGATTGGGGGCTTTGGGAACTGACTAATTCCCCTAATGATTGGGGCCCCCGACATGGCCTTCCCCCGAATGAACAACATGAGTTTCTGGCTTTTGCCCCCCTCATTCCTGCTCCTTCTCGCCTCTTCGGGCGTTGAGGCTGGGGCTGGGACAGGGTGGACTGTCTACCCACCATTGGCAGGAAACCTGGCCCACGCAGGCGCATCTGTCGACCTAACAATCTTTTCTCTCCACCTCGCCGGGATCTCTTCCATTCTTGGCGCCATTAACTTTATCACCACAATCCTAAACATGAAGCCACCCGCTATCTCGCAGTATCAAACGCCCCTCTTCGTATGGGCCGTTCTTATCACAGCCGTTCTCCTCCTTCTCTCACTGCCCGTCCTCGCCGCCGGCATCACCATGCTACTTACAGACC

HAP15 ATCCGCGCGGAGGCTCAGCCAGCCCGGCG-ACTACTTGGGGACGACCAGATTTATAACGTAATTGTCACTGCCCACGCCTTTGTAATGATTTTCTTTATAGTAATACCAATTATGATTGGGGGCTTTGGGAACTGACTAATTCCCCTAATGATTGGGGCCCCCGACATAGCCTTCCCCCGAATGAACAACATGAGCTTCTGGCTTTTGCCCCCCTCATTCCTGCTCCTTCTCGCCTCTTCGGGCGTTGAGGCTGGGGCTGGGACAGGGTGGACTGTCTACCCACCATTGGCAGGAAACCTGGCCCACGCAGGCGCATCTGTCGACCTAACAATCTTTTCTCTCCACCTCGCCGGGATCTCTTCCATTCTTGGCGCCATTAACTTTATCACCACAATCCTAAACATGAAGCCACCCGCTATCTCGCAGTATCAAACGCCCCTCTTCGTATGGGCCGTTCTTATCACAGCCGTTCTCCTCCTTCTCTCACTGCCCGTCCTCGCCGCCGGCATCACCATGCTACTTACAGACC

HAP16 ATCCGCGCGGAGGCTCAGCCAGCCCGGCG-ACTACTTGGGGACGACCAGATTTATAACGTAATTGTCACTGCCCACGCCTTTGTAATGATTTTCTTTATAGTAATACCAATTATGATTGGGGGCTTTGGGAACTGACTAATTCCCCTAATGATTGGGGCCCCCGACATGGCCTTCCCCCGAATGAACAACATGAGCTTCTGGCTTTTGCCCCCCTCATTCCTGCTTCTTCTCGCCTCTTCGGGCGTTGAGGCTGGGGCTGGGACAGGGTGGACTGTCTACCCACCATTGGCAGGAAACCTGGCCCACGCAGGCGCATCTGTCGACCTAACAATCTTTTCTCTCCACCTCGCCGGGATCTCTTCCATTCTTGGCGCCATTAACTTTATCACCACAATCCTAAACATGAAGCCACCCGCTATCTCGCAGTATCAAACGCCCCTCTTCGTATGGGCCGTTCTTATCACAGCCGTTCTCCTCCTTCTCTCACTGCCCGTCCTCGCCGCCGGCATCACCATGCTACTTACAGACC

HAP17 ATCCGCGCGGAGGCTCAGCCAGCCCGGCG-ACTACTTGGGGACGACCAGATTTATAACGTAATTGTCACTGCCCACGCCTTTGTAATGATTTTCTTTATAGTAATACCAATTATGATTGGGGGCTTTGGGAACTGACTAATTCCCCTAATGATTGGGGCCCCCGACATGGCCTTCCCCCGAATGAACAACATGAGCTTCTGGCTTTTGCCCCCCTCATTCCTGCTCCTTCTCGCCTCTTCGGGCGTTGAGGCTGGGGCTGGGACAGGGTGGACTGTCTACCCACCATTGGCAGGAAACCTGGCCCACGCAGGCGCATCTGTCGACCTAACAATCTTTTCTCTCCACCTCGCCGGGATCTCTTCCATTCTTGGCGCCATTAACTTTATCACCACAATCCTAAACATGAAGCCACCCGCTATCTCGCAGTATCAAACGCCCCTCTTCGTATGGGCCGTTCTTATCACAGCCGTTCTCCTCCTTCTCTCACTGCCCGTCCTCGCCGCCGGCATCACCATGCTACTCACAGACC

HAP18 ATCCGCGCGGAGGCTCAGCCAGCCCGGCG-ACTACTTGGGGACGACCAGATTTATAACGTAATTGTCACTGCCCACGCCTTTGTAATGATTTTCTTTATAGTAATACCAATTATGATTGGTGGCTTTGGGAACTGACTAATTCCCCTAATGATTGGGGCCCCCGACATGGCCTTCCCCCGAATGAACAACATGAGCTTCTGGCTTTTGCCCCCCTCATTCCTGCTCCTTCTCGCCTCTTCGGGCGTTGAGGCTGGGGCTGGGACAGGGTGGACTGTCTACCCACCATTGGCAGGAAACCTGGCCCACGCAGGCGCATCTGTCGACCTAACAATCTTTTCTCTCCACCTCGCCGGGATCTCTTCCATTCTTGGCGCCATTAACTTTATCACCACAATCCTAAACATGAAGCCACCCGCTATCTCGCAGTATCAAACGCCCCTCTTCGTATGGGCCGTTCTTATCACAGCCGTTCTCCTCCTTCTCTCACTGCCCGTCCTCGCCGCCGGCATCACCATGCTACTTACAGACC

HAP19 ATCCGCGCGGAGGCTCAGCCAGCCCGGCG-ACTACTTGGGGACGACCAGATTTATAACGTAATTGTCACTGCCCACGCCTTTGTAATGATTTTCTTTATAGTAATACCAATTATGATTGGGGGCTTTGGGAACTGACTAATTCCCCTAATGATTGGGGCCCCCGACATGGCCTTCCCCCGAATGAACAACATGAGCTTCTGGCTTTTACCCCCCTCATTCCTGCTCCTTCTCGCCTCTTCGGGCGTTGAGGCTGGGGCTGGGACAGGGTGGACTGTCTACCCACCATTGGCAGGAAACCTGGCCCACGCAGGCGCATCTGTCGACCTAACAATCTTTTCTCTCCACCTCGCCGGGATCTCTTCCATTCTTGGCGCCATTAACTTTATCACCACAATCCTAAACATGAAGCCACCCGCTATCTCGCAGTATCAAACGCCCCTCTTCGTATGGGCCGTTCTTATCACAGCCGTTCTCCTCCTTCTCTCACTGCCCGTCCTCGCCGCCGGCATCACCATGCTACTTACAGACC

HAP20 ATCCGCGCGGAGGCTCAGCCAGCCCGGCG-ACTACTTGGGGACGACCAGATTTATAACGTAATTGTCACTGCCCACGCCTTTGTAATGATTTTCTTTATAGTAATACCAATTATGATTGGGGGCTTTGGGAACTGACTAATTCCCCTAATGATTGGGGCCCCCGACATGGCCTTCCCCCGAATGAACAACATGAGCTTCTGGCTTTTGCCCCCCTCATTCCTGCTCCTTCTCGCCTCTTCGGGCGTTGAGGCTGGGGCTGGGACAGGGTGGACTGTCTACCCACCATTGGCAGGAAACCTGGCCCACGCAGGCGCATCTGTCGACCTAACAATCTTTTCTCTCCACCTCGCCGGGATTTCTTCCATTCTTGGCGCCATTAACTTTATCACCACAATCCTAAACATGAAGCCACCCGCTATCTCGCAGTATCAAACGCCCCTCTTCGTATGGGCCGTTCTTATCACAGCCGTTCTCCTCCTTCTCTCACTGCCCGTCCTCGCCGCCGGCATCACCATGCTACTTACAGACC

HAP21 ATCCGCGCGGAGGCTCAGCCAGCCCGGCG-ACTACTTGGGGACGACCAGATTTATAACGTAATTGTCACTGCCCACGCCTTTGTAATGATTTTCTTTATAGTAATACCAATTATGATTGGGGGCTTTGGGAACTGACTAATTCCCCTAATGATTGGGGCCCCCGACATGGCCTTCCCCCGAATGAACAACATGAGCTTCTGGCTTTTGCCCCCCTCATTCCTGCTCCTTCTCGCCTCTTCGGGCGTTGAGGCTGGGGCTGGGACAGGGTGGACTGTCTACCCACCATTGGCAGGAAACCTAGCCCACGCAGGCGCATCTGTCGACCTAACAATCTTTTCTCTCCACCTCGCCGGGATCTCTTCCATTCTTGGCGCCATTAACTTTATCACCACAATCCTAAACATGAAGCCACCCGCTATCTCGCAGTATCAAACGCCCCTCTTCGTATGGGCCGTTCTTATCACAGCCGTTCTCCTCCTTCTCTCACTGCCCGTCCTCGCCGCCGGCATCACCATGCTACTTACAGACC

HAP22 ATCCGCGCGGAGGCTCAGCCAGCCCGGCG-ACTACTTGGGGATGACCAGATTTATAACGTAATTGTCACTGCCCACGCCTTTGTAATGATTTTCTTTATAGTAATACCAATTATGATTGGGGGCTTTGGGAACTGACTAATTCCCCTAATGATTGGGGCCCCCGACATGGCCTTCCCCCGAATGAACAACATGAGCTTCTGGCTTTTGCCCCCCTCATTCCTGCTCCTTCTCGCCTCTTCGGGCGTTGAGGCTGGGGCTGGGACAGGGTGGACTGTCTACCCACCATTGGCAGGAAACCTGGCCCACGCAGGCGCATCTGTCGACCTAACAATCTTTTCTCTCCACCTCGCCGGGATCTCTTCCATTCTTGGCGCCATTAACTTTATCACCACAATCCTAAACATGAAGCCACCCGCTATCTCGCAGTATCAAACGCCCCTCTTCGTATGGGCCGTTCTTATCACAGCCGTTCTCCTCCTTCTCTCACTGCCCGTCCTCGCCGCCGGCATCACCATGCTACTTACAGACC

HAP23 ATCCGCGCGGAGGCTCAGCCAGCCCGGCG-ACTACTTGGGGACGACCAGATTTATAACGTAATTGTCACTGCCCACGCCTTTGTAATGATTTTCTTTATAGTAATACCAATTATGATTGGGGGCTTTGGGAACTGACTAATTCCCCTAATGATTGGGGCCCCCGACATGGCCTTCCCCCGAATGAACAACATGAGCTTCTGGCTTTTGCCCCCCTCATTCCTGCTCCTTCTCGCCTCTTCGGGCGTTGAGGCTGGGGCTGGGACAGGGTGGACTGTCTACCCACCATTGGCAGGAAACCTGGCCCACGCAGGCGCATCTGTCGACCTAACAATCTTTTCTCTCCACCTCGCCGGGATCTCTTCCATTCTTGGCGCCATTAACTTTATCACCACAATCCTAAACATGAAGCCGCCCGCTATCTCGCAGTATCAAACGCCCCTCTTCGTATGGGCCGTTCTTATCACAGCCGTTCTCCTCCTTCTCTCACTGCCCGTCCTCGCCGCCGGCATCACCATGCTACTTACAGACC

HAP24 ATCCGCGCGGAGGCTCAGCCAGCCCGGCG-ACTACTTGGGGACGACCAGATTTATAACGTAATTGTCACTGCCCACGCCTTTGTAATGATTTTCTTTATAGTAATACCAATTATGATTGGGGGCTTTGGGAACTGACTAATTCCCCTAATGATTGGGGCCCCCGACATGGCCTTCCCCCGAATGAACAACATGAGCTTCTGGCTTTTGCCCCCCTCATTCCTGCTCCTTCTCGCCTCTTCGGGCGTTGAGGCTGGGGCTGGGACAGGGTGGACTGTCTACCCACCATTGGCAGGAAACCTGGCCCACGCAGGCGCATCTGTCGACCTAACAATCTTTTCTCTCCACCTCGCCGGGATCTCTTCCATTCTTGGCGCCATTAACTTTATCACCACAATCCTAAACATGAAGCCACCCGCTATCTCGCAGTATCAAACGCCCCTCTTCGTATGGGCCGTTCTTATCACAGCCGTCCTCCTCCTTCTCTCACTGCCCGTCCTCGCCGCCGGCATCACCATGCTACTTACAGACC

HAP25 ATCCGCGCGGAGGCTCAGCCAGCCCGGCG-ACTACTTGGGGACGACCAGATTTATAACGTAATTGTCACTGCCCACGCCTTTGTAATGATTTTCTTTATAGTAATACCAATTATGATTGGGGGCTTTGGGAACTGACTAATTCCCCTAATGATTGGGGCCCCCGACATGGCCTTCCCCCGAATGAACAACATGAGCTTCTGGCTTTTGCCCCCCTCATTCCTGCTCCTTCTCGCCTCTTCGGGCGTTGAAGCTGGGGCTGGGACAGGGTGGACTGTCTACCCACCATTGGCAGGAAACCTGGCCCACGCAGGCGCATCTGTCGACCTAACAATCTTTTCTCTCCACCTCGCCGGGATCTCTTCCATTCTTGGCGCCATTAACTTTATCACCACAATCCTAAACATGAAGCCACCCGCTATCTCGCAGTATCAAACGCCCCTCTTCGTATGGGCCGTTCTTATCACAGCCGTTCTCCTCCTTCTCTCACTGCCCGTCCTCGCCGCTGGCATCACCATGCTACTTACAGACC

HAP26 ATCCGCGCGGAGGCTCAGCCAGCCCGGCG-ACTACTTGGGGACGACCAGATTTATAACGTAATTGTCACTGCCCACGCCTTTGTAATGATTTTCTTTATAGTAATACCAATTATGATTGGGGGCTTTGGGAACTGACTAATTCCCCTAATGATTGGGGCCCCCGACATGGCCTTCCCCCGAATGAACAACATGAGCTTCTGGCTTTTGCCCCCCTCATTCCTGCTCCTTCTCGCCTCTTCGGGCGTTGAAGCTGGGGCTGGGACAGGGTGGACTGTCTACCCACCATTGGCAGGAAACCTGGCCCACGCAGGCGCATCTGTCGACCTAACAATCTTTTCTCTCCACCTCGCCGGGATCTCTTCCATTCTTGGCGCCATTAACTTTATCACCACAATCCTAAACATGAAGCCACCCGCTATCTCGCAGTATCAAACGCCCCTCTTCGTATGGGCCGTTCTTATCACAGCCGTTCTCCTCCTTCTCTCACTGCCCGTCCTCGCCGCCGGCATCACCATGCTACTTACAGACC

HAP27 ATCCGCGCGGAGGCTCAGCCAGCCCGGCG-ACTACTTGGGGACGACCAGATTTATAACGTAATTGTCACTGCCCACGCCTTTGTAATGATTTTCTTTATAGTAATACCAATTATGATTGGGGGCTTTGGGAACTGACTAATTCCCCTAATGATTGGGGCCCCCGACATGGCCTTCCCCCGAATGAACAACATGAGCTTCTGGCTTTTGCCCCCCTCATTCCTGCTCCTTCTCGCCTCTTCGGGCGTTGAGGCTGGGGCTGGGACAGGGTGGACTGTCTACCCACCATTGGCAGGAAACCTGGCCCACGCAGGCGCATCTGTCGACCTAACAATCTTTTCTCTCCACCTCGCCGGGATCTCTTCCATTCTTGGCGCCATTAACTTTATCACCACAATCCTAAACATGAAGCCACCCGCTATCTCGCAGTATCAAACGCCCCTCTTCGTATGGGCCGTTCTTATCACAGCCGTTCTCCTCCTTCCCTCACTGCCCGTCCTCGCCGCCGGCATCACCATGCTACTTACAGACC

HAP28 ATCCGCGCGGAGGCTCAGCCAGCCCGGCG-ACTACTTGGGGACGACCAGATTTATAACGTAATTGTCACTGCCCACGCCTTTGTAATGATTTTCTTTATAGTAATACCAATTATGATTGGGGGCTTTGGGAACTGACTAATTCCCCTAATGATTGGGGCCCCCGACATGGCCTTCCCCCGAATGAACAACATGAGCTTCTGGCTTTTGCCCCCCTCATTCCTGCTCCTTCTCGCCTCTTCGGGCGTTGAGGCTGGGGCTGGGACAGGGTGGACTGTCTACCCACCATTGGCAGGAAACCTGGCCCACGCAGGCGCATCCGTCGACCTAACAATCTTTTCTCTCCACCTCGCCGGGATCTCTTCCATTCTTGGCGCCATTAACTTTATCACCACAATCCTAAACATGAAGCCACCCGCTATCTCGCAGTATCAAACGCCCCTCTTCGTATGGGCCGTTCTTATCACAGCCGTTCTCCTCCTTCCCTCACTGCCCGTCCTCGCCGCCGGCATCACCATGCTACTTACAGACC

HAP29 ATCCGCGCGGAGGCTCAGCCAGCCCGGCG-ACTACTTGGGGACGACCAGATTTATAACGTAATTGTCACTGCCCACGCCTTTGTAATGATTTTCTTTATAGTAATACCAATTATGATTGGGGGCTTTGGGAACTGACTAATTCCCCTAATGATTGGGGCCCCCGACATGGCCTTCCCCCGAATGAACAACATGAGCTTCTGGCTTTTGCCCCCCTCATTCCTGCTCCTTCTCGCCTCTTCGGGCGTTGAGGCTGGGGCTGGGACAGGGTGGACTGTCTACCCACCATTGGCAGGAAACCTGGCCCACGCAGGCGCATCTGTCGACCTAACAATCTTTTCTCTCCACCTCGCCGGGATCTCTTCCATTCTTGGCGCCATTAACTTTATCACCACAATCCTAAACATGAAACCACCCGCTATCTCGCAGTATCAAACGCCCCTCTTCGTATGGGCCGTTCTTATCACAGCCGTTCTCCTCCTTCCCTCACTGCCCGTCCTCGCCGCCGGCATCACCATGCTACTTACAGACC

HAP30 ATCCGCGCGGAGGCTCAGCCAGCCCGGCG-ACTACTTGGGGACGACCAGATTTATAACGTAATTGTCACTGCCCACGCCTTTGTAATGATTTTCTTTATAGTAATACCAATTATGATTGGGGGCTTTGGGAACTGACTAATTCCCCTAATGATTGGGGCCCCCGACATGGCCTTCCCCCGAATGAACAACATGAGCTTCTGGCTTTTGCCCCCCTCATTCCTGCTCCTTCTCGCCTCTTCGGGCGTTGAGGCTGGGGCCGGGACAGGGTGGACTGTCTACCCACCATTGGCAGGAAACCTGGCCCACGCAGGCGCATCTGTCGACCTAACAATCTTTTCTCTCCACCTCGCCGGGATCTCTTCCATTCTTGGCGCCATTAACTTTATCACCACAATCCTAAACATGAAGCCACCCGCTATCTCGCAGTATCAAACGCCCCTCTTCGTATGGGCCGTTCTTATCACAGCCGTTCTCCTCCTTCCCTCACTGCCCGTCCTCGCCGCCGGCATCACCATGCTACTTACAGACC

HAP31 ATCCGCGCGGAGGCTCAGCCAGCCCGGCG-ACTACTTGGGGACGACCAGATTTATAACGTAATTGTCACTGCCCACGCCTTTGTAATGATTTTCTTTATAGTAATACCAATTATGATTGGGGGTTTTGGGAACTGACTAATTCCCCTAATGATTGGGGCCCCCGACATGGCCTTCCCCCGAATGAACAACATGAGCTTCTGACTTTTGCCCCCCTCATTCCTGCTCCTTCTCGCCTCTTCGGGCGTTGAGGCTGGGGCTGGGACAGGGTGGACTGTCTACCCACCATTGGCAGGAAACCTGGCCCACGCAGGCGCATCTGTCGACCTAACAATCTTTTCTCTCCACCTCGCCGGGATCTCTTCCATTCTTGGCGCCATTAACTTTATCACCACAATCCTAAACATGAAGCCACCCGCTATCTCGCAGTATCAAACGCCCCTCTTCGTATGGGCCGTTCTTATCACAGCCGTTCTCCTCCTTCCCTCACTGCCCGTCCTCGCCGCCGGCATCACCATGCTACTTACAGACC

HAP32 ATCCGCGCGGAGGCTCAGCCAGCCCGGCG-ACTACTTGGGGACGACCAGATTTATAACGTAATTGTCACTGCCCACGCCTTTGTAATGATTTTCTTTATAGTAATACCAATTATGATTGGGGGCTTTGGGAACTGACTAATTCCCCTAATGATTGGGGCCCCCGACATGGCCTTCCCCCGAATGAACAACATGAGCTTCTGACTTTTGCCCCCCTCATTCCTGCTCCTTCTCGCCTCTTCGGGCGTTGAGGCTGGGGCTGGGACAGGGTGGACTGTCTACCCACCATTGGCAGGAAACCTGGCCCACGCAGGCGCATCTGTCGACCTAACAATCTTTTCTCTCCACCTCGCCGGGATCTCTTCCATTCTTGGCGCCATTAACTTTATCACCACAATCCTAAACATGAAGCCACCCGCTATCTCGCAGTATCAAACGCCCCTCTTCGTATGGGCCGTTCTTATCACAGCCGTTCTCCTCCTTCTCTCACTGCCCGTCCTCGCCGCCGGCATCACCATGCTACTTACAGACC

HAP33 ATCCGCGCGGAGGCTCAGCCAGCCCGGCG-ACTACTTGGGGACGACCAGATTTATAACGTAATTGTCACTGCCCACGCCTTTGTAATGATTTTCTTTATAGTAATACCAATTATGATTGGGGGCTTTGGGAACTGACTAATTCCCCTAATGATTGGGGCCCCCGACATGGCCTTCCCCCGAATGAACAACATGAGCTTCTGGCTTTTGCCCCCCTCATTCCTGCTCCTTCTCGCCTCTTCGGGCGTTGAGGCTGGGGCTGGGACAGGGTGGACTGTCTACCCCCCATTGGCAGGAAACCTGGCCCACGCAGGCGCATCTGTCGACCTAACAATCTTTTCTCTCCACCTCGCCGGGATCTCTTCCATTCTTGGCGCCATTAACTTTATCACCACAATCCTAAACATGAAGCCACCCGCTATCTCGCAGTATCAAACGCCCCTCTTCGTATGGGCCGTTCTTATCACAGCCGTTCTCCTCCTTCCCTCACTGCCCGTCCTCGCCGCCGGCATCACCATGCTACTTACAGACC

HAP34 ATCCGCGCGGAGGCTCAGCCAGCCCGGCG-ACTACTTGGGGACGACCAGATTTATAACGTAATTGTCACTGCCCACGCCTTTGTAATGATTTTCTTTATAGTAATACCAATTATGATTGGGGGCTTTGGGAACTGACTAATTCCCCTAATGATTGGGGCCCCCGACATGGCCTTCCCCCGAATGAACAACATGAGCTTCTGGCTTTTGCCCCCCTCATTCCTGCTCCTTCTCGCCTCTTCGGGCGTTGAGGCTGGGGCTGGGACAGGGTGGACTGTCTACCCCCCATTGGCAGGAAACCTGGCCCACGCAGGCGCATCTGTCGACCTAACAATCTTTTCTCTCCACCTCGCCGGGATCTCTTCCATTCTTGGCGCCATTAACTTTATCACCACAATCCTAAACATGAAGCCACCCGCTATCTCGCAGTATCAAACGCCCCTCTTCGTATGGGCCGTTCTTATCACAGCCGTTCTCCTCCTTCTCTCACTGCCCGTCCTCGCCGCCGGCATCACCATGCTACTTACAGACC

HAP35 ATCCGCGCGGAG-CTCAGCCAGCCCGGCGCACTACTTGGGGACGACCAGATTTATAACGTAATTGTCACTGCCCACGCCTTTGTAATGATTTTCTTTATAGTAATACCAATTATGATTGGGGGCTTTGGGAACTGACTAATTCCTTTAATGATTGGGGCCCCCGACATGGCCTTCCCCCGAATGAACAACATGAGCTTCTGGCTTTTGCCCCCCTCATTCCTGCTCCTTCTCGCCTCTTCGGGCGTTGAGGCTGGGGCTGGGACAGGGTGGACTGTCTACCCACCATTGGCAGGAAACCTGGCCCACGCAGGCGCATCTGTCGACCTAACAATCTTTTCTCTCCACCTCGCCGGGATCTCTTCCATTCTTGGCGCCATTAACTTTATCACCACAATCCTAAACATGAAGCCACCCGCTATCTCGCAGTATCAAACGCCCCTCTTCGTATGGGCCGTTCTTATCACAGCCGTTCTCCTCCTTCTCTCACTGCCCGTCCTCGCCGCCGGCATCACCATGCTACTTACAGACC

HAP36 ATCCGCGCGGAG-CTCAGCCAGCCCGGCGCACTACTTGGGGACGACCAGATTTATAACGTAATTGTCACTGCCCACGCCTTTGTAATGATTTTCTTTATAGTAATACCAATTATGATTGGGGGCTTTGGGAACTGACTAATTCCCCTAATGATTGGGGCCCCCGACATGGCCTTCCCCCGAATGAACAACATGAGCTTCTGGCTTTTGCCCCCCTCATTCCTGCTCCTTCTCGCCTCTTCGGGCGTTGAGGCTGGGGCTGGGACAGGGTGGACTGTCTACCCACCATTGGCAGGAAACCTGGCCCACGCAGGCGCATCTGTCGACCTAACAATCTTTTCTCTCCACCTCGCCGGGATCTCTTCCATTCTTGGCGCCATTAACTTTATCACCACAATCCTAAACATGAAGCCACCCACTATCTCGCAGTATCAAACGCCCCTCTTCGTATGGGCCGTTCTTATCACAGCCGTTCTCCTCCTTCTCTCACTGCCCGTCCTCGCCGCCGGCATCACCATGCTACTTACAGACC

HAP37 ATCCGCGCGGAG-CTCAGCCAGCCCGGCGCACTACTTGGGGACGACCAGATTTATAACGTAATTGTCACTGCCCACGCCTTTGTAATGATTTTCTTTATAGTAATACCAATTATGATTGGGGGCTTTGGGAACTGACTAATTCCCCTAATGATTGGGGCCCCCGACATGGCCTTCCCCCGAATGAACAACATGAGCTTCTGGCTTTTGCCCCCCTCATTCCTGCTCCTCCTCGCCTCTTCGGGCGTTGAGGCTGGGGCTGGGACAGGGTGGACTGTCTACCCACCATTGGCAGGAAACCTGGCCCACGCAGGCGCATCTGTCGACCTAACAATCTTTTCTCTCCACCTCGCCGGGATCTCTTCCATTCTTGGCGCCATTAACTTTATCACCACAATCCTAAACATGAAGCCACCCGCTATCTCGCAGTATCAAACGCCCCTCTTCGTATGGGCCGTTCTTATCACAGCCGTTCTCCTCCTTCTCTCACTGCCCGTCCTCGCCGCCGGCATCACCATGCTACTTACAGACC

HAP38 ATCCGCGCGGAG-CTCAGCCAGCCCGGCGCACTACTTGGGGACGACCAGATTTATAACGTAATTGTCACTGCCCACGCCTTTGTAATGATTTTCTTTATAGTAATACCAATTATGATTGGGGGCTTTGGGAACTGACTAATTCCCCTAATGATTGGGGCCCCCGACATGGCCTTCCCCCGAATGAACAACATGAGCTTCTGGCTTTTGCCCCCCTCATTCCTGCTTCTTCTCGCCTCTTCGGGCGTTGAGGCTGGGGCTGGGACAGGGTGGACTGTCTACCCACCATTGGCAGGAAACCTGGCCCACGCAGGCGCATCTGTCGACCTAACAATCTTTTCTCTCCACCTCGCCGGGATCTCTTCCATTCTTGGCGCCATTAACTTTATCACCACAATCCTAAACATGAAGCCACCCGCTATCTCGCAGTATCAAACGCCCCTCTTCGTATGGGCCGTTCTTATCACAGCCGTTCTCCTCCTTCTCTCACTGCCCGTCCTCGCCGCCGGCATCACCATGCTACTTACAGACC

HAP39 ATCCGCGCGGAG-CTCAGCCAGCCCGGCGCACTACTTGGGGACGACCAGATTTATAACGTAATTGTCACTGCCCACGCCTTTGTAATGATTTTCTTTATAGTAATACCAATTATGATTGGGGGCTTTGGGAACTGACTAATTCCCCTAATGATTGGGGCCCCCGACATGGCCTTCCCCCGAATGAACAACATGAGCTTCTGGCTTTTGCCCCCCTCATTCCTGCTCCTTCTCGCCTCTTCGGGCGTTGAGGCTGGGGCTGGGACAGGGTGGACTGTCTACCCACCATTGGCAGGAAACCTGGCCCACGCAGGCGCATCTGTCGACCTAACAATCTTTTCTCTCCACCTCGCCGGGATTTCTTCCATTCTTGGCGCCATTAACTTTATCACCACAATCCTAAACATGAAGCCACCCGCTATCTCGCAGTATCAAACGCCCCTCTTCGTATGGGCCGTTCTTATCACAGCCGTTCTCCTCCTTCTCTCACTGCCCGTCCTCGCCGCCGGCATCACCATGCTACTTACAGACC

HAP40 ATCCGCGCGGAG-CTCAGCCAGCCCGGCGCACTACTTGGGGACGACCAGATTTATAACGTAATTGTCACTGCCCACGCCTTTGTAATGATTTTCTTTATAGTAATACCAATTATGATTGGGGGCTTTGGGAACTGACTAATTCCCCTAATGATTGGGGCCCCCGACATGGCCTTCCCCCGAATGAACAACATGAGCTTCTGACTTTTGCCCCCCTCATTCCTGCTCCTTCTCGCCTCTTCGGGCGTTGAGGCTGGGGCTGGGACAGGGTGGACTGTCTACCCACCATTGGCAGGAAACCTGGCCCACGCAGGCGCATCTGTCGACCTAACAATCTTTTCTCTCCACCTCGCCGGGATCTCTTCCATTCTTGGCGCCATTAACTTTATCACCACAATCCTAAACATGAAGCCACCCGCTATCTCGCAGTATCAAACGCCCCTCTTCGTATGGGCCGTTCTTATCACAGCCGTTCTCCTCCTTCTCTCACTGCCCGTCCTCGCCGCCGGCATCACCATGCTACTTACAGACC

HAP41 ATCCGCGCGGAG-CTCAGCCAGCCCGGCGCACTACTTGGGGACGACCAGATTTATAACGTAATTGTCACTGCCCACGCCTTTGTAATGATTTTCTTTATAGTAATACCAATTATGATTGGGGGCTTTGGGAACTGACTAATTCCCCTAATGATTGGGGCCCCCGACATGGCCTTCCCCCGAATGAACAACATGAGCTTCTGGCTTTTGCCCCCCTCATTCCTGCTCCTTCTCGCCTCTTCGGGCGTTGAGGCTGGGGCTGGGACAGGGTGGACTGTCTACCCACCATTGGCAGGAAACCTGGCCCACGCAGGCGCATCTGTCGACCTAACAATCTTTTCTCTCCACCTCGCCGGGATCTCTTCCATTCTTGGCGCCATTAACTTTATCACCACAATCCTAAACATGAAGCCACCCGCTATCTCGCAGTATCAAACGCCCCTCTTCGTATGGGCCGTTCTTATCACAGCCGTTCTCCTCCTTCTCTCACTGCCCGTCCTCGCCGCCGGCATCACCATGCTACTTACAGACC

HAP42 ATCCGCGCGGAA-CTCAGCCAGCCCGGCGCACTACTTGGGGACGACCAGATTTATAACGTAATTGTCACTGCCCACGCCTTTGTAATGATTTTCTTTATAGTAATACCAATTATGATTGGGGGCTTTGGGAACTGACTAATTCCCCTAATGATTGGGGCCCCCGACATGGCCTTCCCCCGAATGAACAACATGAGCTTCTGGCTTCTGCCCCCCTCATTCCTGCTCCTTCTCGCCTCTTCGGGCGTTGAGGCTGGGGCTGGGACAGGATGGACTGTCTACCCACCATTGGCAGGAAACCTGGCCCACGCAGGCGCATCTGTCGACCTAACAATCTTTTCTCTCCACCTCGCCGGGATTTCTTCCATTCTTGGCGCCATTAACTTTATCACCACAATCCTAAACATGAAGCCACCCGCTATCTCGCAGTATCAAACGCCCCTCTTCGTATGGGCCGTTCTTATCACAGCCGTTCTCCTCCTTCTCTCACTGCCCGTCCTCGCCGCCGGCATCACCATGCTACTTACAGACC

HAP43 ATCCGCGCGGAA-CTCAGCCAGCCCGGCGCACTACTTGGGGACGACCAGATTTATAACGTAATTGTCACTGCCCACGCCTTTGTAATGATTTTCTTTATAGTAATACCAATTATGATTGGGGGCTTTGGGAACTGACTAATTCCCCTAATGATTGGGGCCCCCGACATGGCCTTCCCCCGAATGAACAACATGAGCTTCTGGCTTTTGCCCCCCTCATTCCTGCTCCTTCTCGCCTCTTCGGGCGTTGAGGCTGGGGCTGGGACAGGATGGACTGTCTACCCACCATTGGCAGGAAACCTGGCCCACGCAGGCGCATCTGTCGACCTAACAATCTTTTCTCTCCACCTCGCCGGGATTTCTTCCATTCTTGGCGCCATTAACTTTATCACCACAATCCTAAACATGAAGCCACCCGCTATCTCGCAGTATCAAACGCCCCTCTTCGTATGGGCCGTTCTTATCACAGCCGTTCTCCTCCTTCTCTCACTGCCCGTCCTCGCCGCCGGCATCACCATGCTACTTACAGACC

HAP44 ATCCGCGCGGAA-CTCAGCCAGCCCGGCGCACTACTTGGGGACGACCAGATTTATAACGTAATTGTCACTGCCCACGCCTTTGTAATGATTTTCTTTATAGTAATACCAATTATGATTGGGGGCTTTGGGAACTGACTAATTCCCCTAATGATTGGGGCCCCCGACATGGCCTTCCCCCGAATGAACAACATGAGCTTCTGGCTTTTGCCCCCCTCATTCCTGCTCCTTCTCGCCTCTTCGGGCGTTGAGGCTGGGGCTGGGACAGGGTGGACTGTCTACCCACCATTGGCAGGAAACCTGGCCCACGCAGGCGCATCTGTCGACCTAACAATCTTTTCTCTCCACCTCGCCGGGATTTCTTCCATTCTTGGCGCCATTAACTTTATCACCACAATCCTAAACATGAAGCCACCCGCTATCTCGCAGTATCAAACGCCCCTCTTCGTATGGGCCGTTCTTATCACAGCCGTTCTCCTCCTTCTCTCACTGCCCGTCCTCGCCGCCGGCATCACTATGCTACTTACAGACC

HAP45 ATCCGCGCGGAA-CTCAGCCAGCCCGGCGCACTACTTGGGGACGACCAGATTTATAACGTAATTGTCACTGCCCACGCCTTTGTAATGATTTTCTTTATAGTAATACCAATTATGATTGGGGGCTTTGGGAACTGACTAATTCCCCTAATGATTGGGGCCCCCGACATGGCCTTCCCCCGAATGAACAACATGAGCTTCTGGCTTTTGCCCCCCTCATTCCTGCTCCTTCTCGCCTCTTCGGGCGTTGAGGCTGGGGCTGGGACAGGGTGGACTGTCTACCCACCATTGGCAGGAAACCTGGCCCACGCAGGCGCATCTGTCGACCTAACAATCTTTTCTCTCCACCTCGCCGGGATTTCTTCCATTCTTGGCGCCATTAACTTTATCACCACAATCCTAAACATGAAGCCACCCGCCATCTCGCAGTATCAAACGCCCCTCTTCGTATGGGCCGTTCTTATCACAGCCGTTCTCCTCCTTCTCTCACTGCCCGTCCTCGCCGCCGGCATCACCATGCTACTTACAGACC

HAP46 ATCCGCGCGGAA-CTCAGCCAGCCCGGCGCACTACTTGGGGACGACCAGATTTATAACGTAATTGTCACTGCCCACGCCTTTGTAATGATTTTCTTTATAGTAATACCAATTATGATTGGGGGCTTTGGGAACTGACTAATTCCCCTAATGATTGGGGCCCCCGACATGGCCTTCCCCCGAATGAACAACATGAGCTTCTGGCTTTTGCCCCCCTCATTCCTGCTCCTTCTCGCCTCTTCGGGCGTTGAGGCTGGGGCTGGGACAGGGTGGACTGTCTACCCACCATTGGCAGGAAACCTGGCCCACGCAGGCGCATCTGTCGACCTAACAATCTTTTCTCTCCACCTCGCCGGGATTTCTTCCATTCTTGGCGCCATTAACTTTATCACCACAATCCTAAACATGAAGCCACCCGCTATCTCGCAGTATCAAACGCCCCTCTTCGTTTGGGCCGTTCTTATCACAGCCGTTCTCCTCCTTCTCTCACTGCCCGTCCTCGCCGCCGGCATCACCATGCTACTTACAGACC

HAP47 ATCCGCGCGGAA-CTCAGCCAGCCCGGCGCACTACTTGGGGACGACCAGATTTATAACGTAATTGTCACTGCCCACGCCTTTGTAATGATTTTCTTTATAGTAATACCAATTATGATTGGGGGCTTTGGGAACTGACTAATTCCCCTAATGATTGGGGCCCCCGACATGGCCTTCCCCCGAATGAACAACATGAGCTTCTGGCTTTTGCCCCCCTCATTCCTGCTCCTTCTCGCCTCTTCGGGCGTTGAGGCTGGGGCTGGGACAGGGTGGACTGTCTACCCGCCATTGGCAGGAAACCTGGCCCACGCAGGCGCATCTGTCGACCTAACAATCTTTTCTCTCCACCTCGCCGGGATTTCTTCCATTCTTGGCGCCATTAACTTTATCACCACAATCCTAAACATGAAGCCACCCGCTATCTCGCAGTATCAAACGCCCCTCTTCGTATGGGCCGTTCTTATCACAGCCGTTCTCCTCCTTCTCTCACTGCCCGTCCTCGCCGCCGGCATCACCATGCTACTTACAGACC

HAP48 ATCCGCGCGGAA-CTCAGCCAGCCCGGCGCACTACTTGGGGACGACCAGATTTATAACGTAATTGTCACTGCCCACGCCTTTGTAATGATTTTCTTTATAGTAATACCAATTATGATTGGGGGCTTTGGGAACTGACTAATTCCCCTAATGATTGGGGCCCCCGACATGGCCTTCCCCCGAATGAACAACATGAGCTTCTGGCTTTTGCCCCCCTCATTCCTGCTCCTTCTCGCCTCTTCGGGCGTTGAGGCTGGGGCTGGGACAGGGTGGACTGTCTACCCACCATTGGCAGGAAACCTGGCCCACGCAGGCGCATCTGTCGACCTAACAATCTTTTCTCTCCACCTCGCCGGGATTTCTTCCATTCTTGGCGCCATTAACTTTATCACCACAATCCTAAACATGAAGCCACCCGCTATCTCGCAGTATCAAACGCCCCTCTTCGTATGGGCCGTTCTTATCACAGCCGTTCTCCTCCTTCTCTCACTGCCCGTCCTCGCCGCCGGCATCACCATGCTACTTACAGACC

HAP49 ATCCGCGCGGAA-CTCAGCCAGCCCGGCGCACTACTTGGGGACGACCAGATTTATAACGTAATTGTCACTGCCCACGCCTTTGTAATGATTTTCTTTATAGTAATACCAATTATGATTGGGGGCTTTGGGAACTGACTAATTCCCCTAATGATTGGGGCCCCCGACATGGCCTTCCCCCGAATGAACAACATGAGCTTCTGGCTTTTGCCCCCCTCATTCCTGCTCCTTCTCGCCTCTTCGGGCGTTGAGGCTGGGGCTGGGACAGGGTGGACTGTCTACCCACCGTTGGCAGGAAACCTGGCCCACGCAGGCGCATCTGTCGACCTAACAATCTTTTCTCTCCACCTCGCCGGGATCTCTTCCATTCTTGGCGCCATTAACTTTATCACCACAATCCTAAACATGAAGCCACCCGCTATCTCGCAGTATCAAACGCCCCTCTTCGTATGGGCCGTTCTTATCACAGCCGTTCTCCTCCTTCTCTCACTGCCCGTCCTCGCCGCCGGCATCACCATGCTACTTACAGACC

HAP50 ATCCGCGCGGAA-CTCAGCCAGCCCGGCGCACTACTTGGGGACGACCAGATTTATAACGTAATTGTCACTGCCCACGCCTTTGTAATGATTTTCTTTATAGTAATACCAATTATGATTGGGGGCTTTGGGAACTGACTAATTCCCCTAATGATTGGGGCCCCCGACATGGCCTTCCCCCGAATGAACAACATGAGCTTCTGGCTTTTGCCCCCCTCATTCCTGCTCCTTCTCGCCTCTTCGGGCGTTGAGGCTGGGGCTGGGACAGGGTGGACTGTCTACCCACCATTGGCAGGAAACCTGGCCCACGCAGGCGCATCTGTCGACCTAACAATCTTTTCTCTCCACCTCGCCGGGATCTCTTCCATTCTTGGTGCCATTAACTTTATCACCACAATCCTAAACATGAAGCCACCCGCTATCTCGCAGTATCAAACGCCCCTCTTCGTATGGGCCGTTCTTATCACAGCCGTTCTCCTCCTTCTCTCACTGCCCGTCCTCGCCGCCGGCATCACCATGCTACTTACAGACC

HAP51 ATCCGCGCGGAA-CTCAGCCAGCCCGGCGCACTACTTGGGGACGACCAGATTTATAACGTAATTGTCACTGCCCACGCCTTTGTAATGATTTTCTTTATAGTAATACCAATTATGATTGGGGGCTTTGGGAACTGACTAATTCCCCTAATGATTGGGGCCCCCGACATGGCCTTCCCCCGAATGAACAACATGAGCTTCTGGCTTTTGCCCCCCTCATTCCTGCTCCTTCTCGCCTCTTCGGGCGTTGAGGCTGGGGCTGGGACAGGGTGGACTGTCTACCCACCATTGGCAGGAAACCTGGCCCACGCAGGCGCATCTGTCGACCTAACAATCTTTTCTCTCCACCTCGCCGGGATCTCTTCCATTCTTGGCGCCATTAACTTTATCACCACAATCCTAAACATGAAGCCACCCGCTATCTCGCAGTATCAAACGCCCCTCTTCGTATGGGCCGTTCTTATCACAGCCGTTCTCCTCCTTCTCTCACTGCCCGTCCTCGCCGCCGGCATCACCATGCTACTTACAGACC

HAP52 ATCCGCGCGGAA-CTCAGCCAGCCCGGCGCACTACTTGGGGATGACCAGATTTATAACGTAATTGTTACTGCTCATGCCTTCGTAATAATTTTCTTTATAGTAATACCAATTATGATTGGAGGTTTTGGGAACTGACTGATTCCCCTAATGATTGGGGCCCCCGACATGGCCTTCCCCCGAATAAATAACATAAGCTTCTGGCTTTTACCCCCCTCATTCCTGCTCCTTCTCGCCTCTTCAGGCGTTGAAGCTGGGGCCGGAACAGGGTGAACTGTCTACCCCCCACTAGCAGGCAACCTAGCCCACGCAGGCGCATCTGTCGACCTAACAATTTTTTCTCTCCACCTCGCTGGGATCTCTTCCATTCTTGGGGCCATTAACTTTATCACCACAATCCTAAACATGAAGCCGCCCGCTGTCTCGCAGTATCAAACGCCCCTCTTCGTATGGGCCGTCCTTATCACAGCCGTCCTTCTCCTTCTTTCACTGCCCGTCCTCGCCGCCGGCATCACCATGCTACTCACAGACC

HAP53 ATCCGCGCGGAA-CTCAGCCAGCCCGGCGCACTACTTGGGGATGACCAGATTTATAACGTAATTGTTACTGCTCACGCCTTCGTAATAATTTTCTTTATAGTAATACCAATTATGATTGGAGGTTTTGGGAACTGACTGATTCCCCTAATGATTGGGGCCCCCGACATGGCCTTCCCCCGAATAAATAACATAAGCTTCTGGCTTTTACCCCCCTCATTCCTGCTCCTTCTCGCCTCTTCAGGCGTTGAAGCTGGGGCCGGAACAGGGTGAACTGTCTACCCCCCACTAGCAGGCAACCTAGCCCACGCAGGCGCATCTGTCGACCTAACAATTTTTTCTCTCCACCTCGCTGGGGTCTCTTCCATTCTTGGGGCCATTAACTTTATCACCACAATCCTAAACATGAAGCCGCCCGCTGTCTCGCAGTATCAAACGCCCCTCTTCGTATGGGCCGTCCTTATCACAGCCGTCCTTCTCCTTCTTTCACTGCCCGTCCTCGCCGCCGGCATCACCATGCTACTCACAGACC

HAP54 ATCCGCGCGGAA-CTCAGCCAGCCCGGCGCACTACTTGGGGATGACCAGATTTATAACGTAATTGTTACTGCTCACGCCTTCGTAATAATTTTCTTTATAGTAATACCAATTATGATTGGAGGTTTTGGGAACTGACTGATTCCCCTAATGATTGGGGCCCCCGACATGGCCTTCCCCCGAATAAATAACATAAGCTTCTGGCTTTTACCCCCCTCATTCCTGCTCCTTCTCGCCTCTTCAGGCGTTGAAGCTGGGGCCGGAACAGGGTGAACTGTCTACCCCCCACTAGCAGGCAACCTAGCCCACGCAGGCGCATCTGTCGACCTAACAATTTTTTCTCTCCACCTCGCTGGGATCTCTTCCATTCTTGGGGCCATTAACTTTATCACCACAATCCTAAACATGAAGCCGCCCGCTGTCTCGCAGTATCAAACGCCCCTCTTCGTATGGGCCGTCCTTATCACAGCTGTCCTTCTCCTTCTTTCACTGCCCGTCCTCGCCGCCGGCATCACCATGCTACTCACAGACC

HAP55 ATCCGCGCGGAA-CTCAGCCAGCCCGGCGCACTACTTGGGGATGACCAGATTTATAACGTAATTGTTACTGCTCACGCCTTCGTAATAATTTTCTTTATAGTAATACCAATTATGATTGGAGGTTTTGGGAACTGACTGATTCCCCTAATGATTGGGGCCCCCGACATGGCCTTCCCCCGAATAAATAACATAAGCTTCTGGCTTTTACCCCCCTCATTCCTGCTCCTTCTCGCCTCTTCAGGCGTTGAAGCTGGGGCCGGAACAGGGTGAACTGTCTACCCCCCACTAGCAGGCAACCTAGCCCACGCAGGCGCATCTGTCGACCTAACAATTTTTTCTCTCCACCTCGCTGGGATCTCTTCCATTCTTGGGGCCATTAACTTTATCACCACAATCCTAAACATGAAGCCGCCCGCTGTCTCGCAGTATCAAACGCCCCTCTTCGTATGGGCCGTCCTTATCACAGCCGTCCTTCTCCTCCTTTCACTGCCCGTCCTCGCCGCCGGCATCACCATGCTACTCACAGACC

HAP56 ATCCGCGCGGAA-CTCAGCCAGCCCGGCGCACTACTTGGGGATGACCAGATTTATAACGTAATTGTTACTGCTCACGCCTTCGTAATAATTTTCTTTATAGTAATACCAATTATGATTGGAGGTTTTGGGAACTGACTGATTCCCCTAATGATTGGGGCCCCAGACATGGCCTTCCCCCGAATAAATAACATAAGCTTCTGGCTTTTACCCCCCTCATTCCTGCTCCTTCTCGCCTCTTCAGGCGTTGAAGCTGGGGCCGGAACAGGGTGAACTGTCTACCCCCCACTAGCAGGCAACCTAGCCCACGCAGGCGCATCTGTCGACCTAACAATTTTTTCTCTCCACCTCGCTGGGATCTCTTCCATTCTTGGGGCCATTAACTTTATCACCACAATCCTAAACATGAAGCCGCCCGCTGTCTCGCAGTATCAAACGCCCCTCTTCGTATGGGCCGTCCTTATCACAGCCGTCCTTCTCCTTCTTTCACTGCCCGTCCTCGCCGCCGGCATCACCATGCTACTCACAGACC

HAP57 ATCCGCGCGGAA-CTCAGCCAGCCCGGCGCACTACTTGGGGATGACCAGATTTATAACGTAATTGTTACTGCTCACGCCTTCGTAATAATTTTCTTTATAGTAATACCAATTATGATTGGAGGTTTTGGGAACTGACTGATTCCCCTAATGATTGGGGCCCCCGACATGGCCTTCCCCCGAATAAATAACATAAGCTTCTGGCTTTTACCCCCCTCATTCCTGCTCCTTCTCGCCTCTTCAGGCGTTGAAGCTGGGGCCGGAACAGGGTGAACTGTCTACCCCCCACTAGCAGGCAACCTAGCCCACGCAGGCGCATCTGTCGACCTAACAATTTTTTCTCTCCACCTCGCTGGGATCTCTTCCATTCTTGGGGCCATTAACTTTATCACCACAATCCTAAACATGAAGCCGCCCGCTGTCTCGCAGTATCAAACGCCCCTCTTCGTATGGGCCGTCCTTATCACAGCCGTCCTTCTCCTTCTTTCACTGCCCGTCCTCGCCGCCGGCATCACCATGCTACTCACAGACC

HAP58 ATCCGCGCGGAA-CTCAGCCAGCCCGGCGCACTACTTGGGGATGACCAGATTTATAACGTAATTGTTACTGCTCACGCCTTCGTAATAATTTTCTTTATAGTAATACCAATTATGATTGGAGGTTTTGGGAACTGACTGATTCCCCTAATGATTGGGGCCCCCGACATGGCCTTCCCCCGAATAAATAACATAAGCTTCTGGCTTTTACCCCCCTCATTCCTGCTCCTTCTCGCCTCTTCAGGCGTTGAAGCTGGGGCCGGAACAGGGTGAACTGTCTACCCCCCACTAGCAGGCAACCTCGCCCACGCAGGCGCATCTGTCGACCTAACAATTTTTTCTCTCCACCTCGCTGGGATCTCTTCCATTCTTGGGGCCATTAACTTTATCACCACAATCCTAAACATGAAGCCGCCCGCTGTCTCGCAGTATCAAACGCCCCTCTTCGTATGGGCCGTCCTTATCACAGCCGTCCTTCTCCTTCTTTCACTGCCCGTCCTCGCCGCCGGCATCACCATGCTACTCACAGACC

HAP59 ATCCGCGCGGAA-CTCAGCCAGCCCGGCGCACTACTTGGGGATGACCAGATTTATAACGTAATTGTTACTGCTCACGCCTTCGTAATAATTTTCTTTATAGTAATACCAATTATGATTGGAGGTTTTGGGAACTGACTGATTCCCCTAATGATTGGGGCCCCCGACATGGCCTTCCCCCGAATAAATAACATAAGCTTCTGGCTTTTACCCCCCTCATTCCTGCTCCTTCTCGCCTCTTCAGGCGTTGAAGCTGGGGCCGGAACAGGGTGAACTGTCTACCCCCCACTAGCAGGCAACCTGGCCCACGCAGGCGCATCTGTCGACCTAACAATTTTTTCTCTCCACCTCGCTGGGATCTCTTCCATTCTTGGGGCCATTAACTTTATCACCACAATCCTAAACATGAAGCCGCCCGCTGTCTCGCAGTATCAAACGCCCCTCTTCGTATGGGCCGTCCTTATCACAGCCGTCCTTCTCCTTCTTTCACTGCCCGTCCTCGCCGCCGGCATCACCATGCTACTCACAGACC

HAP60 ATCCGCGCGGAA-CTCAGCCAGCCCGGCGCACTACTTGGGGATGACCAGATTTATAACGTAATTGTTACTGCTCACGCCTTCGTAATAATTTTCTTTATAGTAATACCAATTATGATTGGAGGTTTTGGGAACTGACTGATTCCCCTAATGATTGGGGCCCCCGACATGGCCTTCCCCCGAATAAATAACATAAGCTTCTGGCTTTTACCCCCCTCATTCCTGCTCCTTCTCGCCTCTTCAGGCGTTGAAGCTGGGGCCGGAACAGGGTGAACTGTCTACCCCCCACTAGCAGGCAACCTAGCCCACGCAGGCGCATCTGTCGACCTAACAATTTTTTCTCTCCACCTCGCTGGGATCTCTTCCATTCTTGGGGCCATTAACTTTATCACCACAATCCTAAACATGAAGCCACCCGCTGTCTCGCAGTATCAAACGCCCCTCTTCGTATGGGCCGTCCTTATCACAGCCGTCCTTCTCCTTCTTTCACTGCCCGTCCTCGCCGCCGGCATCACCATGCTACTCACAGACC

HAP61 ATCCGCGCGGAA-CTCAGCCAGCCCGGCGCACTACTTGGGGATGACCAGATTTATAACGTAATTGTTACTGCTCACGCCTTCGTAATAATTTTCTTTATAGTAATACCAATTATGATTGGAGGTTTTGGGAACTGACTGATTCCCCTAATGATTGGGGCCCCCGACATGGCCTTCCCCCGAATAAATAACATAAGCTTCTGGCTTTTACCCCCCTCATTCCTGCTCCTTCTCGCCTCTTCAGGCGTTGAAGCTGGGGCCGGAACAGGGTGAACTGTCTACCCCCCACTAGCAGGCAACCTAGCCCACGCAGGCGCATCTGTCGACCTAACAATTTTTTCTCTCCACCTCGCTGGGATCTCTTCCATTCTTGGGGCCATTAACTTTATCACCACAATCCTAAACATGAAGCCGCCCGCTGTCTCGCAGTATCAAACGCCCCTCTTCGTATGGGCCGTCCTTATCACAGCCGTCCTCCTCCTTCTTTCACTGCCCGTCCTCGCCGCCGGCATCACCATGCTACTCACAGACC

HAP62 ATCCGCGCGGAA-CTCAGCCAGCCCGGCGCACTACTTGGGGATGACCAGATTTATAACGTAATTGTTACTGCTCACGCCTTCGTAATAATTTTCTTTATAGTAATACCAATTATGATTGGAGGTTTTGGGAACTGACTGATTCCCCTAATGATTGGGGCCCCCGACATGGCCTTCCCCCGAATAAATAACATAAGCTTCTGGCTTTTACCCCCCTCATTCCTGCTCCTTCTCGCCTCTTCAGGCGTTGAAGCTGGGGCCGGAACAGGGTGAACTGTCTACCCCCCACTAGCAGGCAACCTAGCCCACGCAGGCGCATCTGTCGACCTAACAATTTTTTCTCTCCACCTCGCCGGGATCTCTTCCATTCTTGGAGCCATTAACTTTATCACCACAATCCTAAACATGAAGCCGCCCGCTGTCTCGCAGTATCAAACGCCCCTCTTCGTATGGGCCGTCCTTATCACAGCCGTCCTTCTCCTTCTTTCACTGCCCGTCCTCGCCGCCGGCATCACCATGCTACTCACAGACC

HAP63 ATCCGCGCGGAA-CTCAGCCAGCCCGGCGCACTACTTGGAGATGACCAGATTTATAACGTAATTGTTACTGCTCACGCCTTCGTAATGATTTTCTTTATAGTAATACCAATTATGATTGGAGGGTTTGGGAACTGACTGGTTCCTCTAATGATTGGGGCCCCCGACATGGCCTTCCCACGAATGAATAACATGAGCTTCTGGCTTTTACCCCCCTCATTCCTGCTCCTCCTCGCCTCTTCAGGCGTTGAAGCTGGGGCTGGGACAGGGTGGACCGTCTACCCCCCACTGGCAGGCAATCTAGCCCACGCAGGCGCATCTGTCGACCTAACAATCTTTTCTCTCCACCTCGCCGGGATCTCTTCCATTCTCGGGGCCATTAACTTTATCACCACAATCCTAAACATAAAACCGCCCGCTGTCTCGCAGTATCAAACGCCCCTCTTCGTGTGGGCCGTCCTTATCACAGCCGTCCTTCTCCTTCTTTCACTGCCCGTCCTCGCCGCCGGCATCACCATGCTACTTACAGACC

HAP64 ATCCGCGCGGAA-CTCAGCCAGCCCGGCGCACTACTTGGAGATGACCAGATTTATAACGTAATTGTTACTGCTCACGCCTTCGTAATGATTTTCTTTATAGTAATACCAATTATGATTGGAGGGTTTGGGAACTGACTGGTTCCTCTAATGATTGGGGCCCCCGACATGGCCTTCCCACGAATGAATAACATGAGCTTCTGGCTTTTACCCCCCTCATTCCTGCTCCTCCTCGCCTCTTCAGGCGTTGAAGCTGGGGCTGGGACAGGGTGGACCGTCTACCCCCCACTGGCAGGCAATCTAGCCCACGCAGGCGCATCTGTCGACCTAACAATCTTTTCTCTCCACCTCGCCGGGATCTCTTCCATTCTCGGGGCCATTAACTTTATCACCACAATCCTAAACATGAAACCGCCCGCTGTCTCGCAGTATCAAACGCCCCTCTTCGTGTGGGCCGTCCTTATCACAGCCGTCCTTCTCCTTCTTTCACTGCCCGTCCTCGCCGCCGGCATCACCATGTTACTTACAGACC

HAP65 ATCCGCGCGGAA-CTCAGCCAGCCCGGCGCACTACTTGGAGATGACCAGATTTATAATGTAATTGTTACTGCTCACGCCTTCGTAATGATTTTCTTTATAGTAATACCAATTATGATTGGAGGGTTTGGGAACTGACTGGTTCCTCTAATGATTGGGGCCCCCGACATGGCCTTCCCACGAATGAATAACATGAGCTTCTGGCTTTTACCCCCCTCATTCCTGCTCCTCCTCGCCTCTTCAGGCGTTGAAGCTGGGGCTGGGACAGGGTGGACCGTCTACCCCCCACTGGCAGGCAATCTAGCCCACGCAGGCGCATCTGTCGACCTAACAATCTTTTCTCTCCACCTCGCCGGGATCTCTTCCATTCTCGGGGCCATTAACTTTATCACCACAATCCTAAACATGAAACCGCCCGCTGTCTCGCAGTATCAAACGCCCCTCTTCGTGTGGGCCGTCCTTATCACAGCCGTCCTTCTCCTTCTTTCACTGCCCGTCCTCGCCGCCGGCATCACCATGCTACTTACAGACC

HAP66 ATCCGCGCGGAA-CTCAGCCAGCCCGGCGCACTACTTGGAGATGACCAGATTTATAACGTAATTGTTACTGCTCACGCCTTCGTAATGATTTTCTTTATAGTAATACCAATTATGATTGGAGGGTTTGGGAACTGACTGGTTCCTCTAATGATTGGGGCCCCCGACATGGCCTTCCCACGAATGAATAACATGAGCTTCTGGCTTTTACCCCCCTCATTCCTGCTCCTCCTCGCCTCTTCAGGCGTTGAAGCTGGGGCTGGGACAGGGTGGACCGTCTACCCCCCACTGGCAGGCAATCTAGCCCACGCAGGCGCATCTGTCGACCTAACAATCTTTTCTCTCCACCTCGCCGGGATCTCTTCCATTCTCGGGGCCATTAACTTTATCACCACAATCCTAAACATGAAACCGCCCGCTGTCTCGCAGTATCAAACGCCCCTCTTCGTGTGGGCCGTCCTTATCACAGCCGTCCTTCTCCTTCTTTCACTGCCCGTCCTCGCCGCCGGCATCACCATGCTACTTACAGACC

HAP67 ATCCGCGCGGAA-CTCAGCCAGCCCGGCGCACTACTTGGAGATGACCAGATTTATAACGTAATTGTTACTGCTCACGCCTTCGTAATGATTTTCTTTATAGTAATACCAATTATGATTGGAGGGTTTGGGAACTGACTGGTTCCTCTAATGATTGGGGCCCCCGACATGGCCTTCCCACGAATGAATAACATGAGCTTCTGGCTTTTACCCCCCTCATTCCTGCTCCTCCTCGCCTCTTCAGGCGTTGAAGCTGGGGCTGGGACAGGGTGGACCGTCTACCCCCCACTGGCAGGCAATCTAGCCCACGCAGGCGCATCTGTCGACCTAACAATCTTTTCTCTCCACCTCGCCGGGATCTCTTCCATTCTCGGAGCCATTAACTTTATCACCACAATCCTAAACATGAAACCGCCCGCTGTCTCGCAGTATCAAACGCCCCTCTTCGTGTGGGCCGTCCTTATCACAGCCGTCCTTCTCCTTCTTTCACTGCCCGTCCTCGCCGCCGGCATCACCATGCTACTTACAGACC

HAP68 ATCCGCGCGGAA-CTCAGCCAGCCCGGCGCACTACTTGGAGATGACCAGATTTATAACGTAATTGTTACTGCTCACGCCTTCGTAATGATTTTCTTTATAGTAATACCAATTATGATTGGAGGGTTTGGGAACTGACTGATTCCTCTAATGATTGGGGCCCCCGACATGGCCTTCCCACGAATGAATAACATGAGCTTCTGGCTTTTACCCCCCTCATTCCTGCTCCTCCTCGCCTCTTCAGGCGTTGAAGCTGGGGCTGGGACAGGGTGAACCGTCTACCCCCCACTGGCAGGCAATCTAGCCCACGCAGGCGCATCTGTCGACCTAACAATCTTTTCTCTCCACCTCGCCGGGATCTCTTCCATTCTCGGGGCCATTAACTTTATCACCACAATCCTAAACATGAAACCGCCCGCTGTCTCGCAGTATCAAACGCCCCTCTTCGTGTGGGCCGTCCTTATCACAGCCGTCCTTCTCCTTCTTTCACTGCCCGTCCTCGCCGCCGGCATCACCATGCTACTTACAGACC

HAP69 ATCCGCGCGGAA-CTCAGCCAGCCCGGCGCACTACTTGGAGATGACCAGATTTATAACGTAATTGTTACTGCTCACGCCTTCGTAATGATTTTCTTTATAGTAATACCAATTATGATTGGAGGGTTTGGGAACTGACTGATTCCTCTAATGATTGGGGCCCCCGACATGGCCTTCCCACGAATGAATAACATGAGCTTCTGGCTTTTACCCCCCTCATTCCTGCTCCTCCTCGCCTCTTCAGGCGTTGAAGCTGGGGCTGGGACAGGGTGGACCGTCTACCCCCCACTGGCAGGCAATCTAGCCCACGCAGGCGCATCTGTCGACCTAACAATCTTTTCTCTCCACCTCGCCGGGATCTCTTCCATTCTCGGGGCCATTAACTTTATCACCACAATCCTAAACATGAAACCGCCCGCTGTCTCGCAGTATCAAACGCCCCTCTTCGTGTGGGCCGTCCTTATCACAGCCGTCCTTCTCCTTCTTTCACTGCCCGTCCTCGCCGCCGGCATCACCATGCTACTTACAGACC

HAP70 ATCCGCGCGGAA-CTCAGCCAGCCCGGCGCACTACTTGGAGATGACCAGATTTATAACGTAATTGTTACTGCTCACGCCTTCGTAATGATTTTCTTTATAGTAATACCAATTATGATTGGAGGGTTTGGGAACTGACTGATTCCTCTAATGATTGGGGCCCCCGACATGGCCTTCCCACGAATGAATAACATGAGCTTCTGGCTTTTACCCCCCTCATTCCTGCTCCTCCTCGCCTCTTCAGGCGTTGAAGCTGGGGCTGGGACAGGGTGGACCGTCTACCCCCCACTGGCAGGCAATCTAGCCCACGCAGGCGCATCTGTCGACCTGACAATCTTTTCTCTCCACCTCGCCGGGATCTCTTCCATTCTCGGGGCCATTAACTTTATCACCACTATCCTAAACATGAAACCGCCCGCTGTCTCGCAGTATCAAACGCCCCTCTTCGTGTGGGCCGTCCTTATCACAGCCGTCCTTCTCCTTCTTTCACTGCCCGTCCTCGCCGCCGGCATCACCATGCTACTTACAGACC

HAP71 ATCCGCGCGGAA-CTCAGCCAGCCCGGCGCACTACTTGGAGATGACCAGATTTATAACGTAATTGTTACTGCTCACGCCTTCGTAATGATTTTCTTTATAGTAATACCAATTATGATTGGAGGGTTTGGGAACTGACTGATTCCTCTAATGATTGGGGCCCCCGACATGGCCTTCCCACGAATGAATAACATGAGCTTCTGGCTTTTGCCCCCCTCATTCCTGCTCCTCCTCGCCTCTTCAGGCGTTGAAGCTGGGGCTGGGACAGGGTGGACCGTCTACCCCCCACTGGCAGGCAATCTAGCCCACGCAGGCGCATCTGTCGACCTGACAATCTTTTCTCTCCACCTCGCCGGGATCTCTTCCATTCTCGGGGCCATTAACTTTATCACCACTATCCTAAACATGAAACCGCCCGCTGTCTCGCAGTATCAAACGCCCCTCTTCGTGTGGGCCGTCCTTATCACAGCCGTCCTTCTCCTTCTTTCACTGCCCGTCCTCGCCGCCGGCATCACCATGCTACTTACAGACC

HAP72 ATCCGCGCGGAA-CTCAGCCAGCCCGGCGCACTACTTGGAGATGACCAGATTTATAACGTAATTGTTACTGCTCACGCCTTCGTAATGATTTTCTTTATAGTAATACCAATTATGATTGGAGGGTTTGGGAACTGACTGATTCCTCTAATGATTGGGGCCCCCGACATGGCCTTCCCACGAATGAATAACATGAGCTTCTGGCTTTTGCCCCCATCATTCCTGCTCCTCCTCGCCTCTTCAGGCGTTGAAGCTGGGGCTGGGACAGGGTGGACCGTCTACCCCCCACTGGCAGGCAATCTAGCCCACGCAGGCGCATCTGTCGACCTAACAATCTTTTCTCTCCACCTCGCCGGGATCTCTTCCATTCTCGGGGCCATTAACTTTATCACCACAATCCTAAACATGAAACCGCCCGCTGTATCGCAGTATCAGACGCCCCTCTTCGTGTGGGCCGTCCTTATCACAGCCGTCCTTCTCCTTCTTTCACTGCCCGTCCTCGCCGCCGGCATCACCATGCTACTTACAGACC

HAP73 ATCCGCGCGGAA-CTCAGCCAGCCCGGCGCACTACTTGGAGATGACCAGATTTATAACGTAATTGTTACTGCTCACGCCTTCGTAATGATTTTCTTTATAGTAATACCAATTATGATTGGAGGGTTTGGGAACTGACTGATTCCTCTAATGATTGGGGCCCCCGACATGGCCTTCCCACGAATGAATAACATGAGCTTCTGGCTTTTGCCCCCATCATTCCTGCTCCTCCTCGCCTCTTCAGGCGTTGAAGCTGGGGCTGGGACAGGGTGGACCGTCTACCCCCCACTGGCAGGCAATCTAGCCCACGCAGGCGCATCTGTCGACCTAACAATCTTTTCTCTCCACCTCGCCGGGATCTCTTCCATTCTCGGGGCCATTAACTTTATCACCACAATTCTAAACATGAAACCGCCCGCTGTATCGCAGTATCAAACGCCCCTCTTCGTGTGGGCCGTCCTTATCACAGCCGTCCTTCTCCTTCTTTCACTGCCCGTCCTCGCCGCCGGCATCACCATGCTACTTACAGACC

HAP74 ATCCGCGCGGAA-CTCAGCCAGCCCGGCGCACTACTTGGAGATGACCAGATTTATAACGTAATTGTTACTGCTCACGCCTTCGTAATGATTTTCTTTATAGTAATACCAATTATGATTGGAGGGTTTGGGAACTGACTGATTCCTCTAATGATTGGGGCCCCCGACATGGCCTTCCCACGAATGAATAACATGAGCTTCTGGCTTTTGCCCCCATCATTCCTGCTCCTCCTCGCCTCTTCAGGCGTTGAAGCTGGGGCTGGGACAGGGTGGACCGTCTACCCCCCACTGGCAGGCAATCTAGCCCACGCAGGCGCATCTGTCGACCTAACAATCTTTTCTCTCCACCTCGCCGGGATCTCTTCCATTCTCGGGGCCATTAACTTTATTACCACAATCCTAAACATGAAACCGCCCGCTGTATCGCAGTATCAAACGCCCCTCTTCGTGTGGGCCGTCCTTATCACAGCCGTCCTTCTCCTTCTTTCACTGCCCGTCCTCGCCGCCGGCATCACCATGCTACTTACAGACC

HAP75 ATCCGCGCGGAA-CTCAGCCAGCCCGGCGCACTACTTGGAGATGACCAGATTTATAACGTAATTGTTACTGCTCACGCCTTCGTAATGATTTTCTTTATAGTAATACCAATTATGATTGGAGGGTTTGGGAACTGACTGATTCCTCTAATGATTGGGGCCCCCGACATGGCCTTCCCACGAATGAATAACATGAGCTTCTGGCTTTTGCCCCCATCATTCCTGCTCCTCCTCGCCTCTTCAGGTGTTGAAGCTGGGGCTGGGACAGGGTGGACCGTCTACCCCCCACTGGCAGGCAATCTAGCCCACGCAGGCGCATCTGTCGACCTAACAATCTTTTCTCTCCACCTCGCCGGGATCTCTTCCATTCTCGGGGCCATTAACTTTATCACCACAATCCTAAACATGAAACCGCCCGCTGTATCGCAGTATCAAACGCCCCTCTTCGTGTGGGCCGTCCTTATCACAGCCGTCCTTCTCCTTCTTTCACTGCCCGTCCTCGCCGCCGGCATCACCATGCTACTTACAGACC

HAP76 ATCCGCGCGGAA-CTCAGCCAGCCCGGCGCACTACTTGGAGATGACCAGATTTATAACGTAATTGTTACTGCTCACGCCTTCGTAATGATTTTCTTTATAGTAATACCAATTATGATTGGAGGGTTTGGGAACTGACTGATTCCTCTAATGATTGGGGCCCCCGACATGGCCTTCCCACGAATGAATAACATGAGCTTCTGGCTTTTGCCCCCATCATTCCTGCTCCTCCTCGCCTCTTCAGGCGTTGAAGCTGGGGCTGGGACAGGGTGGACCGTCTACCCCCCACTGGCGGGCAATCTAGCCCACGCAGGCGCATCTGTCGACCTAACAATCTTTTCTCTCCACCTCGCCGGGATCTCTTCCATTCTCGGGGCCATTAACTTTATCACCACAATCCTAAACATGAAACCGCCCGCTGTATCGCAGTATCAAACGCCCCTCTTCGTGTGGGCCGTCCTTATCACAGCCGTCCTTCTCCTTCTTTCACTGCCCGTCCTCGCCGCCGGCATCACCATGCTACTTACAGACC

HAP77 ATCCGCGCGGAA-CTCAGCCAGCCCGGCGCACTACTTGGAGATGACCAGATTTATAACGTAATTGTTACTGCTCACGCCTTCGTAATGATTTTCTTTATAGTAATACCAATTATGATTGGAGGGTTTGGAAACTGACTGATTCCTCTAATGATTGGGGCCCCCGACATGGCCTTCCCACGAATGAATAACATGAGCTTCTGGCTTTTGCCCCCATCATTCCTGCTCCTCCTCGCCTCTTCAGGCGTTGAAGCTGGGGCTGGGACAGGGTGGACCGTCTACCCCCCACTGGCAGGCAATCTAGCCCACGCAGGCGCATCTGTCGACCTAACAATCTTTTCTCTCCACCTCGCCGGGATCTCTTCCATTCTCGGGGCCATTAACTTTATCACCACAATCCTAAACATGAAACCGCCCGCTGTATCGCAGTATCAAACGCCCCTCTTCGTGTGGGCCGTCCTTATCACAGCCGTCCTTCTCCTTCTTTCACTGCCCGTCCTCGCCGCCGGCATCACCATGCTACTTACAGACC

HAP78 ATCCGCGCGGAA-CTCAGCCAGCCCGGCGCACTACTTGGAGATGACCAGATTTATAACGTAATTGTTACTGCTCACGCCTTCGTAATGATTTTCTTTATAGTAATACCAATTATGATTGGAGGGTTTGGGAACTGACTGATTCCTCTAATGATTGGGGCCCCCGACATGGCCTTCCCACGAATGAATAACATGAGCTTCTGGCTTTTGCCCCCATCATTCCTGCTCCTCCTCGCCTCTTCAGGCGTTGAAGCTGGGGCTGGGACAGGGTGGACCGTCTACCCCCCACTGGCAGGCAATCTAGCCCACGCAGGCGCATCTGTCGACCTAACAATCTTTTCTCTCCACCTCGCCGGGATCTCTTCCATTCTCGGGGCCATTAACTTTATCACCACAATCCTAAACATGAAACCGCCCGCTGTATCGCAGTATCAAACGCCCCTCTTCGTGTGGGCCGTCCTTATCACAGCCGTCCTTCTCCTTCTTTCACTGCCCGTCCTCGCCGCCGGCATCACCATGCTACTTACAGACC

HAP79 ATCCGCGCGGAA-CTCAGCCAGCCCGGCGCACTACTTGGAGACGACCAGATTTATAACGTAATTGTTACTGCTCACGCCTTCGTAATGATTTTCTTTATAGTAATACCAATTATGATTGGAGGGTTTGGGAACTGACTGATTCCTCTAATGATTGGGGCCCCCGACATGGCCTTCCCACGAATGAATAACATGAGCTTCTGGCTTTTGCCCCCATCATTCCTGCTCCTCCTCGCCTCTTCAGGCGTTGAAGCTGGGGCTGGGACAGGGTGGACCGTCTACCCCCCACTGGCAGGCAATCTAGCCCACGCAGGCGCATCTGTCGACCTAACAATCTTTTCTCTCCACCTCGCCGGGATCTCTTCCATTCTCGGGGCCATTAACTTTATCACCACAATCCTAAACATGAAACCGCCCGCTGTATCGCAGTATCAAACGCCCCTCTTCGTGTGGGCCGTCCTTATCACAGCCGTCCTTCTCCTTCTTTCACTGCCCGTCCTCGCCGCCGGCATCACCATGCTACTTACAGACC

HAP80 ATCCGCGCGGAA-CTCAGCCAGCCCGGCGCACTACTTGGAGATGACCAGATTTATAACGTAATTGTTACTGCTCACGCCTTCGTAATAATTTTCTTTATAGTAATACCAATTATGATTGGAGGGTTTGGGAACTGACTGATTCCTCTAATGATTGGGGCCCCCGACATGGCCTTCCCACGAATGAATAACATGAGCTTCTGGCTTTTGCCCCCATCATTCCTGCTCCTCCTCGCCTCTTCAGGCGTTGAAGCTGGGGCTGGGACAGGGTGGACCGTCTACCCCCCACTGGCAGGCAATCTAGCCCACGCAGGTGCATCTGTCGACCTAACAATCTTTTCTCTCCACCTCGCCGGGATCTCTTCCATTCTCGGGGCCATTAACTTTATCACCACAATCCTAAACATGAAACCGCCCGCTGTCTCGCAGTATCAAACGCCCCTCTTCGTGTGGGCCGTCCTTATCACAGCCGTCCTTCTCCTTCTTTCACTGCCCGTCCTCGCCGCCGGCATCACCATGCTACTTACAGACC

HAP81 ATCCGCGCGGAA-CTCAGCCAGCCCGGCGCACTACTTGGAGATGACCAGATTTATAACGTAATTGTTACTGCTCACGCCTTCGTAATAATTTTCTTTATAGTAATACCAATTATGATTGGAGGGTTTGGGAACTGACTGATTCCTCTAATGATTGGGGCCCCCGACATGGCCTTCCCACGAATAAATAACATGAGCTTCTGGCTTTTGCCCCCATCATTCCTGCTCCTCCTCGCCTCTTCAGGCGTTGAAGCTGGGGCTGGGACAGGGTGGACCGTCTACCCCCCACTGGCAGGCAATCTAGCCCACGCAGGTGCATCTGTCGACCTAACAATCTTTTCTCTCCACCTCGCCGGGATCTCTTCCATTCTCGGGGCCATTAACTTTATCACCACAATCCTAAACATGAAACCGCCCGCTGTCTCGCAGTATCAAACGCCCCTCTTCGTGTGGGCCGTCCTTATCACAGCCGTCCTTCTCCTTCTTTCACTGCCCGTCCTCGCCGCCGGCATCACCATGCTACTTACAGACC

HAP82 ATCCGCGCGGAA-CTCAGCCAGCCCGGCGCACTACTTGGAGATGACCAGATTTATAACGTAATTGTTACTGCTCACGCCTTCGTAATAATTTTCTTTATAGTAATACCAATTATGATTGGAGGGTTTGGGAACTGACTGATTCCTCTAATGATTGGGGCCCCCGACATGGCCTTCCCACGAATGAATAACATGAGCTTCTGGCTTTTGCCCCCCTCATTCCTGCTCCTCCTCGCCTCTTCAGGCGTTGAAGCTGGGGCTGGGACAGGGTGGACCGTCTACCCCCCACTGGCAGGCAATCTAGCCCACGCAGGTGCATCTGTCGACCTAACAATCTTTTCTCTCCACCTCGCCGGGATCTCTTCCATTCTCGGGGCCATTAACTTTATCACCACAATCCTAAACATGAAACCGCCCGCTGTCTCGCAGTATCAAACGCCCCTCTTCGTGTGGGCCGTCCTTATCACAGCCGTCCTTCTCCTTCTTTCACTGCCCGTCCTCGCCGCCGGCATCACCATGCTACTTACAGACC

HAP83 ATCCGCGCGGAA-CTCAGCCAGCCCGGCGCACTACTTGGAGATGACCAGATTTATAACGTAATTGTTACTGCTCACGCCTTCGTAATAATTTTCTTTATAGTAATACCAATTATGATTGGAGGGTTTGGGAACTGGCTGATTCCTCTAATGATTGGGGCCCCCGACATGGCCTTCCCACGAATGAATAACATGAGCTTCTGGCTTTTGCCCCCATCATTCCTGCTCCTCCTCGCCTCTTCAGGCGTTGAAGCTGGGGCTGGGACAGGGTGGACCGTCTACCCCCCACTGGCAGGCAATCTAGCCCACGCAGGCGCATCTGTCGACCTAACAATCTTTTCTCTCCACCTCGCCGGGATCTCTTCCATTCTCGGGGCCATTAACTTTATCACCACAATCCTAAACATGAAACCGCCCGCTGTCTCGCAGTATCAAACGCCCCTCTTCGTGTGGGCCGTCCTTATCACAGCCGTCCTTCTCCTTCTTTCACTGCCCGTCCTCGCCGCCGGCATCACCATGCTACTTACAGACC

HAP84 ATCCGCGCGGAA-CTCAGCCAGCCCGGCGCACTACTTGGAGATGACCAGATTTATAACGTAATTGTTACTGCTCACGCCTTCGTAATAATTTTCTTTATAGTAATACCAATTATGATTGGAGGGTTTGGGAACTGACTGATTCCTCTAATGATTGGGGCCCCCGACATGGCCTTCCCACGAATGAATAACATGAGCTTCTGGCTTTTGCCCCCATCATTCCTGCTCCTCCTCGCCTCTTCAGGCGTTGAAGCTGGGGCTGGGACAGGGTGGACCGTCTACCCCCCACTGGCAGGCAATCTAGCCCACGCAGGCGCATCTGTCGACCTAACAATCTTTTCTCTCCACCTCGCCGGGATCTCTTCCATTCTCGGGGCCATTAACTTTATCACCACAATCCTAAACATGAAACCGCCCGCTGTCTCGCAGTATCAAACGCCCCTCTTCGTGTGGGCCGTCCTTATCACAGCCGTCCTTCTCCTTCTTTCACTGCCCGTCCTCGCCGCCGGCATCACCATGCTACTTACAGACC

HAP85 ATCCGCGCGGAA-CTCAGCCAGCCCGGCGCACTACTTGGAGATGACCAGATTTATAACGTAATTGTTACTGCTCACGCCTTCGTAATAATTTTCTTTATAGTAATACCAATTATGATTGGAGGGTTTGGGAACTGACTGATTCCTCTAATGATTGGGGCCCCCGACATGGCCTTCCCACGAATGAATAACATGAGCTTCTGGCTTTTGCCCCCATCATTCCTGCTCCTCCTCGCCTCTTCAGGCGTTGAAGCTGGGGCTGGGACAGGGTGGACCGTCTACCCCCCACTGGCAGGCAATCTGGCCCACGCAGGCGCATCTGTCGACCTAACAATCTTTTCTCTCCACCTCGCCGGGATCTCTTCCATTCTCGGGGCCATTAATTTTATCACCACAATCCTAAACATGAAACCGCCCGCTGTCTCGCAGTATCAAACGCCCCTCTTCGTGTGGGCCGTCCTTATCACAGCCGTCCTTCTCCTTCTTTCACTGCCCGTCCTCGCCGCCGGCATCACCATGCTACTTACAGACC

HAP86 ATCCGCGCGGAA-CTCAGCCAGCCCGGCGCACTACTTGGAGATGACCAGATTTATAACGTAATTGTTACTGCTCACGCCTTCGTAATAATTTTCTTTATAGTAATACCAATTATGATTGGAGGGTTTGGGAACTGACTGATTCCTCTAATGATTGGGGCCCCCGACATGGCCTTCCCACGAATGAATAACATGAGCTTCTGGCTTTTGCCCCCATCATTCCTGCTCCTCCTCGCCTCTTCAGGCGTTGAAGCTGGGGCTGGGACAGGGTGGACCGTCTACCCCCCACTGGCAGGCAATCTGGCCCACGCAGGCGCATCTGTCGACCTAACAATCTTTTCTCTCCACCTCGCCGGGATCTCTTCCATTCTCGGGGCCATTAACTTTATCACCACAATCCTAAACATGAAACCGCCCGCTGTCTCGCAGTATCAAACGCCCCTCTTCGTGTGGGCCGTCCTTATCACAGCCGTCCTTCTCCTTCTTTCACTGCCCGTCCTCGCCGCCGGCATCACCATGCTACTTACAGACC

HAP87 ATCCGCGCGGAA-CTCAGCCAGCCCGGCGCACTACTTGGAGATGACCAGATTTATAACGTAATTGTTACTGCTCACGCCTTCGTAATGATTTTCTTTATAGTAATACCAATTATGATTGGAGGGTTTGGGAACTGACTGATTCCTCTAATGATTGGGGCCCCCGACATGGCCTTCCCACGAATGAATAACATGAGCTTCTGGCTTTTGCCCCCATCATTCCTGCTCCTCCTCGCCTCTTCAGGCGTTGAAGCTGGGGCTGGGACAGGGTGGACCGTCTACCCCCCACTGGCAGGCAATCTAGCCCACGCAGGCGCATCTGTCGACCTAACAATCTTTTCTCTCCACCTCGCCGGGATCTCTTCCATTCTCGGGGCCATTAACTTTATCACCACAATCCTAAACATGAAACCGCCCGCTGTCTCGCAGTATCAAACGCCCCTCTTCGTGTGGGCCGTCCTTATCACAGCCGTCCTTCTCCTTCTTTCACTGCCCGTCCTCGCCGCCGGCATCACCATGCTACTTACAGACC

HAP88 ATCCGCGCGGAA-CTCAGCCAGCCCGGCGCACTACTTGGAGATGACCAGATTTATAACGTAATTGTTACTGCTCACGCCTTCGTAATGATTTTCTTTATAGTAATACCAATTATGATTGGAGGGTTTGGGAACTGACTGATTCCTCTAATGATTGGGGCCCCCGACATGGCCTTCCCACGAATGAATAACATGAGCTTCTGGCTTTTGCCCCCATCATTCCTGCTCCTCCTCGCCTCTTCAGGCGTTGAAGCTGGGGCTGGGACAGGGTGGACCGTCTACCCCCCACTGGCAGGCAATCTAGCCCACGCAGGCGCATCTGTCGACCTAACAATCTTTTCTCTCCACCTCGCCGGGATCTCTTCCATTCTCGGGGCCATTAACTTTATCACCACAATCCTAAACATGAAACCGCCCGCTGTCTCGCAGTATCAAACGCCCCTCTTCGTGTGGGCCGTCCTTATCACAGCCGTCCTTCTCCTTCTTTCACTGCCCGTCCTCGCGGCCGGCATCACCATGCTACTTACAGACC
