# Supplementary material for: Distribution and population structure in the naked goby Gobiosoma bosc (Perciformes: Gobiidae) along a salinity gradient in two western Atlantic estuaries
Source: PeerJ. 2018 Aug 7;6:e5380. doi: 10.7717/peerj.5380 (PMC6086083; doi:10.7717/peerj.5380)
Supplement: Table S2 [file peerj-06-5380-s002.pdf]

	GSC	MLC	NCL	WRC	FSL	CQC	MTP	POC
GSC	0							
MLC	0.00927	0						
NCL	-0.00334	-0.00032	0					
WRC	-0.00797	-0.03473	-0.012	0				
FSL	-0.0161	-0.0167	-0.02228	0.02778	0			
CQC	0.02474	0.07242	0.04755	0.067	0.01743	0		
MTP	0.00506	-0.00059	-0.00179	-0.00403	-0.01087	0.06667	0	
POC	-0.00875	0.1062	0.05469	0.21127	0.07508	-0.1131	0.11432	0
CDI	0.01005	0.00396	-0.01223	0.00587	-0.02402	0.03491	0.01079	-0.00338
HPC	0.05418	0.18767	0.13095	0.25396	0.15569	-0.035	0.19251	-0.08367
GULF	0.62101	0.72604	0.69638	0.89472	0.83768	0.47105	0.75498	0.66667
GERM	0.87808	0.85445	0.85855	0.84423	0.74778	0.78472	0.84228	0.64052

CDI	HPC	GULF	GERM
0			
0.07985	0		
0.57618	0.65692	0	
0.81896	0.74536	0.63704	0
