# Supplementary material for: Distribution and population structure in the naked goby Gobiosoma bosc (Perciformes: Gobiidae) along a salinity gradient in two western Atlantic estuaries
Source: PeerJ. 2018 Aug 7;6:e5380. doi: 10.7717/peerj.5380 (PMC6086083; doi:10.7717/peerj.5380)
Supplement: Supplemental Information 1 — Sequence data for North Carolina Populations, Atlantic and Gulf of Mexico Populations (Mila et al., 2017), and German Populations (Van Tassell et al., 2015) of G. bosc [file peerj-06-5380-s004.docx]

***Sequence Information***:

>HAP1_GERM

ATCCGCGCGGAA-CTCAGCCAGCCCGGCGCACTACTTGGGGACGACCAGATTTATAACGTAATTGTCACTGCCCACGCCTTTGTAATGATTTTCTTTATAGTAATACCAATTATGATTGGGGGCTTTGGGAACTGGCTAATTCCCCTAATGATTGGGGCCCCCGACATGGCCTTCCCCCGAATGAACAACATGAGCTTCTGGCTTTTGCCCCCCTCATTCCTGCTCCTTCTCGCCTCTTCGGGCGTTGAGGCTGGGGCTGGGACAGGGTGGACTGTCTACCCCCCATTGGCAGGAAACCTGGCCCACGCAGGCGCATCTGTCGACCTAACAATCTTTTCTCTCCACCTCGCCGGGATCTCTTCCATTCTTGGCGCCATTAACTTTATCACCACAATCCTAAACATGAAGCCACCCGCTATCTCGCAGTATCAAACGCCCCTCTTCGTATGGGCCGTTCTTATTACAGCCGTTCTCCTCCTTCTCTCACTGCCCGTCCTCGCCGCCGGCATCACCATGCTACTTACAGACC

>HAP2_GERM_NY

ATCCGCGCGGAA-CTCAGCCAGCCCGGCGCACTACTTGGGGACGACCAGATTTATAACGTAATTGTCACTGCCCACGCCTTTGTAATGATTTTCTTTATAGTAATACCAATTATGATTGGGGGCTTTGGGAACTGACTAATTCCCCTAATGATTGGGGCCCCCGACATGGCCTTCCCCCGAATGAACAACATGAGCTTCTGGCTTTTGCCCCCCTCATTCCTGCTCCTTCTCGCCTCTTCGGGCGTTGAGGCTGGGGCTGGGACAGGGTGGACTGTCTACCCCCCATTGGCAGGAAACCTGGCCCACGCAGGCGCATCTGTCGACCTAACAATCTTTTCTCTCCACCTCGCCGGGATCTCTTCCATTCTTGGCGCCATTAACTTTATCACCACAATCCTAAACATGAAGCCACCCGCTATCTCGCAGTATCAAACGCCCCTCTTCGTATGGGCCGTTCTTATTACAGCCGTTCTCCTCCTTCTCTCACTGCCCGTCCTCGCCGCCGGCATCACCATGCTACTTACAGACC

>HAP3_GERM

ATCCGCGCGGAA-CTCAGCCAGCCCGGCGCACTACTTGGGGACGACCAGATTTATAACGTAATTGTCACTGCCCACGCCTTTGTAATGATTTTCTTTATAGTAATACCAATTATGATTGGGGGCTTTGGGAACTGACTAATTCCCCTAATGATTGGGGCCCCCGACATGGCCTTCCCCCGAATGAACAACATGAGCTTCTGGCTTTTGCCCCCCTCATTCCTGCTCCTTCTCGCCTCTTCGGGCGTTGAGGCTGGGGCTGGGACAGGGTGGACTGTCTACCCACCATTGGCAGGAAACCTGGCCCACGCAGGCGCATCTGTCGACCTAACAATCTTTTCTCTCCACCTCGCCGGGATCTCTTCCATTCTTGGCGCCATTAACTTTATCACCACAATCCTAAACATGAAGCCACCCGCTATCTCGCAGTATCAAACGCCCCTCTTCGTATGGGCCGTTCTTATTACAGCCGTTCTCCTCCTTCTCTCACTGCCCGTCCTCGCCGCCGGCATCACCATGCTACTTACAGACC

>HAP4_GERM_FL

ATCCGCGCGGAGGCTCAGCCAGCCCGGCG-ACTACTTGGGGACGACCAGATTTATAACGTAATTGTCACTGCCCACGCCTTTGTAATGATTTTCTTTATAGTAATACCAATTATGATTGGGGGCTTTGGGAACTGACTAATTCCCCTAATGATTGGGGCCCCCGACATGGCCTTCCCCCGAATGAACAACATGAGCTTCTGGCTTTTGCCCCCCTCATTCCTGCTCCTTCTCGCCTCTTCGGGCGTTGAGGCTGGGGCTGGGACAGGGTGGACTGTCTACCCACCATTGGCAGGAAACCTGGCCCACGCAGGCGCATCTGTCGACCTAACAATCTTTTCTCTCCACCTCGCCGGGATCTCTTCCATTCTTGGCGCCATTAACTTTATCACCACAATCCTAAACATGAAGCCACCCGCTATCTCGCAGTATCAAACGCCCCTCTTCGTATGGGCCGTTCTTATTACAGCCGTTCTCCTCCTTCTCTCACTGCCCGTCCTCGCCGCCGGCATCACCATGCTACTTACAGACC

>HAP5_NCUBIQUITOUS

ATCCGCGCGGAGGCTCAGCCAGCCCGGCG-ACTACTTGGGGACGACCAGATTTATAACGTAATTGTCACTGCCCACGCCTTTGTAATGATTTTCTTTATAGTAATACCAATTATGATTGGGGGCTTTGGGAACTGACTAATTCCCCTAATGATTGGGGCCCCCGACATGGCCTTCCCCCGAATGAACAACATGAGCTTCTGGCTTTTGCCCCCCTCATTCCTGCTCCTTCTCGCCTCTTCGGGCGTTGAGGCTGGGGCTGGGACAGGGTGGACTGTCTACCCACCATTGGCAGGAAACCTGGCCCACGCAGGCGCATCTGTCGACCTAACAATCTTTTCTCTCCACCTCGCCGGGATCTCTTCCATTCTTGGCGCCATTAACTTTATCACCACAATCCTAAACATGAAGCCACCCGCTATCTCGCAGTATCAAACGCCCCTCTTCGTATGGGCCGTTCTTATCACAGCCGTTCTCCTCCTTCTCTCACTGCCCGTCCTCGCCGCCGGCATCACCATGCTACTTACAGACC

>HAP6_NC_NCL

ATCCGCGCGGAGGCTCAGCCAGCCCGGCG-ACTACTTGGGGACGACCAGATTTATAACGTAATTGTCACTGCCCACGCCTTTGTAATGATTTTCTTTATAGTAATACCAATTATGATTGGGGGCTTTGGAAACTGACTAATTCCCCTAATGATTGGGGCCCCCGACATGGCCTTCCCCCGAATGAACAACATGAGCTTCTGGCTTTTGCCCCCCTCATTCCTGCTCCTTCTCGCCTCTTCGGGCGTTGAGGCTGGGGCTGGGACAGGGTGGACTGTCTACCCACCATTGGCAGGAAACCTGGCCCACGCAGGCGCATCTGTCGACCTAACAATCTTTTCTCTCCACCTCGCCGGGATCTCTTCCATTCTTGGCGCCATTAACTTTATCACCACAATCCTAAACATGAAGCCACCCGCTATCTCGCAGTATCAAACGCCCCTCTTCGTATGGGCCGTTCTTATCACAGCCGTTCTCCTCCTTCTCTCACTGCCCGTCCTCGCCGCCGGCATCACCATGCTACTTACAGACC

>HAP7_NC_CQC

ATCCGCGCGGAGGCTCAGCCAGCCCGGCG-ACTACTTGGGGACGACCAGATTTATAACGTAATTGTCACTGCCCACGCCTTTGTAATGATTTTCTTTATAGTAATACCAATTATGATTGGGGGCTTTGGGAACTGACTAATTCCCCTAATGATTGGAGCCCCCGACATGGCCTTCCCCCGAATGAACAACATGAGCTTCTGGCTTTTGCCCCCCTCATTCCTGCTCCTTCTCGCCTCTTCGGGCGTTGAGGCTGGGGCTGGGACAGGGTGGACTGTCTACCCACCATTGGCAGGAAACCTGGCCCACGCAGGCGCATCTGTCGACCTAACAATCTTTTCTCTCCACCTCGCCGGGATCTCTTCCATTCTTGGCGCCATTAACTTTATCACCACAATCCTAAACATGAAGCCACCCGCTATCTCGCAGTATCAAACGCCCCTCTTCGTATGGGCCGTTCTTATCACAGCCGTTCTCCTCCTTCTCTCACTGCCCGTCCTCGCCGCCGGCATCACCATGCTACTTACAGACC

>HAP8_MTP

ATCCGCGCGGAGGCTCAGCCAGCCCGGCG-ACTACTTGGGGACGACCAGATTTATAACGTAATTGTCACTGCCCACGCCTTTGTAATGATTTTCTTTATAGTAATACCAATTATGATTGGGGGCTTTGGGAACTGACTAATTCCCCTAATGATTGGGGCCCCCGACATGGCCTTCCCCCGAATGAACAACATGAGCTTCTGGCTTTTGCCCCCCTCATTCCTGCTCCTTCTCGCCTCTTCGGGCGTTGAGGCTGGGGCTGGGACAGGGTGGACTGTCTACCCACCATTGGCAGGAAACCTGGCCCACGCAGGCGCATCTGTCGACCTAACAATCTTTTCTCTCCACCTCGCCGGGATCTCTTCCATTCTTGGCGCCATTAACTTTATCACCACAATCCTAAACATGAAGCCACCCGCTATCTCGCAGTATCAAACGCCCCTCTTCGTATGGGCCGTTCTTATCACAGCCGTTCTCCTCCTTCTCTCACTGCCCGTCCTCGCCGCCGGCATCACCATGCTACTTAC-GACC

>HAP9_GSC

ATCCGCGCGGAGGCTCAGCCAGCCCGGCG-ACTACTTGGGGACGACCAGATTTATAACGTAATTGTCACTGCCCACGCCTTTGTAATGATTTTCTTTATAGTAATACCAATTATGATTGGGGGCTTTGGGAACTGACTAATTCCCCTAATGATTGGGGCCCCCGACATGGCCTTCCCCCGAATGAACAACATGAGCTTCTGGCTTTTGCCCCCCTCATTCCTGCTCCTTCTCGCCTCTTCGGGCGTTGAGGCTGGGGCTGGGACAGGGTGGACTGTCTACCCACCATTGGCAGGAAACCTGGCCCACGCAGGCGCATCTGTCGACCTAACAATCTTTTCCCTCCACCTCGCCGGGATCTCTTCCATTCTTGGCGCCATTAACTTTATCACCACAATCCTAAACATGAAGCCACCCGCTATCTCGCAGTATCAAACGCCCCTCTTCGTATGGGCCGTTCTTATCACAGCCGTTCTCCTCCTTCTCTCACTGCCCGTCCTCGCCGCCGGCATCACCATGCTACTTACAGACC

>HAP10_GSC

ATCCGCGCGGAGGCTCAGCCAGCCCGGCG-ACTACTTGGGGACGACCAGATTTATAACGTAATTGTCACTGCCCACGCCTTTGTAATGATTTTCTTTATAGTAATACCAATTATGATTGGGGGCTTTGGGAACTGACTAATTCCCCTAATGATTGGGGCCCCCGACATGGCCTTCCCCCGAATGAACAACATGAGCTTCTGGCTTTTGCCCCCCTCATTCTTGCTCCTTCTCGCCTCTTCGGGCGTTGAGGCTGGGGCTGGGACAGGGTGGACTGTCTACCCACCATTGGCAGGAAACCTGGCCCACGCAGGCGCATCTGTCGACCTAACAATCTTTTCTCTCCACCTCGCCGGGATCTCTTCCATTCTTGGCGCCATTAACTTTATCACCACAATCCTAAACATGAAGCCACCCGCTATCTCGCAGTATCAAACGCCCCTCTTCGTATGGGCCGTTCTTATCACAGCCGTTCTCCTCCTTCTCTCACTGGCCGTCCTCGCCGCCGGCATCACCATGCTACTTACAGACC

>HAP11_FSL

ATCCGCGCGGAGGCTCAGCCAGCCCGGCG-ACTACTTGGGGACGACCAGATTTATAACGTAATTGTCACTGCCCACGCCTTTGTAATGATTTTCTTTATAGTAATACCAATTATGATTGGGGGCTTTGGGAACTGACTAATTCCCCTAATGATTGGGGCCCCCGACATGGCCTTCCCCCGAATGAACAACATGAGCTTCTGGCTTTTGCCCCCCTCATTCCTGCTCCTTCTCGCCTCTTCGGGCGTTGAGGCTGGGGCTGGGACAGGGTGGACTGTCTACCCACCATTGGCAGGAAACCTGGCCCACGCTGGCGCATCTGTCGACCTAACAATCTTTTCTCTCCACCTCGCCGGGATCTCTTCCATTCTTGGCGCCATTAACTTTATCACCACAATCCTAAACATGAAGCCACCCGCTATCTCGCAGTATCAAACGCCCCTCTTCGTATGGGCCGTTCTTATCACAGCCGTTCTCCTCCTTCTCTCACTGCCCGTCCTCGCCGCCGGCATCACCATGCTACTTACAGACC

>HAP12_MTP

ATCCGCGCGGAGGCTCAGCCAGCCCGGCG-ACTACTTGGGGACGACCAGATTTATAACGTAATTGTCACTGCCCACGCCTTTGTAATGATTTTCTTTATAGTAATACCAATTATGATTGGGGGCTTTGGGAACTGACTAATTCCCCTAATGATTGGGGCCCCCGACATGGCCTTCCCCCGAATGAACAACATGAGCTTCTGGCTTTTGCCCCCCTCATTCCTGCTCCTTCTCGCCTCTTCGGGCGTTGAGGCTGGGGCTGGGACAGGGTGGACTGTCTACCCACCATTGGCAGGAAACCTGGCCCACGCAGGCGCATCTGTCGACCTAACAATCTTTTCTCTCCACCTCGCCGGGATCTCTTCCATTCTTGGCGCCATTAACTTTATCACCACAATCCTAAACATGAAGCCACCCGCTATCTCGCAGTATCAAACACCCCTCTTCGTATGGGCCGTTCTTATCACAGCCGTTCTCCTCCTTCTCTCACTGCCCGTCCTCGCCGCCGGCATCACCATGCTACTTACAGACC

>HAP13_GSC

ATCCGCGCGGAGGCTCAGCCAGCCCGGCG-ACTACTTGGGGACGACCAGATTTATAACGTAATTGTCACTGCCCACGCCTTTGTAATGATTTTCTTTATAGTAATACCAATTATGATTGGGGGCTTTGGGAACTGACTAATTCCCCTAATGATCGGGGCCCCCGACATGGCCTTCCCCCGAATGAACAACATGAGCTTCTGGCTTTTGCCCCCCTCATTCCTGCTCCTTCTCGCCTCTTCGGGCGTTGAGGCTGGGGCTGGGACAGGGTGGACTGTCTACCCACCATTGGCAGGAAACCTGGCCCACGCAGGCGCATCTGTCGACCTAACAATCTTTTCTCTCCACCTCGCCGGGATCTCTTCCATTCTTGGCGCCATTAACTTTATCACCACAATCCTAAACATGAAGCCACCCGCTATCTCGCAGTATCAAACGCCCCTCTTCGTATGGGCCGTTCTTATCACAGCCGTTCTCCTCCTTCTCTCACTGCCCGTCCTCGCCGCCGGCATCACCATGCTACTTACAGACC

>HAP14_NCL

ATCCGCGCGGAGGCTCAGCCAGCCCGGCG-ACTACTTGGGGACGACCAGATTTATAACGTAATTGTCACTGCCCACGCCTTTGTAATGATTTTCTTTATAGTAATACCAATTATGATTGGGGGCTTTGGGAACTGACTAATTCCCCTAATGATTGGGGCCCCCGACATGGCCTTCCCCCGAATGAACAACATGAGTTTCTGGCTTTTGCCCCCCTCATTCCTGCTCCTTCTCGCCTCTTCGGGCGTTGAGGCTGGGGCTGGGACAGGGTGGACTGTCTACCCACCATTGGCAGGAAACCTGGCCCACGCAGGCGCATCTGTCGACCTAACAATCTTTTCTCTCCACCTCGCCGGGATCTCTTCCATTCTTGGCGCCATTAACTTTATCACCACAATCCTAAACATGAAGCCACCCGCTATCTCGCAGTATCAAACGCCCCTCTTCGTATGGGCCGTTCTTATCACAGCCGTTCTCCTCCTTCTCTCACTGCCCGTCCTCGCCGCCGGCATCACCATGCTACTTACAGACC

>HAP15_MTP

ATCCGCGCGGAGGCTCAGCCAGCCCGGCG-ACTACTTGGGGACGACCAGATTTATAACGTAATTGTCACTGCCCACGCCTTTGTAATGATTTTCTTTATAGTAATACCAATTATGATTGGGGGCTTTGGGAACTGACTAATTCCCCTAATGATTGGGGCCCCCGACATAGCCTTCCCCCGAATGAACAACATGAGCTTCTGGCTTTTGCCCCCCTCATTCCTGCTCCTTCTCGCCTCTTCGGGCGTTGAGGCTGGGGCTGGGACAGGGTGGACTGTCTACCCACCATTGGCAGGAAACCTGGCCCACGCAGGCGCATCTGTCGACCTAACAATCTTTTCTCTCCACCTCGCCGGGATCTCTTCCATTCTTGGCGCCATTAACTTTATCACCACAATCCTAAACATGAAGCCACCCGCTATCTCGCAGTATCAAACGCCCCTCTTCGTATGGGCCGTTCTTATCACAGCCGTTCTCCTCCTTCTCTCACTGCCCGTCCTCGCCGCCGGCATCACCATGCTACTTACAGACC

>HAP16_CDI

ATCCGCGCGGAGGCTCAGCCAGCCCGGCG-ACTACTTGGGGACGACCAGATTTATAACGTAATTGTCACTGCCCACGCCTTTGTAATGATTTTCTTTATAGTAATACCAATTATGATTGGGGGCTTTGGGAACTGACTAATTCCCCTAATGATTGGGGCCCCCGACATGGCCTTCCCCCGAATGAACAACATGAGCTTCTGGCTTTTGCCCCCCTCATTCCTGCTTCTTCTCGCCTCTTCGGGCGTTGAGGCTGGGGCTGGGACAGGGTGGACTGTCTACCCACCATTGGCAGGAAACCTGGCCCACGCAGGCGCATCTGTCGACCTAACAATCTTTTCTCTCCACCTCGCCGGGATCTCTTCCATTCTTGGCGCCATTAACTTTATCACCACAATCCTAAACATGAAGCCACCCGCTATCTCGCAGTATCAAACGCCCCTCTTCGTATGGGCCGTTCTTATCACAGCCGTTCTCCTCCTTCTCTCACTGCCCGTCCTCGCCGCCGGCATCACCATGCTACTTACAGACC

>HAP17_CQC

ATCCGCGCGGAGGCTCAGCCAGCCCGGCG-ACTACTTGGGGACGACCAGATTTATAACGTAATTGTCACTGCCCACGCCTTTGTAATGATTTTCTTTATAGTAATACCAATTATGATTGGGGGCTTTGGGAACTGACTAATTCCCCTAATGATTGGGGCCCCCGACATGGCCTTCCCCCGAATGAACAACATGAGCTTCTGGCTTTTGCCCCCCTCATTCCTGCTCCTTCTCGCCTCTTCGGGCGTTGAGGCTGGGGCTGGGACAGGGTGGACTGTCTACCCACCATTGGCAGGAAACCTGGCCCACGCAGGCGCATCTGTCGACCTAACAATCTTTTCTCTCCACCTCGCCGGGATCTCTTCCATTCTTGGCGCCATTAACTTTATCACCACAATCCTAAACATGAAGCCACCCGCTATCTCGCAGTATCAAACGCCCCTCTTCGTATGGGCCGTTCTTATCACAGCCGTTCTCCTCCTTCTCTCACTGCCCGTCCTCGCCGCCGGCATCACCATGCTACTCACAGACC

>HAP18_MLC

ATCCGCGCGGAGGCTCAGCCAGCCCGGCG-ACTACTTGGGGACGACCAGATTTATAACGTAATTGTCACTGCCCACGCCTTTGTAATGATTTTCTTTATAGTAATACCAATTATGATTGGTGGCTTTGGGAACTGACTAATTCCCCTAATGATTGGGGCCCCCGACATGGCCTTCCCCCGAATGAACAACATGAGCTTCTGGCTTTTGCCCCCCTCATTCCTGCTCCTTCTCGCCTCTTCGGGCGTTGAGGCTGGGGCTGGGACAGGGTGGACTGTCTACCCACCATTGGCAGGAAACCTGGCCCACGCAGGCGCATCTGTCGACCTAACAATCTTTTCTCTCCACCTCGCCGGGATCTCTTCCATTCTTGGCGCCATTAACTTTATCACCACAATCCTAAACATGAAGCCACCCGCTATCTCGCAGTATCAAACGCCCCTCTTCGTATGGGCCGTTCTTATCACAGCCGTTCTCCTCCTTCTCTCACTGCCCGTCCTCGCCGCCGGCATCACCATGCTACTTACAGACC

>HAP19_MLC_GSC_WRC

ATCCGCGCGGAGGCTCAGCCAGCCCGGCG-ACTACTTGGGGACGACCAGATTTATAACGTAATTGTCACTGCCCACGCCTTTGTAATGATTTTCTTTATAGTAATACCAATTATGATTGGGGGCTTTGGGAACTGACTAATTCCCCTAATGATTGGGGCCCCCGACATGGCCTTCCCCCGAATGAACAACATGAGCTTCTGGCTTTTACCCCCCTCATTCCTGCTCCTTCTCGCCTCTTCGGGCGTTGAGGCTGGGGCTGGGACAGGGTGGACTGTCTACCCACCATTGGCAGGAAACCTGGCCCACGCAGGCGCATCTGTCGACCTAACAATCTTTTCTCTCCACCTCGCCGGGATCTCTTCCATTCTTGGCGCCATTAACTTTATCACCACAATCCTAAACATGAAGCCACCCGCTATCTCGCAGTATCAAACGCCCCTCTTCGTATGGGCCGTTCTTATCACAGCCGTTCTCCTCCTTCTCTCACTGCCCGTCCTCGCCGCCGGCATCACCATGCTACTTACAGACC

>HAP20_CQC

ATCCGCGCGGAGGCTCAGCCAGCCCGGCG-ACTACTTGGGGACGACCAGATTTATAACGTAATTGTCACTGCCCACGCCTTTGTAATGATTTTCTTTATAGTAATACCAATTATGATTGGGGGCTTTGGGAACTGACTAATTCCCCTAATGATTGGGGCCCCCGACATGGCCTTCCCCCGAATGAACAACATGAGCTTCTGGCTTTTGCCCCCCTCATTCCTGCTCCTTCTCGCCTCTTCGGGCGTTGAGGCTGGGGCTGGGACAGGGTGGACTGTCTACCCACCATTGGCAGGAAACCTGGCCCACGCAGGCGCATCTGTCGACCTAACAATCTTTTCTCTCCACCTCGCCGGGATTTCTTCCATTCTTGGCGCCATTAACTTTATCACCACAATCCTAAACATGAAGCCACCCGCTATCTCGCAGTATCAAACGCCCCTCTTCGTATGGGCCGTTCTTATCACAGCCGTTCTCCTCCTTCTCTCACTGCCCGTCCTCGCCGCCGGCATCACCATGCTACTTACAGACC

>HAP21_GSC_MTP

ATCCGCGCGGAGGCTCAGCCAGCCCGGCG-ACTACTTGGGGACGACCAGATTTATAACGTAATTGTCACTGCCCACGCCTTTGTAATGATTTTCTTTATAGTAATACCAATTATGATTGGGGGCTTTGGGAACTGACTAATTCCCCTAATGATTGGGGCCCCCGACATGGCCTTCCCCCGAATGAACAACATGAGCTTCTGGCTTTTGCCCCCCTCATTCCTGCTCCTTCTCGCCTCTTCGGGCGTTGAGGCTGGGGCTGGGACAGGGTGGACTGTCTACCCACCATTGGCAGGAAACCTAGCCCACGCAGGCGCATCTGTCGACCTAACAATCTTTTCTCTCCACCTCGCCGGGATCTCTTCCATTCTTGGCGCCATTAACTTTATCACCACAATCCTAAACATGAAGCCACCCGCTATCTCGCAGTATCAAACGCCCCTCTTCGTATGGGCCGTTCTTATCACAGCCGTTCTCCTCCTTCTCTCACTGCCCGTCCTCGCCGCCGGCATCACCATGCTACTTACAGACC

>HAP22_MLC_CDI

ATCCGCGCGGAGGCTCAGCCAGCCCGGCG-ACTACTTGGGGATGACCAGATTTATAACGTAATTGTCACTGCCCACGCCTTTGTAATGATTTTCTTTATAGTAATACCAATTATGATTGGGGGCTTTGGGAACTGACTAATTCCCCTAATGATTGGGGCCCCCGACATGGCCTTCCCCCGAATGAACAACATGAGCTTCTGGCTTTTGCCCCCCTCATTCCTGCTCCTTCTCGCCTCTTCGGGCGTTGAGGCTGGGGCTGGGACAGGGTGGACTGTCTACCCACCATTGGCAGGAAACCTGGCCCACGCAGGCGCATCTGTCGACCTAACAATCTTTTCTCTCCACCTCGCCGGGATCTCTTCCATTCTTGGCGCCATTAACTTTATCACCACAATCCTAAACATGAAGCCACCCGCTATCTCGCAGTATCAAACGCCCCTCTTCGTATGGGCCGTTCTTATCACAGCCGTTCTCCTCCTTCTCTCACTGCCCGTCCTCGCCGCCGGCATCACCATGCTACTTACAGACC

>HAP23_NCL

ATCCGCGCGGAGGCTCAGCCAGCCCGGCG-ACTACTTGGGGACGACCAGATTTATAACGTAATTGTCACTGCCCACGCCTTTGTAATGATTTTCTTTATAGTAATACCAATTATGATTGGGGGCTTTGGGAACTGACTAATTCCCCTAATGATTGGGGCCCCCGACATGGCCTTCCCCCGAATGAACAACATGAGCTTCTGGCTTTTGCCCCCCTCATTCCTGCTCCTTCTCGCCTCTTCGGGCGTTGAGGCTGGGGCTGGGACAGGGTGGACTGTCTACCCACCATTGGCAGGAAACCTGGCCCACGCAGGCGCATCTGTCGACCTAACAATCTTTTCTCTCCACCTCGCCGGGATCTCTTCCATTCTTGGCGCCATTAACTTTATCACCACAATCCTAAACATGAAGCCGCCCGCTATCTCGCAGTATCAAACGCCCCTCTTCGTATGGGCCGTTCTTATCACAGCCGTTCTCCTCCTTCTCTCACTGCCCGTCCTCGCCGCCGGCATCACCATGCTACTTACAGACC

>HAP24_MLC

ATCCGCGCGGAGGCTCAGCCAGCCCGGCG-ACTACTTGGGGACGACCAGATTTATAACGTAATTGTCACTGCCCACGCCTTTGTAATGATTTTCTTTATAGTAATACCAATTATGATTGGGGGCTTTGGGAACTGACTAATTCCCCTAATGATTGGGGCCCCCGACATGGCCTTCCCCCGAATGAACAACATGAGCTTCTGGCTTTTGCCCCCCTCATTCCTGCTCCTTCTCGCCTCTTCGGGCGTTGAGGCTGGGGCTGGGACAGGGTGGACTGTCTACCCACCATTGGCAGGAAACCTGGCCCACGCAGGCGCATCTGTCGACCTAACAATCTTTTCTCTCCACCTCGCCGGGATCTCTTCCATTCTTGGCGCCATTAACTTTATCACCACAATCCTAAACATGAAGCCACCCGCTATCTCGCAGTATCAAACGCCCCTCTTCGTATGGGCCGTTCTTATCACAGCCGTCCTCCTCCTTCTCTCACTGCCCGTCCTCGCCGCCGGCATCACCATGCTACTTACAGACC

>HAP25_CDI

ATCCGCGCGGAGGCTCAGCCAGCCCGGCG-ACTACTTGGGGACGACCAGATTTATAACGTAATTGTCACTGCCCACGCCTTTGTAATGATTTTCTTTATAGTAATACCAATTATGATTGGGGGCTTTGGGAACTGACTAATTCCCCTAATGATTGGGGCCCCCGACATGGCCTTCCCCCGAATGAACAACATGAGCTTCTGGCTTTTGCCCCCCTCATTCCTGCTCCTTCTCGCCTCTTCGGGCGTTGAAGCTGGGGCTGGGACAGGGTGGACTGTCTACCCACCATTGGCAGGAAACCTGGCCCACGCAGGCGCATCTGTCGACCTAACAATCTTTTCTCTCCACCTCGCCGGGATCTCTTCCATTCTTGGCGCCATTAACTTTATCACCACAATCCTAAACATGAAGCCACCCGCTATCTCGCAGTATCAAACGCCCCTCTTCGTATGGGCCGTTCTTATCACAGCCGTTCTCCTCCTTCTCTCACTGCCCGTCCTCGCCGCTGGCATCACCATGCTACTTACAGACC

>HAP26_CDI_NCL

ATCCGCGCGGAGGCTCAGCCAGCCCGGCG-ACTACTTGGGGACGACCAGATTTATAACGTAATTGTCACTGCCCACGCCTTTGTAATGATTTTCTTTATAGTAATACCAATTATGATTGGGGGCTTTGGGAACTGACTAATTCCCCTAATGATTGGGGCCCCCGACATGGCCTTCCCCCGAATGAACAACATGAGCTTCTGGCTTTTGCCCCCCTCATTCCTGCTCCTTCTCGCCTCTTCGGGCGTTGAAGCTGGGGCTGGGACAGGGTGGACTGTCTACCCACCATTGGCAGGAAACCTGGCCCACGCAGGCGCATCTGTCGACCTAACAATCTTTTCTCTCCACCTCGCCGGGATCTCTTCCATTCTTGGCGCCATTAACTTTATCACCACAATCCTAAACATGAAGCCACCCGCTATCTCGCAGTATCAAACGCCCCTCTTCGTATGGGCCGTTCTTATCACAGCCGTTCTCCTCCTTCTCTCACTGCCCGTCCTCGCCGCCGGCATCACCATGCTACTTACAGACC

>HAP27_POC_NCL_HPC_CQC_GSC

ATCCGCGCGGAGGCTCAGCCAGCCCGGCG-ACTACTTGGGGACGACCAGATTTATAACGTAATTGTCACTGCCCACGCCTTTGTAATGATTTTCTTTATAGTAATACCAATTATGATTGGGGGCTTTGGGAACTGACTAATTCCCCTAATGATTGGGGCCCCCGACATGGCCTTCCCCCGAATGAACAACATGAGCTTCTGGCTTTTGCCCCCCTCATTCCTGCTCCTTCTCGCCTCTTCGGGCGTTGAGGCTGGGGCTGGGACAGGGTGGACTGTCTACCCACCATTGGCAGGAAACCTGGCCCACGCAGGCGCATCTGTCGACCTAACAATCTTTTCTCTCCACCTCGCCGGGATCTCTTCCATTCTTGGCGCCATTAACTTTATCACCACAATCCTAAACATGAAGCCACCCGCTATCTCGCAGTATCAAACGCCCCTCTTCGTATGGGCCGTTCTTATCACAGCCGTTCTCCTCCTTCCCTCACTGCCCGTCCTCGCCGCCGGCATCACCATGCTACTTACAGACC

>HAP28_CDI

ATCCGCGCGGAGGCTCAGCCAGCCCGGCG-ACTACTTGGGGACGACCAGATTTATAACGTAATTGTCACTGCCCACGCCTTTGTAATGATTTTCTTTATAGTAATACCAATTATGATTGGGGGCTTTGGGAACTGACTAATTCCCCTAATGATTGGGGCCCCCGACATGGCCTTCCCCCGAATGAACAACATGAGCTTCTGGCTTTTGCCCCCCTCATTCCTGCTCCTTCTCGCCTCTTCGGGCGTTGAGGCTGGGGCTGGGACAGGGTGGACTGTCTACCCACCATTGGCAGGAAACCTGGCCCACGCAGGCGCATCCGTCGACCTAACAATCTTTTCTCTCCACCTCGCCGGGATCTCTTCCATTCTTGGCGCCATTAACTTTATCACCACAATCCTAAACATGAAGCCACCCGCTATCTCGCAGTATCAAACGCCCCTCTTCGTATGGGCCGTTCTTATCACAGCCGTTCTCCTCCTTCCCTCACTGCCCGTCCTCGCCGCCGGCATCACCATGCTACTTACAGACC

>HAP29_GSC

ATCCGCGCGGAGGCTCAGCCAGCCCGGCG-ACTACTTGGGGACGACCAGATTTATAACGTAATTGTCACTGCCCACGCCTTTGTAATGATTTTCTTTATAGTAATACCAATTATGATTGGGGGCTTTGGGAACTGACTAATTCCCCTAATGATTGGGGCCCCCGACATGGCCTTCCCCCGAATGAACAACATGAGCTTCTGGCTTTTGCCCCCCTCATTCCTGCTCCTTCTCGCCTCTTCGGGCGTTGAGGCTGGGGCTGGGACAGGGTGGACTGTCTACCCACCATTGGCAGGAAACCTGGCCCACGCAGGCGCATCTGTCGACCTAACAATCTTTTCTCTCCACCTCGCCGGGATCTCTTCCATTCTTGGCGCCATTAACTTTATCACCACAATCCTAAACATGAAACCACCCGCTATCTCGCAGTATCAAACGCCCCTCTTCGTATGGGCCGTTCTTATCACAGCCGTTCTCCTCCTTCCCTCACTGCCCGTCCTCGCCGCCGGCATCACCATGCTACTTACAGACC

>HAP30_HPC

ATCCGCGCGGAGGCTCAGCCAGCCCGGCG-ACTACTTGGGGACGACCAGATTTATAACGTAATTGTCACTGCCCACGCCTTTGTAATGATTTTCTTTATAGTAATACCAATTATGATTGGGGGCTTTGGGAACTGACTAATTCCCCTAATGATTGGGGCCCCCGACATGGCCTTCCCCCGAATGAACAACATGAGCTTCTGGCTTTTGCCCCCCTCATTCCTGCTCCTTCTCGCCTCTTCGGGCGTTGAGGCTGGGGCCGGGACAGGGTGGACTGTCTACCCACCATTGGCAGGAAACCTGGCCCACGCAGGCGCATCTGTCGACCTAACAATCTTTTCTCTCCACCTCGCCGGGATCTCTTCCATTCTTGGCGCCATTAACTTTATCACCACAATCCTAAACATGAAGCCACCCGCTATCTCGCAGTATCAAACGCCCCTCTTCGTATGGGCCGTTCTTATCACAGCCGTTCTCCTCCTTCCCTCACTGCCCGTCCTCGCCGCCGGCATCACCATGCTACTTACAGACC

>HAP31_CQC

ATCCGCGCGGAGGCTCAGCCAGCCCGGCG-ACTACTTGGGGACGACCAGATTTATAACGTAATTGTCACTGCCCACGCCTTTGTAATGATTTTCTTTATAGTAATACCAATTATGATTGGGGGTTTTGGGAACTGACTAATTCCCCTAATGATTGGGGCCCCCGACATGGCCTTCCCCCGAATGAACAACATGAGCTTCTGACTTTTGCCCCCCTCATTCCTGCTCCTTCTCGCCTCTTCGGGCGTTGAGGCTGGGGCTGGGACAGGGTGGACTGTCTACCCACCATTGGCAGGAAACCTGGCCCACGCAGGCGCATCTGTCGACCTAACAATCTTTTCTCTCCACCTCGCCGGGATCTCTTCCATTCTTGGCGCCATTAACTTTATCACCACAATCCTAAACATGAAGCCACCCGCTATCTCGCAGTATCAAACGCCCCTCTTCGTATGGGCCGTTCTTATCACAGCCGTTCTCCTCCTTCCCTCACTGCCCGTCCTCGCCGCCGGCATCACCATGCTACTTACAGACC

>HAP32_POC

ATCCGCGCGGAGGCTCAGCCAGCCCGGCG-ACTACTTGGGGACGACCAGATTTATAACGTAATTGTCACTGCCCACGCCTTTGTAATGATTTTCTTTATAGTAATACCAATTATGATTGGGGGCTTTGGGAACTGACTAATTCCCCTAATGATTGGGGCCCCCGACATGGCCTTCCCCCGAATGAACAACATGAGCTTCTGACTTTTGCCCCCCTCATTCCTGCTCCTTCTCGCCTCTTCGGGCGTTGAGGCTGGGGCTGGGACAGGGTGGACTGTCTACCCACCATTGGCAGGAAACCTGGCCCACGCAGGCGCATCTGTCGACCTAACAATCTTTTCTCTCCACCTCGCCGGGATCTCTTCCATTCTTGGCGCCATTAACTTTATCACCACAATCCTAAACATGAAGCCACCCGCTATCTCGCAGTATCAAACGCCCCTCTTCGTATGGGCCGTTCTTATCACAGCCGTTCTCCTCCTTCTCTCACTGCCCGTCCTCGCCGCCGGCATCACCATGCTACTTACAGACC

>HAP33_CQC

ATCCGCGCGGAGGCTCAGCCAGCCCGGCG-ACTACTTGGGGACGACCAGATTTATAACGTAATTGTCACTGCCCACGCCTTTGTAATGATTTTCTTTATAGTAATACCAATTATGATTGGGGGCTTTGGGAACTGACTAATTCCCCTAATGATTGGGGCCCCCGACATGGCCTTCCCCCGAATGAACAACATGAGCTTCTGGCTTTTGCCCCCCTCATTCCTGCTCCTTCTCGCCTCTTCGGGCGTTGAGGCTGGGGCTGGGACAGGGTGGACTGTCTACCCCCCATTGGCAGGAAACCTGGCCCACGCAGGCGCATCTGTCGACCTAACAATCTTTTCTCTCCACCTCGCCGGGATCTCTTCCATTCTTGGCGCCATTAACTTTATCACCACAATCCTAAACATGAAGCCACCCGCTATCTCGCAGTATCAAACGCCCCTCTTCGTATGGGCCGTTCTTATCACAGCCGTTCTCCTCCTTCCCTCACTGCCCGTCCTCGCCGCCGGCATCACCATGCTACTTACAGACC

>HAP34_CQC

ATCCGCGCGGAGGCTCAGCCAGCCCGGCG-ACTACTTGGGGACGACCAGATTTATAACGTAATTGTCACTGCCCACGCCTTTGTAATGATTTTCTTTATAGTAATACCAATTATGATTGGGGGCTTTGGGAACTGACTAATTCCCCTAATGATTGGGGCCCCCGACATGGCCTTCCCCCGAATGAACAACATGAGCTTCTGGCTTTTGCCCCCCTCATTCCTGCTCCTTCTCGCCTCTTCGGGCGTTGAGGCTGGGGCTGGGACAGGGTGGACTGTCTACCCCCCATTGGCAGGAAACCTGGCCCACGCAGGCGCATCTGTCGACCTAACAATCTTTTCTCTCCACCTCGCCGGGATCTCTTCCATTCTTGGCGCCATTAACTTTATCACCACAATCCTAAACATGAAGCCACCCGCTATCTCGCAGTATCAAACGCCCCTCTTCGTATGGGCCGTTCTTATCACAGCCGTTCTCCTCCTTCTCTCACTGCCCGTCCTCGCCGCCGGCATCACCATGCTACTTACAGACC

>HAP35_SC

ATCCGCGCGGAG-CTCAGCCAGCCCGGCGCACTACTTGGGGACGACCAGATTTATAACGTAATTGTCACTGCCCACGCCTTTGTAATGATTTTCTTTATAGTAATACCAATTATGATTGGGGGCTTTGGGAACTGACTAATTCCTTTAATGATTGGGGCCCCCGACATGGCCTTCCCCCGAATGAACAACATGAGCTTCTGGCTTTTGCCCCCCTCATTCCTGCTCCTTCTCGCCTCTTCGGGCGTTGAGGCTGGGGCTGGGACAGGGTGGACTGTCTACCCACCATTGGCAGGAAACCTGGCCCACGCAGGCGCATCTGTCGACCTAACAATCTTTTCTCTCCACCTCGCCGGGATCTCTTCCATTCTTGGCGCCATTAACTTTATCACCACAATCCTAAACATGAAGCCACCCGCTATCTCGCAGTATCAAACGCCCCTCTTCGTATGGGCCGTTCTTATCACAGCCGTTCTCCTCCTTCTCTCACTGCCCGTCCTCGCCGCCGGCATCACCATGCTACTTACAGACC

>HAP36_SC

ATCCGCGCGGAG-CTCAGCCAGCCCGGCGCACTACTTGGGGACGACCAGATTTATAACGTAATTGTCACTGCCCACGCCTTTGTAATGATTTTCTTTATAGTAATACCAATTATGATTGGGGGCTTTGGGAACTGACTAATTCCCCTAATGATTGGGGCCCCCGACATGGCCTTCCCCCGAATGAACAACATGAGCTTCTGGCTTTTGCCCCCCTCATTCCTGCTCCTTCTCGCCTCTTCGGGCGTTGAGGCTGGGGCTGGGACAGGGTGGACTGTCTACCCACCATTGGCAGGAAACCTGGCCCACGCAGGCGCATCTGTCGACCTAACAATCTTTTCTCTCCACCTCGCCGGGATCTCTTCCATTCTTGGCGCCATTAACTTTATCACCACAATCCTAAACATGAAGCCACCCACTATCTCGCAGTATCAAACGCCCCTCTTCGTATGGGCCGTTCTTATCACAGCCGTTCTCCTCCTTCTCTCACTGCCCGTCCTCGCCGCCGGCATCACCATGCTACTTACAGACC

>HAP37_SC

ATCCGCGCGGAG-CTCAGCCAGCCCGGCGCACTACTTGGGGACGACCAGATTTATAACGTAATTGTCACTGCCCACGCCTTTGTAATGATTTTCTTTATAGTAATACCAATTATGATTGGGGGCTTTGGGAACTGACTAATTCCCCTAATGATTGGGGCCCCCGACATGGCCTTCCCCCGAATGAACAACATGAGCTTCTGGCTTTTGCCCCCCTCATTCCTGCTCCTCCTCGCCTCTTCGGGCGTTGAGGCTGGGGCTGGGACAGGGTGGACTGTCTACCCACCATTGGCAGGAAACCTGGCCCACGCAGGCGCATCTGTCGACCTAACAATCTTTTCTCTCCACCTCGCCGGGATCTCTTCCATTCTTGGCGCCATTAACTTTATCACCACAATCCTAAACATGAAGCCACCCGCTATCTCGCAGTATCAAACGCCCCTCTTCGTATGGGCCGTTCTTATCACAGCCGTTCTCCTCCTTCTCTCACTGCCCGTCCTCGCCGCCGGCATCACCATGCTACTTACAGACC

>HAP38_JAFL

ATCCGCGCGGAG-CTCAGCCAGCCCGGCGCACTACTTGGGGACGACCAGATTTATAACGTAATTGTCACTGCCCACGCCTTTGTAATGATTTTCTTTATAGTAATACCAATTATGATTGGGGGCTTTGGGAACTGACTAATTCCCCTAATGATTGGGGCCCCCGACATGGCCTTCCCCCGAATGAACAACATGAGCTTCTGGCTTTTGCCCCCCTCATTCCTGCTTCTTCTCGCCTCTTCGGGCGTTGAGGCTGGGGCTGGGACAGGGTGGACTGTCTACCCACCATTGGCAGGAAACCTGGCCCACGCAGGCGCATCTGTCGACCTAACAATCTTTTCTCTCCACCTCGCCGGGATCTCTTCCATTCTTGGCGCCATTAACTTTATCACCACAATCCTAAACATGAAGCCACCCGCTATCTCGCAGTATCAAACGCCCCTCTTCGTATGGGCCGTTCTTATCACAGCCGTTCTCCTCCTTCTCTCACTGCCCGTCCTCGCCGCCGGCATCACCATGCTACTTACAGACC

>HAP39_JAFL

ATCCGCGCGGAG-CTCAGCCAGCCCGGCGCACTACTTGGGGACGACCAGATTTATAACGTAATTGTCACTGCCCACGCCTTTGTAATGATTTTCTTTATAGTAATACCAATTATGATTGGGGGCTTTGGGAACTGACTAATTCCCCTAATGATTGGGGCCCCCGACATGGCCTTCCCCCGAATGAACAACATGAGCTTCTGGCTTTTGCCCCCCTCATTCCTGCTCCTTCTCGCCTCTTCGGGCGTTGAGGCTGGGGCTGGGACAGGGTGGACTGTCTACCCACCATTGGCAGGAAACCTGGCCCACGCAGGCGCATCTGTCGACCTAACAATCTTTTCTCTCCACCTCGCCGGGATTTCTTCCATTCTTGGCGCCATTAACTTTATCACCACAATCCTAAACATGAAGCCACCCGCTATCTCGCAGTATCAAACGCCCCTCTTCGTATGGGCCGTTCTTATCACAGCCGTTCTCCTCCTTCTCTCACTGCCCGTCCTCGCCGCCGGCATCACCATGCTACTTACAGACC

>HAP40_JAFL

ATCCGCGCGGAG-CTCAGCCAGCCCGGCGCACTACTTGGGGACGACCAGATTTATAACGTAATTGTCACTGCCCACGCCTTTGTAATGATTTTCTTTATAGTAATACCAATTATGATTGGGGGCTTTGGGAACTGACTAATTCCCCTAATGATTGGGGCCCCCGACATGGCCTTCCCCCGAATGAACAACATGAGCTTCTGACTTTTGCCCCCCTCATTCCTGCTCCTTCTCGCCTCTTCGGGCGTTGAGGCTGGGGCTGGGACAGGGTGGACTGTCTACCCACCATTGGCAGGAAACCTGGCCCACGCAGGCGCATCTGTCGACCTAACAATCTTTTCTCTCCACCTCGCCGGGATCTCTTCCATTCTTGGCGCCATTAACTTTATCACCACAATCCTAAACATGAAGCCACCCGCTATCTCGCAGTATCAAACGCCCCTCTTCGTATGGGCCGTTCTTATCACAGCCGTTCTCCTCCTTCTCTCACTGCCCGTCCTCGCCGCCGGCATCACCATGCTACTTACAGACC

>HAP41_JAFL

ATCCGCGCGGAG-CTCAGCCAGCCCGGCGCACTACTTGGGGACGACCAGATTTATAACGTAATTGTCACTGCCCACGCCTTTGTAATGATTTTCTTTATAGTAATACCAATTATGATTGGGGGCTTTGGGAACTGACTAATTCCCCTAATGATTGGGGCCCCCGACATGGCCTTCCCCCGAATGAACAACATGAGCTTCTGGCTTTTGCCCCCCTCATTCCTGCTCCTTCTCGCCTCTTCGGGCGTTGAGGCTGGGGCTGGGACAGGGTGGACTGTCTACCCACCATTGGCAGGAAACCTGGCCCACGCAGGCGCATCTGTCGACCTAACAATCTTTTCTCTCCACCTCGCCGGGATCTCTTCCATTCTTGGCGCCATTAACTTTATCACCACAATCCTAAACATGAAGCCACCCGCTATCTCGCAGTATCAAACGCCCCTCTTCGTATGGGCCGTTCTTATCACAGCCGTTCTCCTCCTTCTCTCACTGCCCGTCCTCGCCGCCGGCATCACCATGCTACTTACAGACC

>HAP42_IRFL

ATCCGCGCGGAA-CTCAGCCAGCCCGGCGCACTACTTGGGGACGACCAGATTTATAACGTAATTGTCACTGCCCACGCCTTTGTAATGATTTTCTTTATAGTAATACCAATTATGATTGGGGGCTTTGGGAACTGACTAATTCCCCTAATGATTGGGGCCCCCGACATGGCCTTCCCCCGAATGAACAACATGAGCTTCTGGCTTCTGCCCCCCTCATTCCTGCTCCTTCTCGCCTCTTCGGGCGTTGAGGCTGGGGCTGGGACAGGATGGACTGTCTACCCACCATTGGCAGGAAACCTGGCCCACGCAGGCGCATCTGTCGACCTAACAATCTTTTCTCTCCACCTCGCCGGGATTTCTTCCATTCTTGGCGCCATTAACTTTATCACCACAATCCTAAACATGAAGCCACCCGCTATCTCGCAGTATCAAACGCCCCTCTTCGTATGGGCCGTTCTTATCACAGCCGTTCTCCTCCTTCTCTCACTGCCCGTCCTCGCCGCCGGCATCACCATGCTACTTACAGACC

>HAP43_IRFL

ATCCGCGCGGAA-CTCAGCCAGCCCGGCGCACTACTTGGGGACGACCAGATTTATAACGTAATTGTCACTGCCCACGCCTTTGTAATGATTTTCTTTATAGTAATACCAATTATGATTGGGGGCTTTGGGAACTGACTAATTCCCCTAATGATTGGGGCCCCCGACATGGCCTTCCCCCGAATGAACAACATGAGCTTCTGGCTTTTGCCCCCCTCATTCCTGCTCCTTCTCGCCTCTTCGGGCGTTGAGGCTGGGGCTGGGACAGGATGGACTGTCTACCCACCATTGGCAGGAAACCTGGCCCACGCAGGCGCATCTGTCGACCTAACAATCTTTTCTCTCCACCTCGCCGGGATTTCTTCCATTCTTGGCGCCATTAACTTTATCACCACAATCCTAAACATGAAGCCACCCGCTATCTCGCAGTATCAAACGCCCCTCTTCGTATGGGCCGTTCTTATCACAGCCGTTCTCCTCCTTCTCTCACTGCCCGTCCTCGCCGCCGGCATCACCATGCTACTTACAGACC

>HAP44_IRFL

ATCCGCGCGGAA-CTCAGCCAGCCCGGCGCACTACTTGGGGACGACCAGATTTATAACGTAATTGTCACTGCCCACGCCTTTGTAATGATTTTCTTTATAGTAATACCAATTATGATTGGGGGCTTTGGGAACTGACTAATTCCCCTAATGATTGGGGCCCCCGACATGGCCTTCCCCCGAATGAACAACATGAGCTTCTGGCTTTTGCCCCCCTCATTCCTGCTCCTTCTCGCCTCTTCGGGCGTTGAGGCTGGGGCTGGGACAGGGTGGACTGTCTACCCACCATTGGCAGGAAACCTGGCCCACGCAGGCGCATCTGTCGACCTAACAATCTTTTCTCTCCACCTCGCCGGGATTTCTTCCATTCTTGGCGCCATTAACTTTATCACCACAATCCTAAACATGAAGCCACCCGCTATCTCGCAGTATCAAACGCCCCTCTTCGTATGGGCCGTTCTTATCACAGCCGTTCTCCTCCTTCTCTCACTGCCCGTCCTCGCCGCCGGCATCACTATGCTACTTACAGACC

>HAP45_IRFL

ATCCGCGCGGAA-CTCAGCCAGCCCGGCGCACTACTTGGGGACGACCAGATTTATAACGTAATTGTCACTGCCCACGCCTTTGTAATGATTTTCTTTATAGTAATACCAATTATGATTGGGGGCTTTGGGAACTGACTAATTCCCCTAATGATTGGGGCCCCCGACATGGCCTTCCCCCGAATGAACAACATGAGCTTCTGGCTTTTGCCCCCCTCATTCCTGCTCCTTCTCGCCTCTTCGGGCGTTGAGGCTGGGGCTGGGACAGGGTGGACTGTCTACCCACCATTGGCAGGAAACCTGGCCCACGCAGGCGCATCTGTCGACCTAACAATCTTTTCTCTCCACCTCGCCGGGATTTCTTCCATTCTTGGCGCCATTAACTTTATCACCACAATCCTAAACATGAAGCCACCCGCCATCTCGCAGTATCAAACGCCCCTCTTCGTATGGGCCGTTCTTATCACAGCCGTTCTCCTCCTTCTCTCACTGCCCGTCCTCGCCGCCGGCATCACCATGCTACTTACAGACC

>HAP46_IRFL

ATCCGCGCGGAA-CTCAGCCAGCCCGGCGCACTACTTGGGGACGACCAGATTTATAACGTAATTGTCACTGCCCACGCCTTTGTAATGATTTTCTTTATAGTAATACCAATTATGATTGGGGGCTTTGGGAACTGACTAATTCCCCTAATGATTGGGGCCCCCGACATGGCCTTCCCCCGAATGAACAACATGAGCTTCTGGCTTTTGCCCCCCTCATTCCTGCTCCTTCTCGCCTCTTCGGGCGTTGAGGCTGGGGCTGGGACAGGGTGGACTGTCTACCCACCATTGGCAGGAAACCTGGCCCACGCAGGCGCATCTGTCGACCTAACAATCTTTTCTCTCCACCTCGCCGGGATTTCTTCCATTCTTGGCGCCATTAACTTTATCACCACAATCCTAAACATGAAGCCACCCGCTATCTCGCAGTATCAAACGCCCCTCTTCGTTTGGGCCGTTCTTATCACAGCCGTTCTCCTCCTTCTCTCACTGCCCGTCCTCGCCGCCGGCATCACCATGCTACTTACAGACC

>HAP47_IRFL

ATCCGCGCGGAA-CTCAGCCAGCCCGGCGCACTACTTGGGGACGACCAGATTTATAACGTAATTGTCACTGCCCACGCCTTTGTAATGATTTTCTTTATAGTAATACCAATTATGATTGGGGGCTTTGGGAACTGACTAATTCCCCTAATGATTGGGGCCCCCGACATGGCCTTCCCCCGAATGAACAACATGAGCTTCTGGCTTTTGCCCCCCTCATTCCTGCTCCTTCTCGCCTCTTCGGGCGTTGAGGCTGGGGCTGGGACAGGGTGGACTGTCTACCCGCCATTGGCAGGAAACCTGGCCCACGCAGGCGCATCTGTCGACCTAACAATCTTTTCTCTCCACCTCGCCGGGATTTCTTCCATTCTTGGCGCCATTAACTTTATCACCACAATCCTAAACATGAAGCCACCCGCTATCTCGCAGTATCAAACGCCCCTCTTCGTATGGGCCGTTCTTATCACAGCCGTTCTCCTCCTTCTCTCACTGCCCGTCCTCGCCGCCGGCATCACCATGCTACTTACAGACC

>HAP48_IRFL

ATCCGCGCGGAA-CTCAGCCAGCCCGGCGCACTACTTGGGGACGACCAGATTTATAACGTAATTGTCACTGCCCACGCCTTTGTAATGATTTTCTTTATAGTAATACCAATTATGATTGGGGGCTTTGGGAACTGACTAATTCCCCTAATGATTGGGGCCCCCGACATGGCCTTCCCCCGAATGAACAACATGAGCTTCTGGCTTTTGCCCCCCTCATTCCTGCTCCTTCTCGCCTCTTCGGGCGTTGAGGCTGGGGCTGGGACAGGGTGGACTGTCTACCCACCATTGGCAGGAAACCTGGCCCACGCAGGCGCATCTGTCGACCTAACAATCTTTTCTCTCCACCTCGCCGGGATTTCTTCCATTCTTGGCGCCATTAACTTTATCACCACAATCCTAAACATGAAGCCACCCGCTATCTCGCAGTATCAAACGCCCCTCTTCGTATGGGCCGTTCTTATCACAGCCGTTCTCCTCCTTCTCTCACTGCCCGTCCTCGCCGCCGGCATCACCATGCTACTTACAGACC

>HAP49_HRNY

ATCCGCGCGGAA-CTCAGCCAGCCCGGCGCACTACTTGGGGACGACCAGATTTATAACGTAATTGTCACTGCCCACGCCTTTGTAATGATTTTCTTTATAGTAATACCAATTATGATTGGGGGCTTTGGGAACTGACTAATTCCCCTAATGATTGGGGCCCCCGACATGGCCTTCCCCCGAATGAACAACATGAGCTTCTGGCTTTTGCCCCCCTCATTCCTGCTCCTTCTCGCCTCTTCGGGCGTTGAGGCTGGGGCTGGGACAGGGTGGACTGTCTACCCACCGTTGGCAGGAAACCTGGCCCACGCAGGCGCATCTGTCGACCTAACAATCTTTTCTCTCCACCTCGCCGGGATCTCTTCCATTCTTGGCGCCATTAACTTTATCACCACAATCCTAAACATGAAGCCACCCGCTATCTCGCAGTATCAAACGCCCCTCTTCGTATGGGCCGTTCTTATCACAGCCGTTCTCCTCCTTCTCTCACTGCCCGTCCTCGCCGCCGGCATCACCATGCTACTTACAGACC

>HAP50_VIVA

ATCCGCGCGGAA-CTCAGCCAGCCCGGCGCACTACTTGGGGACGACCAGATTTATAACGTAATTGTCACTGCCCACGCCTTTGTAATGATTTTCTTTATAGTAATACCAATTATGATTGGGGGCTTTGGGAACTGACTAATTCCCCTAATGATTGGGGCCCCCGACATGGCCTTCCCCCGAATGAACAACATGAGCTTCTGGCTTTTGCCCCCCTCATTCCTGCTCCTTCTCGCCTCTTCGGGCGTTGAGGCTGGGGCTGGGACAGGGTGGACTGTCTACCCACCATTGGCAGGAAACCTGGCCCACGCAGGCGCATCTGTCGACCTAACAATCTTTTCTCTCCACCTCGCCGGGATCTCTTCCATTCTTGGTGCCATTAACTTTATCACCACAATCCTAAACATGAAGCCACCCGCTATCTCGCAGTATCAAACGCCCCTCTTCGTATGGGCCGTTCTTATCACAGCCGTTCTCCTCCTTCTCTCACTGCCCGTCCTCGCCGCCGGCATCACCATGCTACTTACAGACC

>HAP51_PRNY

ATCCGCGCGGAA-CTCAGCCAGCCCGGCGCACTACTTGGGGACGACCAGATTTATAACGTAATTGTCACTGCCCACGCCTTTGTAATGATTTTCTTTATAGTAATACCAATTATGATTGGGGGCTTTGGGAACTGACTAATTCCCCTAATGATTGGGGCCCCCGACATGGCCTTCCCCCGAATGAACAACATGAGCTTCTGGCTTTTGCCCCCCTCATTCCTGCTCCTTCTCGCCTCTTCGGGCGTTGAGGCTGGGGCTGGGACAGGGTGGACTGTCTACCCACCATTGGCAGGAAACCTGGCCCACGCAGGCGCATCTGTCGACCTAACAATCTTTTCTCTCCACCTCGCCGGGATCTCTTCCATTCTTGGCGCCATTAACTTTATCACCACAATCCTAAACATGAAGCCACCCGCTATCTCGCAGTATCAAACGCCCCTCTTCGTATGGGCCGTTCTTATCACAGCCGTTCTCCTCCTTCTCTCACTGCCCGTCCTCGCCGCCGGCATCACCATGCTACTTACAGACC

>HAP52_CKFL

ATCCGCGCGGAA-CTCAGCCAGCCCGGCGCACTACTTGGGGATGACCAGATTTATAACGTAATTGTTACTGCTCATGCCTTCGTAATAATTTTCTTTATAGTAATACCAATTATGATTGGAGGTTTTGGGAACTGACTGATTCCCCTAATGATTGGGGCCCCCGACATGGCCTTCCCCCGAATAAATAACATAAGCTTCTGGCTTTTACCCCCCTCATTCCTGCTCCTTCTCGCCTCTTCAGGCGTTGAAGCTGGGGCCGGAACAGGGTGAACTGTCTACCCCCCACTAGCAGGCAACCTAGCCCACGCAGGCGCATCTGTCGACCTAACAATTTTTTCTCTCCACCTCGCTGGGATCTCTTCCATTCTTGGGGCCATTAACTTTATCACCACAATCCTAAACATGAAGCCGCCCGCTGTCTCGCAGTATCAAACGCCCCTCTTCGTATGGGCCGTCCTTATCACAGCCGTCCTTCTCCTTCTTTCACTGCCCGTCCTCGCCGCCGGCATCACCATGCTACTCACAGACC

>HAP53_TBFL

ATCCGCGCGGAA-CTCAGCCAGCCCGGCGCACTACTTGGGGATGACCAGATTTATAACGTAATTGTTACTGCTCACGCCTTCGTAATAATTTTCTTTATAGTAATACCAATTATGATTGGAGGTTTTGGGAACTGACTGATTCCCCTAATGATTGGGGCCCCCGACATGGCCTTCCCCCGAATAAATAACATAAGCTTCTGGCTTTTACCCCCCTCATTCCTGCTCCTTCTCGCCTCTTCAGGCGTTGAAGCTGGGGCCGGAACAGGGTGAACTGTCTACCCCCCACTAGCAGGCAACCTAGCCCACGCAGGCGCATCTGTCGACCTAACAATTTTTTCTCTCCACCTCGCTGGGGTCTCTTCCATTCTTGGGGCCATTAACTTTATCACCACAATCCTAAACATGAAGCCGCCCGCTGTCTCGCAGTATCAAACGCCCCTCTTCGTATGGGCCGTCCTTATCACAGCCGTCCTTCTCCTTCTTTCACTGCCCGTCCTCGCCGCCGGCATCACCATGCTACTCACAGACC

>HAP54_CKFL

ATCCGCGCGGAA-CTCAGCCAGCCCGGCGCACTACTTGGGGATGACCAGATTTATAACGTAATTGTTACTGCTCACGCCTTCGTAATAATTTTCTTTATAGTAATACCAATTATGATTGGAGGTTTTGGGAACTGACTGATTCCCCTAATGATTGGGGCCCCCGACATGGCCTTCCCCCGAATAAATAACATAAGCTTCTGGCTTTTACCCCCCTCATTCCTGCTCCTTCTCGCCTCTTCAGGCGTTGAAGCTGGGGCCGGAACAGGGTGAACTGTCTACCCCCCACTAGCAGGCAACCTAGCCCACGCAGGCGCATCTGTCGACCTAACAATTTTTTCTCTCCACCTCGCTGGGATCTCTTCCATTCTTGGGGCCATTAACTTTATCACCACAATCCTAAACATGAAGCCGCCCGCTGTCTCGCAGTATCAAACGCCCCTCTTCGTATGGGCCGTCCTTATCACAGCTGTCCTTCTCCTTCTTTCACTGCCCGTCCTCGCCGCCGGCATCACCATGCTACTCACAGACC

>HAP55_CKFL

ATCCGCGCGGAA-CTCAGCCAGCCCGGCGCACTACTTGGGGATGACCAGATTTATAACGTAATTGTTACTGCTCACGCCTTCGTAATAATTTTCTTTATAGTAATACCAATTATGATTGGAGGTTTTGGGAACTGACTGATTCCCCTAATGATTGGGGCCCCCGACATGGCCTTCCCCCGAATAAATAACATAAGCTTCTGGCTTTTACCCCCCTCATTCCTGCTCCTTCTCGCCTCTTCAGGCGTTGAAGCTGGGGCCGGAACAGGGTGAACTGTCTACCCCCCACTAGCAGGCAACCTAGCCCACGCAGGCGCATCTGTCGACCTAACAATTTTTTCTCTCCACCTCGCTGGGATCTCTTCCATTCTTGGGGCCATTAACTTTATCACCACAATCCTAAACATGAAGCCGCCCGCTGTCTCGCAGTATCAAACGCCCCTCTTCGTATGGGCCGTCCTTATCACAGCCGTCCTTCTCCTCCTTTCACTGCCCGTCCTCGCCGCCGGCATCACCATGCTACTCACAGACC

>HAP56_CKFL

ATCCGCGCGGAA-CTCAGCCAGCCCGGCGCACTACTTGGGGATGACCAGATTTATAACGTAATTGTTACTGCTCACGCCTTCGTAATAATTTTCTTTATAGTAATACCAATTATGATTGGAGGTTTTGGGAACTGACTGATTCCCCTAATGATTGGGGCCCCAGACATGGCCTTCCCCCGAATAAATAACATAAGCTTCTGGCTTTTACCCCCCTCATTCCTGCTCCTTCTCGCCTCTTCAGGCGTTGAAGCTGGGGCCGGAACAGGGTGAACTGTCTACCCCCCACTAGCAGGCAACCTAGCCCACGCAGGCGCATCTGTCGACCTAACAATTTTTTCTCTCCACCTCGCTGGGATCTCTTCCATTCTTGGGGCCATTAACTTTATCACCACAATCCTAAACATGAAGCCGCCCGCTGTCTCGCAGTATCAAACGCCCCTCTTCGTATGGGCCGTCCTTATCACAGCCGTCCTTCTCCTTCTTTCACTGCCCGTCCTCGCCGCCGGCATCACCATGCTACTCACAGACC

>HAP57_CKFL

ATCCGCGCGGAA-CTCAGCCAGCCCGGCGCACTACTTGGGGATGACCAGATTTATAACGTAATTGTTACTGCTCACGCCTTCGTAATAATTTTCTTTATAGTAATACCAATTATGATTGGAGGTTTTGGGAACTGACTGATTCCCCTAATGATTGGGGCCCCCGACATGGCCTTCCCCCGAATAAATAACATAAGCTTCTGGCTTTTACCCCCCTCATTCCTGCTCCTTCTCGCCTCTTCAGGCGTTGAAGCTGGGGCCGGAACAGGGTGAACTGTCTACCCCCCACTAGCAGGCAACCTAGCCCACGCAGGCGCATCTGTCGACCTAACAATTTTTTCTCTCCACCTCGCTGGGATCTCTTCCATTCTTGGGGCCATTAACTTTATCACCACAATCCTAAACATGAAGCCGCCCGCTGTCTCGCAGTATCAAACGCCCCTCTTCGTATGGGCCGTCCTTATCACAGCCGTCCTTCTCCTTCTTTCACTGCCCGTCCTCGCCGCCGGCATCACCATGCTACTCACAGACC

>HAP58_TBFL

ATCCGCGCGGAA-CTCAGCCAGCCCGGCGCACTACTTGGGGATGACCAGATTTATAACGTAATTGTTACTGCTCACGCCTTCGTAATAATTTTCTTTATAGTAATACCAATTATGATTGGAGGTTTTGGGAACTGACTGATTCCCCTAATGATTGGGGCCCCCGACATGGCCTTCCCCCGAATAAATAACATAAGCTTCTGGCTTTTACCCCCCTCATTCCTGCTCCTTCTCGCCTCTTCAGGCGTTGAAGCTGGGGCCGGAACAGGGTGAACTGTCTACCCCCCACTAGCAGGCAACCTCGCCCACGCAGGCGCATCTGTCGACCTAACAATTTTTTCTCTCCACCTCGCTGGGATCTCTTCCATTCTTGGGGCCATTAACTTTATCACCACAATCCTAAACATGAAGCCGCCCGCTGTCTCGCAGTATCAAACGCCCCTCTTCGTATGGGCCGTCCTTATCACAGCCGTCCTTCTCCTTCTTTCACTGCCCGTCCTCGCCGCCGGCATCACCATGCTACTCACAGACC

>HAP59_CKFL

ATCCGCGCGGAA-CTCAGCCAGCCCGGCGCACTACTTGGGGATGACCAGATTTATAACGTAATTGTTACTGCTCACGCCTTCGTAATAATTTTCTTTATAGTAATACCAATTATGATTGGAGGTTTTGGGAACTGACTGATTCCCCTAATGATTGGGGCCCCCGACATGGCCTTCCCCCGAATAAATAACATAAGCTTCTGGCTTTTACCCCCCTCATTCCTGCTCCTTCTCGCCTCTTCAGGCGTTGAAGCTGGGGCCGGAACAGGGTGAACTGTCTACCCCCCACTAGCAGGCAACCTGGCCCACGCAGGCGCATCTGTCGACCTAACAATTTTTTCTCTCCACCTCGCTGGGATCTCTTCCATTCTTGGGGCCATTAACTTTATCACCACAATCCTAAACATGAAGCCGCCCGCTGTCTCGCAGTATCAAACGCCCCTCTTCGTATGGGCCGTCCTTATCACAGCCGTCCTTCTCCTTCTTTCACTGCCCGTCCTCGCCGCCGGCATCACCATGCTACTCACAGACC

>HAP60_CKFL

ATCCGCGCGGAA-CTCAGCCAGCCCGGCGCACTACTTGGGGATGACCAGATTTATAACGTAATTGTTACTGCTCACGCCTTCGTAATAATTTTCTTTATAGTAATACCAATTATGATTGGAGGTTTTGGGAACTGACTGATTCCCCTAATGATTGGGGCCCCCGACATGGCCTTCCCCCGAATAAATAACATAAGCTTCTGGCTTTTACCCCCCTCATTCCTGCTCCTTCTCGCCTCTTCAGGCGTTGAAGCTGGGGCCGGAACAGGGTGAACTGTCTACCCCCCACTAGCAGGCAACCTAGCCCACGCAGGCGCATCTGTCGACCTAACAATTTTTTCTCTCCACCTCGCTGGGATCTCTTCCATTCTTGGGGCCATTAACTTTATCACCACAATCCTAAACATGAAGCCACCCGCTGTCTCGCAGTATCAAACGCCCCTCTTCGTATGGGCCGTCCTTATCACAGCCGTCCTTCTCCTTCTTTCACTGCCCGTCCTCGCCGCCGGCATCACCATGCTACTCACAGACC

>HAP61_CKFL

ATCCGCGCGGAA-CTCAGCCAGCCCGGCGCACTACTTGGGGATGACCAGATTTATAACGTAATTGTTACTGCTCACGCCTTCGTAATAATTTTCTTTATAGTAATACCAATTATGATTGGAGGTTTTGGGAACTGACTGATTCCCCTAATGATTGGGGCCCCCGACATGGCCTTCCCCCGAATAAATAACATAAGCTTCTGGCTTTTACCCCCCTCATTCCTGCTCCTTCTCGCCTCTTCAGGCGTTGAAGCTGGGGCCGGAACAGGGTGAACTGTCTACCCCCCACTAGCAGGCAACCTAGCCCACGCAGGCGCATCTGTCGACCTAACAATTTTTTCTCTCCACCTCGCTGGGATCTCTTCCATTCTTGGGGCCATTAACTTTATCACCACAATCCTAAACATGAAGCCGCCCGCTGTCTCGCAGTATCAAACGCCCCTCTTCGTATGGGCCGTCCTTATCACAGCCGTCCTCCTCCTTCTTTCACTGCCCGTCCTCGCCGCCGGCATCACCATGCTACTCACAGACC

>HAP62_TBFL

ATCCGCGCGGAA-CTCAGCCAGCCCGGCGCACTACTTGGGGATGACCAGATTTATAACGTAATTGTTACTGCTCACGCCTTCGTAATAATTTTCTTTATAGTAATACCAATTATGATTGGAGGTTTTGGGAACTGACTGATTCCCCTAATGATTGGGGCCCCCGACATGGCCTTCCCCCGAATAAATAACATAAGCTTCTGGCTTTTACCCCCCTCATTCCTGCTCCTTCTCGCCTCTTCAGGCGTTGAAGCTGGGGCCGGAACAGGGTGAACTGTCTACCCCCCACTAGCAGGCAACCTAGCCCACGCAGGCGCATCTGTCGACCTAACAATTTTTTCTCTCCACCTCGCCGGGATCTCTTCCATTCTTGGAGCCATTAACTTTATCACCACAATCCTAAACATGAAGCCGCCCGCTGTCTCGCAGTATCAAACGCCCCTCTTCGTATGGGCCGTCCTTATCACAGCCGTCCTTCTCCTTCTTTCACTGCCCGTCCTCGCCGCCGGCATCACCATGCTACTCACAGACC

>HAP63_DEFL

ATCCGCGCGGAA-CTCAGCCAGCCCGGCGCACTACTTGGAGATGACCAGATTTATAACGTAATTGTTACTGCTCACGCCTTCGTAATGATTTTCTTTATAGTAATACCAATTATGATTGGAGGGTTTGGGAACTGACTGGTTCCTCTAATGATTGGGGCCCCCGACATGGCCTTCCCACGAATGAATAACATGAGCTTCTGGCTTTTACCCCCCTCATTCCTGCTCCTCCTCGCCTCTTCAGGCGTTGAAGCTGGGGCTGGGACAGGGTGGACCGTCTACCCCCCACTGGCAGGCAATCTAGCCCACGCAGGCGCATCTGTCGACCTAACAATCTTTTCTCTCCACCTCGCCGGGATCTCTTCCATTCTCGGGGCCATTAACTTTATCACCACAATCCTAAACATAAAACCGCCCGCTGTCTCGCAGTATCAAACGCCCCTCTTCGTGTGGGCCGTCCTTATCACAGCCGTCCTTCTCCTTCTTTCACTGCCCGTCCTCGCCGCCGGCATCACCATGCTACTTACAGACC

>HAP64_DEFL

ATCCGCGCGGAA-CTCAGCCAGCCCGGCGCACTACTTGGAGATGACCAGATTTATAACGTAATTGTTACTGCTCACGCCTTCGTAATGATTTTCTTTATAGTAATACCAATTATGATTGGAGGGTTTGGGAACTGACTGGTTCCTCTAATGATTGGGGCCCCCGACATGGCCTTCCCACGAATGAATAACATGAGCTTCTGGCTTTTACCCCCCTCATTCCTGCTCCTCCTCGCCTCTTCAGGCGTTGAAGCTGGGGCTGGGACAGGGTGGACCGTCTACCCCCCACTGGCAGGCAATCTAGCCCACGCAGGCGCATCTGTCGACCTAACAATCTTTTCTCTCCACCTCGCCGGGATCTCTTCCATTCTCGGGGCCATTAACTTTATCACCACAATCCTAAACATGAAACCGCCCGCTGTCTCGCAGTATCAAACGCCCCTCTTCGTGTGGGCCGTCCTTATCACAGCCGTCCTTCTCCTTCTTTCACTGCCCGTCCTCGCCGCCGGCATCACCATGTTACTTACAGACC

>HAP65_DEFL

ATCCGCGCGGAA-CTCAGCCAGCCCGGCGCACTACTTGGAGATGACCAGATTTATAATGTAATTGTTACTGCTCACGCCTTCGTAATGATTTTCTTTATAGTAATACCAATTATGATTGGAGGGTTTGGGAACTGACTGGTTCCTCTAATGATTGGGGCCCCCGACATGGCCTTCCCACGAATGAATAACATGAGCTTCTGGCTTTTACCCCCCTCATTCCTGCTCCTCCTCGCCTCTTCAGGCGTTGAAGCTGGGGCTGGGACAGGGTGGACCGTCTACCCCCCACTGGCAGGCAATCTAGCCCACGCAGGCGCATCTGTCGACCTAACAATCTTTTCTCTCCACCTCGCCGGGATCTCTTCCATTCTCGGGGCCATTAACTTTATCACCACAATCCTAAACATGAAACCGCCCGCTGTCTCGCAGTATCAAACGCCCCTCTTCGTGTGGGCCGTCCTTATCACAGCCGTCCTTCTCCTTCTTTCACTGCCCGTCCTCGCCGCCGGCATCACCATGCTACTTACAGACC

>HAP66_DEFL

ATCCGCGCGGAA-CTCAGCCAGCCCGGCGCACTACTTGGAGATGACCAGATTTATAACGTAATTGTTACTGCTCACGCCTTCGTAATGATTTTCTTTATAGTAATACCAATTATGATTGGAGGGTTTGGGAACTGACTGGTTCCTCTAATGATTGGGGCCCCCGACATGGCCTTCCCACGAATGAATAACATGAGCTTCTGGCTTTTACCCCCCTCATTCCTGCTCCTCCTCGCCTCTTCAGGCGTTGAAGCTGGGGCTGGGACAGGGTGGACCGTCTACCCCCCACTGGCAGGCAATCTAGCCCACGCAGGCGCATCTGTCGACCTAACAATCTTTTCTCTCCACCTCGCCGGGATCTCTTCCATTCTCGGGGCCATTAACTTTATCACCACAATCCTAAACATGAAACCGCCCGCTGTCTCGCAGTATCAAACGCCCCTCTTCGTGTGGGCCGTCCTTATCACAGCCGTCCTTCTCCTTCTTTCACTGCCCGTCCTCGCCGCCGGCATCACCATGCTACTTACAGACC

>HAP67_DEFL

ATCCGCGCGGAA-CTCAGCCAGCCCGGCGCACTACTTGGAGATGACCAGATTTATAACGTAATTGTTACTGCTCACGCCTTCGTAATGATTTTCTTTATAGTAATACCAATTATGATTGGAGGGTTTGGGAACTGACTGGTTCCTCTAATGATTGGGGCCCCCGACATGGCCTTCCCACGAATGAATAACATGAGCTTCTGGCTTTTACCCCCCTCATTCCTGCTCCTCCTCGCCTCTTCAGGCGTTGAAGCTGGGGCTGGGACAGGGTGGACCGTCTACCCCCCACTGGCAGGCAATCTAGCCCACGCAGGCGCATCTGTCGACCTAACAATCTTTTCTCTCCACCTCGCCGGGATCTCTTCCATTCTCGGAGCCATTAACTTTATCACCACAATCCTAAACATGAAACCGCCCGCTGTCTCGCAGTATCAAACGCCCCTCTTCGTGTGGGCCGTCCTTATCACAGCCGTCCTTCTCCTTCTTTCACTGCCCGTCCTCGCCGCCGGCATCACCATGCTACTTACAGACC

>HAP68_APFL

ATCCGCGCGGAA-CTCAGCCAGCCCGGCGCACTACTTGGAGATGACCAGATTTATAACGTAATTGTTACTGCTCACGCCTTCGTAATGATTTTCTTTATAGTAATACCAATTATGATTGGAGGGTTTGGGAACTGACTGATTCCTCTAATGATTGGGGCCCCCGACATGGCCTTCCCACGAATGAATAACATGAGCTTCTGGCTTTTACCCCCCTCATTCCTGCTCCTCCTCGCCTCTTCAGGCGTTGAAGCTGGGGCTGGGACAGGGTGAACCGTCTACCCCCCACTGGCAGGCAATCTAGCCCACGCAGGCGCATCTGTCGACCTAACAATCTTTTCTCTCCACCTCGCCGGGATCTCTTCCATTCTCGGGGCCATTAACTTTATCACCACAATCCTAAACATGAAACCGCCCGCTGTCTCGCAGTATCAAACGCCCCTCTTCGTGTGGGCCGTCCTTATCACAGCCGTCCTTCTCCTTCTTTCACTGCCCGTCCTCGCCGCCGGCATCACCATGCTACTTACAGACC

>HAP69_APFL

ATCCGCGCGGAA-CTCAGCCAGCCCGGCGCACTACTTGGAGATGACCAGATTTATAACGTAATTGTTACTGCTCACGCCTTCGTAATGATTTTCTTTATAGTAATACCAATTATGATTGGAGGGTTTGGGAACTGACTGATTCCTCTAATGATTGGGGCCCCCGACATGGCCTTCCCACGAATGAATAACATGAGCTTCTGGCTTTTACCCCCCTCATTCCTGCTCCTCCTCGCCTCTTCAGGCGTTGAAGCTGGGGCTGGGACAGGGTGGACCGTCTACCCCCCACTGGCAGGCAATCTAGCCCACGCAGGCGCATCTGTCGACCTAACAATCTTTTCTCTCCACCTCGCCGGGATCTCTTCCATTCTCGGGGCCATTAACTTTATCACCACAATCCTAAACATGAAACCGCCCGCTGTCTCGCAGTATCAAACGCCCCTCTTCGTGTGGGCCGTCCTTATCACAGCCGTCCTTCTCCTTCTTTCACTGCCCGTCCTCGCCGCCGGCATCACCATGCTACTTACAGACC

>HAP70_GATX

ATCCGCGCGGAA-CTCAGCCAGCCCGGCGCACTACTTGGAGATGACCAGATTTATAACGTAATTGTTACTGCTCACGCCTTCGTAATGATTTTCTTTATAGTAATACCAATTATGATTGGAGGGTTTGGGAACTGACTGATTCCTCTAATGATTGGGGCCCCCGACATGGCCTTCCCACGAATGAATAACATGAGCTTCTGGCTTTTACCCCCCTCATTCCTGCTCCTCCTCGCCTCTTCAGGCGTTGAAGCTGGGGCTGGGACAGGGTGGACCGTCTACCCCCCACTGGCAGGCAATCTAGCCCACGCAGGCGCATCTGTCGACCTGACAATCTTTTCTCTCCACCTCGCCGGGATCTCTTCCATTCTCGGGGCCATTAACTTTATCACCACTATCCTAAACATGAAACCGCCCGCTGTCTCGCAGTATCAAACGCCCCTCTTCGTGTGGGCCGTCCTTATCACAGCCGTCCTTCTCCTTCTTTCACTGCCCGTCCTCGCCGCCGGCATCACCATGCTACTTACAGACC

>HAP71_GATX

ATCCGCGCGGAA-CTCAGCCAGCCCGGCGCACTACTTGGAGATGACCAGATTTATAACGTAATTGTTACTGCTCACGCCTTCGTAATGATTTTCTTTATAGTAATACCAATTATGATTGGAGGGTTTGGGAACTGACTGATTCCTCTAATGATTGGGGCCCCCGACATGGCCTTCCCACGAATGAATAACATGAGCTTCTGGCTTTTGCCCCCCTCATTCCTGCTCCTCCTCGCCTCTTCAGGCGTTGAAGCTGGGGCTGGGACAGGGTGGACCGTCTACCCCCCACTGGCAGGCAATCTAGCCCACGCAGGCGCATCTGTCGACCTGACAATCTTTTCTCTCCACCTCGCCGGGATCTCTTCCATTCTCGGGGCCATTAACTTTATCACCACTATCCTAAACATGAAACCGCCCGCTGTCTCGCAGTATCAAACGCCCCTCTTCGTGTGGGCCGTCCTTATCACAGCCGTCCTTCTCCTTCTTTCACTGCCCGTCCTCGCCGCCGGCATCACCATGCTACTTACAGACC

>HAP72_APFL

ATCCGCGCGGAA-CTCAGCCAGCCCGGCGCACTACTTGGAGATGACCAGATTTATAACGTAATTGTTACTGCTCACGCCTTCGTAATGATTTTCTTTATAGTAATACCAATTATGATTGGAGGGTTTGGGAACTGACTGATTCCTCTAATGATTGGGGCCCCCGACATGGCCTTCCCACGAATGAATAACATGAGCTTCTGGCTTTTGCCCCCATCATTCCTGCTCCTCCTCGCCTCTTCAGGCGTTGAAGCTGGGGCTGGGACAGGGTGGACCGTCTACCCCCCACTGGCAGGCAATCTAGCCCACGCAGGCGCATCTGTCGACCTAACAATCTTTTCTCTCCACCTCGCCGGGATCTCTTCCATTCTCGGGGCCATTAACTTTATCACCACAATCCTAAACATGAAACCGCCCGCTGTATCGCAGTATCAGACGCCCCTCTTCGTGTGGGCCGTCCTTATCACAGCCGTCCTTCTCCTTCTTTCACTGCCCGTCCTCGCCGCCGGCATCACCATGCTACTTACAGACC

>HAP73_APFL

ATCCGCGCGGAA-CTCAGCCAGCCCGGCGCACTACTTGGAGATGACCAGATTTATAACGTAATTGTTACTGCTCACGCCTTCGTAATGATTTTCTTTATAGTAATACCAATTATGATTGGAGGGTTTGGGAACTGACTGATTCCTCTAATGATTGGGGCCCCCGACATGGCCTTCCCACGAATGAATAACATGAGCTTCTGGCTTTTGCCCCCATCATTCCTGCTCCTCCTCGCCTCTTCAGGCGTTGAAGCTGGGGCTGGGACAGGGTGGACCGTCTACCCCCCACTGGCAGGCAATCTAGCCCACGCAGGCGCATCTGTCGACCTAACAATCTTTTCTCTCCACCTCGCCGGGATCTCTTCCATTCTCGGGGCCATTAACTTTATCACCACAATTCTAAACATGAAACCGCCCGCTGTATCGCAGTATCAAACGCCCCTCTTCGTGTGGGCCGTCCTTATCACAGCCGTCCTTCTCCTTCTTTCACTGCCCGTCCTCGCCGCCGGCATCACCATGCTACTTACAGACC

>HAP74_APFL

ATCCGCGCGGAA-CTCAGCCAGCCCGGCGCACTACTTGGAGATGACCAGATTTATAACGTAATTGTTACTGCTCACGCCTTCGTAATGATTTTCTTTATAGTAATACCAATTATGATTGGAGGGTTTGGGAACTGACTGATTCCTCTAATGATTGGGGCCCCCGACATGGCCTTCCCACGAATGAATAACATGAGCTTCTGGCTTTTGCCCCCATCATTCCTGCTCCTCCTCGCCTCTTCAGGCGTTGAAGCTGGGGCTGGGACAGGGTGGACCGTCTACCCCCCACTGGCAGGCAATCTAGCCCACGCAGGCGCATCTGTCGACCTAACAATCTTTTCTCTCCACCTCGCCGGGATCTCTTCCATTCTCGGGGCCATTAACTTTATTACCACAATCCTAAACATGAAACCGCCCGCTGTATCGCAGTATCAAACGCCCCTCTTCGTGTGGGCCGTCCTTATCACAGCCGTCCTTCTCCTTCTTTCACTGCCCGTCCTCGCCGCCGGCATCACCATGCTACTTACAGACC

>HAP75_APFL

ATCCGCGCGGAA-CTCAGCCAGCCCGGCGCACTACTTGGAGATGACCAGATTTATAACGTAATTGTTACTGCTCACGCCTTCGTAATGATTTTCTTTATAGTAATACCAATTATGATTGGAGGGTTTGGGAACTGACTGATTCCTCTAATGATTGGGGCCCCCGACATGGCCTTCCCACGAATGAATAACATGAGCTTCTGGCTTTTGCCCCCATCATTCCTGCTCCTCCTCGCCTCTTCAGGTGTTGAAGCTGGGGCTGGGACAGGGTGGACCGTCTACCCCCCACTGGCAGGCAATCTAGCCCACGCAGGCGCATCTGTCGACCTAACAATCTTTTCTCTCCACCTCGCCGGGATCTCTTCCATTCTCGGGGCCATTAACTTTATCACCACAATCCTAAACATGAAACCGCCCGCTGTATCGCAGTATCAAACGCCCCTCTTCGTGTGGGCCGTCCTTATCACAGCCGTCCTTCTCCTTCTTTCACTGCCCGTCCTCGCCGCCGGCATCACCATGCTACTTACAGACC

>HAP76_DEFL

ATCCGCGCGGAA-CTCAGCCAGCCCGGCGCACTACTTGGAGATGACCAGATTTATAACGTAATTGTTACTGCTCACGCCTTCGTAATGATTTTCTTTATAGTAATACCAATTATGATTGGAGGGTTTGGGAACTGACTGATTCCTCTAATGATTGGGGCCCCCGACATGGCCTTCCCACGAATGAATAACATGAGCTTCTGGCTTTTGCCCCCATCATTCCTGCTCCTCCTCGCCTCTTCAGGCGTTGAAGCTGGGGCTGGGACAGGGTGGACCGTCTACCCCCCACTGGCGGGCAATCTAGCCCACGCAGGCGCATCTGTCGACCTAACAATCTTTTCTCTCCACCTCGCCGGGATCTCTTCCATTCTCGGGGCCATTAACTTTATCACCACAATCCTAAACATGAAACCGCCCGCTGTATCGCAGTATCAAACGCCCCTCTTCGTGTGGGCCGTCCTTATCACAGCCGTCCTTCTCCTTCTTTCACTGCCCGTCCTCGCCGCCGGCATCACCATGCTACTTACAGACC

>HAP77_APFL

ATCCGCGCGGAA-CTCAGCCAGCCCGGCGCACTACTTGGAGATGACCAGATTTATAACGTAATTGTTACTGCTCACGCCTTCGTAATGATTTTCTTTATAGTAATACCAATTATGATTGGAGGGTTTGGAAACTGACTGATTCCTCTAATGATTGGGGCCCCCGACATGGCCTTCCCACGAATGAATAACATGAGCTTCTGGCTTTTGCCCCCATCATTCCTGCTCCTCCTCGCCTCTTCAGGCGTTGAAGCTGGGGCTGGGACAGGGTGGACCGTCTACCCCCCACTGGCAGGCAATCTAGCCCACGCAGGCGCATCTGTCGACCTAACAATCTTTTCTCTCCACCTCGCCGGGATCTCTTCCATTCTCGGGGCCATTAACTTTATCACCACAATCCTAAACATGAAACCGCCCGCTGTATCGCAGTATCAAACGCCCCTCTTCGTGTGGGCCGTCCTTATCACAGCCGTCCTTCTCCTTCTTTCACTGCCCGTCCTCGCCGCCGGCATCACCATGCTACTTACAGACC

>HAP78_APFL

ATCCGCGCGGAA-CTCAGCCAGCCCGGCGCACTACTTGGAGATGACCAGATTTATAACGTAATTGTTACTGCTCACGCCTTCGTAATGATTTTCTTTATAGTAATACCAATTATGATTGGAGGGTTTGGGAACTGACTGATTCCTCTAATGATTGGGGCCCCCGACATGGCCTTCCCACGAATGAATAACATGAGCTTCTGGCTTTTGCCCCCATCATTCCTGCTCCTCCTCGCCTCTTCAGGCGTTGAAGCTGGGGCTGGGACAGGGTGGACCGTCTACCCCCCACTGGCAGGCAATCTAGCCCACGCAGGCGCATCTGTCGACCTAACAATCTTTTCTCTCCACCTCGCCGGGATCTCTTCCATTCTCGGGGCCATTAACTTTATCACCACAATCCTAAACATGAAACCGCCCGCTGTATCGCAGTATCAAACGCCCCTCTTCGTGTGGGCCGTCCTTATCACAGCCGTCCTTCTCCTTCTTTCACTGCCCGTCCTCGCCGCCGGCATCACCATGCTACTTACAGACC

>HAP79_APFL

ATCCGCGCGGAA-CTCAGCCAGCCCGGCGCACTACTTGGAGACGACCAGATTTATAACGTAATTGTTACTGCTCACGCCTTCGTAATGATTTTCTTTATAGTAATACCAATTATGATTGGAGGGTTTGGGAACTGACTGATTCCTCTAATGATTGGGGCCCCCGACATGGCCTTCCCACGAATGAATAACATGAGCTTCTGGCTTTTGCCCCCATCATTCCTGCTCCTCCTCGCCTCTTCAGGCGTTGAAGCTGGGGCTGGGACAGGGTGGACCGTCTACCCCCCACTGGCAGGCAATCTAGCCCACGCAGGCGCATCTGTCGACCTAACAATCTTTTCTCTCCACCTCGCCGGGATCTCTTCCATTCTCGGGGCCATTAACTTTATCACCACAATCCTAAACATGAAACCGCCCGCTGTATCGCAGTATCAAACGCCCCTCTTCGTGTGGGCCGTCCTTATCACAGCCGTCCTTCTCCTTCTTTCACTGCCCGTCCTCGCCGCCGGCATCACCATGCTACTTACAGACC

>HAP80_EMLA

ATCCGCGCGGAA-CTCAGCCAGCCCGGCGCACTACTTGGAGATGACCAGATTTATAACGTAATTGTTACTGCTCACGCCTTCGTAATAATTTTCTTTATAGTAATACCAATTATGATTGGAGGGTTTGGGAACTGACTGATTCCTCTAATGATTGGGGCCCCCGACATGGCCTTCCCACGAATGAATAACATGAGCTTCTGGCTTTTGCCCCCATCATTCCTGCTCCTCCTCGCCTCTTCAGGCGTTGAAGCTGGGGCTGGGACAGGGTGGACCGTCTACCCCCCACTGGCAGGCAATCTAGCCCACGCAGGTGCATCTGTCGACCTAACAATCTTTTCTCTCCACCTCGCCGGGATCTCTTCCATTCTCGGGGCCATTAACTTTATCACCACAATCCTAAACATGAAACCGCCCGCTGTCTCGCAGTATCAAACGCCCCTCTTCGTGTGGGCCGTCCTTATCACAGCCGTCCTTCTCCTTCTTTCACTGCCCGTCCTCGCCGCCGGCATCACCATGCTACTTACAGACC

>HAP81_EMLA

ATCCGCGCGGAA-CTCAGCCAGCCCGGCGCACTACTTGGAGATGACCAGATTTATAACGTAATTGTTACTGCTCACGCCTTCGTAATAATTTTCTTTATAGTAATACCAATTATGATTGGAGGGTTTGGGAACTGACTGATTCCTCTAATGATTGGGGCCCCCGACATGGCCTTCCCACGAATAAATAACATGAGCTTCTGGCTTTTGCCCCCATCATTCCTGCTCCTCCTCGCCTCTTCAGGCGTTGAAGCTGGGGCTGGGACAGGGTGGACCGTCTACCCCCCACTGGCAGGCAATCTAGCCCACGCAGGTGCATCTGTCGACCTAACAATCTTTTCTCTCCACCTCGCCGGGATCTCTTCCATTCTCGGGGCCATTAACTTTATCACCACAATCCTAAACATGAAACCGCCCGCTGTCTCGCAGTATCAAACGCCCCTCTTCGTGTGGGCCGTCCTTATCACAGCCGTCCTTCTCCTTCTTTCACTGCCCGTCCTCGCCGCCGGCATCACCATGCTACTTACAGACC

>HAP82_OSMS

ATCCGCGCGGAA-CTCAGCCAGCCCGGCGCACTACTTGGAGATGACCAGATTTATAACGTAATTGTTACTGCTCACGCCTTCGTAATAATTTTCTTTATAGTAATACCAATTATGATTGGAGGGTTTGGGAACTGACTGATTCCTCTAATGATTGGGGCCCCCGACATGGCCTTCCCACGAATGAATAACATGAGCTTCTGGCTTTTGCCCCCCTCATTCCTGCTCCTCCTCGCCTCTTCAGGCGTTGAAGCTGGGGCTGGGACAGGGTGGACCGTCTACCCCCCACTGGCAGGCAATCTAGCCCACGCAGGTGCATCTGTCGACCTAACAATCTTTTCTCTCCACCTCGCCGGGATCTCTTCCATTCTCGGGGCCATTAACTTTATCACCACAATCCTAAACATGAAACCGCCCGCTGTCTCGCAGTATCAAACGCCCCTCTTCGTGTGGGCCGTCCTTATCACAGCCGTCCTTCTCCTTCTTTCACTGCCCGTCCTCGCCGCCGGCATCACCATGCTACTTACAGACC

>HAP83_EMLA

ATCCGCGCGGAA-CTCAGCCAGCCCGGCGCACTACTTGGAGATGACCAGATTTATAACGTAATTGTTACTGCTCACGCCTTCGTAATAATTTTCTTTATAGTAATACCAATTATGATTGGAGGGTTTGGGAACTGGCTGATTCCTCTAATGATTGGGGCCCCCGACATGGCCTTCCCACGAATGAATAACATGAGCTTCTGGCTTTTGCCCCCATCATTCCTGCTCCTCCTCGCCTCTTCAGGCGTTGAAGCTGGGGCTGGGACAGGGTGGACCGTCTACCCCCCACTGGCAGGCAATCTAGCCCACGCAGGCGCATCTGTCGACCTAACAATCTTTTCTCTCCACCTCGCCGGGATCTCTTCCATTCTCGGGGCCATTAACTTTATCACCACAATCCTAAACATGAAACCGCCCGCTGTCTCGCAGTATCAAACGCCCCTCTTCGTGTGGGCCGTCCTTATCACAGCCGTCCTTCTCCTTCTTTCACTGCCCGTCCTCGCCGCCGGCATCACCATGCTACTTACAGACC

>HAP84_GATX

ATCCGCGCGGAA-CTCAGCCAGCCCGGCGCACTACTTGGAGATGACCAGATTTATAACGTAATTGTTACTGCTCACGCCTTCGTAATAATTTTCTTTATAGTAATACCAATTATGATTGGAGGGTTTGGGAACTGACTGATTCCTCTAATGATTGGGGCCCCCGACATGGCCTTCCCACGAATGAATAACATGAGCTTCTGGCTTTTGCCCCCATCATTCCTGCTCCTCCTCGCCTCTTCAGGCGTTGAAGCTGGGGCTGGGACAGGGTGGACCGTCTACCCCCCACTGGCAGGCAATCTAGCCCACGCAGGCGCATCTGTCGACCTAACAATCTTTTCTCTCCACCTCGCCGGGATCTCTTCCATTCTCGGGGCCATTAACTTTATCACCACAATCCTAAACATGAAACCGCCCGCTGTCTCGCAGTATCAAACGCCCCTCTTCGTGTGGGCCGTCCTTATCACAGCCGTCCTTCTCCTTCTTTCACTGCCCGTCCTCGCCGCCGGCATCACCATGCTACTTACAGACC

>HAP85OSMS

ATCCGCGCGGAA-CTCAGCCAGCCCGGCGCACTACTTGGAGATGACCAGATTTATAACGTAATTGTTACTGCTCACGCCTTCGTAATAATTTTCTTTATAGTAATACCAATTATGATTGGAGGGTTTGGGAACTGACTGATTCCTCTAATGATTGGGGCCCCCGACATGGCCTTCCCACGAATGAATAACATGAGCTTCTGGCTTTTGCCCCCATCATTCCTGCTCCTCCTCGCCTCTTCAGGCGTTGAAGCTGGGGCTGGGACAGGGTGGACCGTCTACCCCCCACTGGCAGGCAATCTGGCCCACGCAGGCGCATCTGTCGACCTAACAATCTTTTCTCTCCACCTCGCCGGGATCTCTTCCATTCTCGGGGCCATTAATTTTATCACCACAATCCTAAACATGAAACCGCCCGCTGTCTCGCAGTATCAAACGCCCCTCTTCGTGTGGGCCGTCCTTATCACAGCCGTCCTTCTCCTTCTTTCACTGCCCGTCCTCGCCGCCGGCATCACCATGCTACTTACAGACC

>HAP86_OSMS

ATCCGCGCGGAA-CTCAGCCAGCCCGGCGCACTACTTGGAGATGACCAGATTTATAACGTAATTGTTACTGCTCACGCCTTCGTAATAATTTTCTTTATAGTAATACCAATTATGATTGGAGGGTTTGGGAACTGACTGATTCCTCTAATGATTGGGGCCCCCGACATGGCCTTCCCACGAATGAATAACATGAGCTTCTGGCTTTTGCCCCCATCATTCCTGCTCCTCCTCGCCTCTTCAGGCGTTGAAGCTGGGGCTGGGACAGGGTGGACCGTCTACCCCCCACTGGCAGGCAATCTGGCCCACGCAGGCGCATCTGTCGACCTAACAATCTTTTCTCTCCACCTCGCCGGGATCTCTTCCATTCTCGGGGCCATTAACTTTATCACCACAATCCTAAACATGAAACCGCCCGCTGTCTCGCAGTATCAAACGCCCCTCTTCGTGTGGGCCGTCCTTATCACAGCCGTCCTTCTCCTTCTTTCACTGCCCGTCCTCGCCGCCGGCATCACCATGCTACTTACAGACC

>HAP87_DEFL

ATCCGCGCGGAA-CTCAGCCAGCCCGGCGCACTACTTGGAGATGACCAGATTTATAACGTAATTGTTACTGCTCACGCCTTCGTAATGATTTTCTTTATAGTAATACCAATTATGATTGGAGGGTTTGGGAACTGACTGATTCCTCTAATGATTGGGGCCCCCGACATGGCCTTCCCACGAATGAATAACATGAGCTTCTGGCTTTTGCCCCCATCATTCCTGCTCCTCCTCGCCTCTTCAGGCGTTGAAGCTGGGGCTGGGACAGGGTGGACCGTCTACCCCCCACTGGCAGGCAATCTAGCCCACGCAGGCGCATCTGTCGACCTAACAATCTTTTCTCTCCACCTCGCCGGGATCTCTTCCATTCTCGGGGCCATTAACTTTATCACCACAATCCTAAACATGAAACCGCCCGCTGTCTCGCAGTATCAAACGCCCCTCTTCGTGTGGGCCGTCCTTATCACAGCCGTCCTTCTCCTTCTTTCACTGCCCGTCCTCGCCGCCGGCATCACCATGCTACTTACAGACC

>HAP88_DEFL

ATCCGCGCGGAA-CTCAGCCAGCCCGGCGCACTACTTGGAGATGACCAGATTTATAACGTAATTGTTACTGCTCACGCCTTCGTAATGATTTTCTTTATAGTAATACCAATTATGATTGGAGGGTTTGGGAACTGACTGATTCCTCTAATGATTGGGGCCCCCGACATGGCCTTCCCACGAATGAATAACATGAGCTTCTGGCTTTTGCCCCCATCATTCCTGCTCCTCCTCGCCTCTTCAGGCGTTGAAGCTGGGGCTGGGACAGGGTGGACCGTCTACCCCCCACTGGCAGGCAATCTAGCCCACGCAGGCGCATCTGTCGACCTAACAATCTTTTCTCTCCACCTCGCCGGGATCTCTTCCATTCTCGGGGCCATTAACTTTATCACCACAATCCTAAACATGAAACCGCCCGCTGTCTCGCAGTATCAAACGCCCCTCTTCGTGTGGGCCGTCCTTATCACAGCCGTCCTTCTCCTTCTTTCACTGCCCGTCCTCGCGGCCGGCATCACCATGCTACTTACAGACC
